# Bidirectional association between periodontal disease and diabetes mellitus: a systematic review and meta-analysis of cohort studies

**DOI:** 10.1038/s41598-021-93062-6

**Published:** 2021-07-01

**Authors:** Julia Stöhr, Janett Barbaresko, Manuela Neuenschwander, Sabrina Schlesinger

**Affiliations:** 1 Institute for Biometrics and Epidemiology, German Diabetes Center, Leibniz Center for Diabetes Research at Heinrich Heine University Düsseldorf, Auf’m Hennekamp 65, 40225 Düsseldorf, Germany; 2 German Center for Diabetes Research, München-Neuherberg, Germany

**Keywords:** Dental diseases, Endocrine system and metabolic diseases

## Abstract

Periodontal disease has been reported to be associated with diabetes mellitus. However, the direction of the association and the influence of bias are not clear. Thus, the aim of this systematic review and meta-analysis was to summarize the existing evidence on the bidirectional prospective association between periodontal disease and diabetes mellitus by accounting for the risk of bias of the original studies. The literature search was conducted on the electronic data sources PubMed and Web of Science up to February 9th, 2021. We included observational studies, which investigated the prospective association between diabetes mellitus and periodontal disease or vice versa. The risk of bias of the primary studies was evaluated by applying the Quality in Prognosis Studies (QUIPS) tool. Random effects models were used to calculate summary relative risk (SRR) with 95% CI. Subgroup analyses were applied to investigate heterogeneity and the robustness of the finding. In total, 15 studies were included . The SRR for incident diabetes mellitus was 1.26 (95% CI 1.12, 1.41; I^2^: 71%, n = 10; participants = 427,620; identified cases = 114,361), when comparing individuals with periodontitis to individuals without periodontitis. The SRR for incident periodontitis was 1.24 (95% CI 1.13, 1.37; I^2^: 92%, n = 7; participants = 295,804; identified cases: > 22,500), comparing individuals with diabetes to individuals without diabetes. There were no significant differences between subgroups after stratification for risk of bias. The findings show a positive bidirectional association between periodontal disease and diabetes mellitus, and thus, underline the need for screening of patients with periodontitis regarding diabetes mellitus and vice versa. The main limitation of the study is the high unexplained heterogeneity between the studies including the different assessment methods of the disease diagnosis.

## Introduction

Diabetes mellitus is one of the most common chronic diseases worldwide and current estimates assume that 463 million adults were affected in 2019. The prevalence is projected to increase by almost 50% over the next years, and it is estimated that there will be about 700 million people living with diabetes by 2045^1^. Individuals with diabetes are at higher risk of developing further health-related complications and disorders, including cardiovascular disease^2^, retinopathy^3^, nephropathy^4^ and neuropathy^5^. In addition, there is indication, that patients with diabetes suffer more often from dental disease (e.g. periodontitis) compared to individuals without diabetes^6^. Periodontitis is a widespread disease, whose most severe form afflicted about 743 million people worldwide in 2010, but an underestimation is expected^7^. The disease is characterized by a chronic inflammation of the entire periodontium that can irreparably destruct the tooth-surrounding tissue and result in the resorption of the alveolar bone. Consequences such as gingival bleeding, increased tooth mobility and tooth loss can be expected^8^. Recently, a meta-analysis summarized findings on glucose disturbance, including diabetes, and periodontal disease and indicated a positive association between these two factors^9^. However, in this meta-analysis, studies with different exposures and outcomes were mixed. For example, the authors combined studies on diabetes, prediabetes and diabetes severity^10,11^. In addition, the outcome was a mixture of periodontal disease and progression of the disease^12^. Moreover, there is indication that periodontal disease is a risk factor for diabetes mellitus^13^. Both conditions are driven by inflammatory processes, which might be a possible explanation for this bidirectional association^14^. To draw clear conclusions on these associations, a systematic review and meta-analysis is needed that considers methodological challenges when combining the existing data from primary studies. First, the time sequence of exposure and outcome needs to be taken into account to obtain the direction of the association. Second, the measurement of periodontal disease differs between the studies. In observational studies, periodontal disease has been assessed as self-reported periodontitis, clinical measurements attained from oral examinations (e.g. clinical attachment loss (CAL), periodontal pocket depth (PPD)), or established scores, such as the Community Periodontal Index (CPI; based on components including gingival bleeding, dental calculus and periodontal pocket depths), or Russell’s Periodontal Index (PI; including signs of periodontal disease as inflammation, pocket formation and breakdown of function), respectively. Further, the risk of bias of the primary studies, including selection bias, information bias and confounding, should be considered by applying an appropriate tool^15^ when interpreting the data.

Thus, our aim was to conduct a systematic review and meta-analyses of the existing evidence of observational studies, which investigate prospectively the bidirectional association between periodontal disease and diabetes mellitus. In addition, we accounted for the risk of bias of the original studies, especially the assessment of periodontal disease in our meta-analyses.

## Research design and methods

The systematic literature search was conducted according to the preferred reporting items for systematic reviews and meta-analyses (PRISMA 2020) guidelines^16^. A protocol has been registered in PROSPERO (https://www.crd.york.ac.uk/prospero/display_record.php?RecordID=118829).

### Search strategy

The literature search was conducted on PubMed and Web of Science up to February 9th, 2021 to identify studies analyzing the association either between diabetes mellitus and periodontal disease, or the other direction, respectively. We used a combination of predefined search terms developed for each database without applying filters or restriction of language and calendar date (Table S1). Furthermore, we checked the reference lists of identified related reviews and included articles if eligible. Four investigators (JS, JB, MN, SS) independently screened the titles and abstracts of the identified studies in EndnoteX8 and read the full-text, if the study seemed to be relevant. Disagreements were discussed and resolved by consensus.

### Selection of studies

Studies were included, if: (1) they investigated the association between either periodontal disease and diabetes mellitus, or the other direction, respectively, (2) the association was investigated prospectively (this included prospective and retrospective cohort studies, nested case–control studies, case-cohort studies), (3) reported risk ratios (relative risk (RR), odds ratio (OR), hazard ratio (HR)) with corresponding 95% confidence intervals (CI) and 4) the papers were published following a peer-reviewed process.

Studies were excluded, if (1) they were animal studies, cross-sectional studies, case–control studies, conference abstracts or reviews, (2) they only reported crude estimates, (3) the study population was not relevant (including pregnant women or children and adolescents), (4) they focused on hyperglycemia (Fasting plasma glucose (FPG) > 110 ml/dg or haemoglobin A1c (HbA1c) > 6%) as exposure or outcome, (5) they focused on the progression of the particular disease and (6) when studies investigated potential indicators for periodontal diseases, such as tooth loss. In more detail, several studies investigated tooth loss as an indicator of periodontal disease,because assessing the number of missing teeth is less difficult and less time-consuming. However, tooth loss is not a reliable marker for periodontitis^17^, as caries is another leading cause for tooth loss^18^. Thus, we decided to exclude tooth loss as exposure/outcome, to minimize bias.

If different studies reported on similar data (same exposure and outcome), we selected the study with the largest number of participants and cases.

### Data extraction and risk of bias assessment

The extraction of the data from the studies was conducted by one investigator (JS or SS) and checked by two other investigators (JB or MN). Each inconsistency was debated until agreement was reached. The following data were extracted from each eligible study: the last name of the first author, the name of the study and the country in which it was conducted, the study design (prospective and retrospective cohort studies), the publication year, follow-up time, number of participants and cases, definition and assessment of exposure and outcome, the exposure categories and number of cases and non-cases for each category, the RRs and 95% (CI) and the variables adjusted for in the analysis.

The risk of bias of the primary studies was evaluated by applying the Quality In Prognosis Studies (QUIPS) tool^15^ by at least two independent investigators (JB, JS, MN) and discrepancies were discussed with another investigator (SS) and resolved by discussion. It includes the following 6 domains: study participation, study attrition, prognostic factor measurements, outcome measurements, study confounding and statistical analysis, and reporting. In each domain, the studies were evaluated for their reliability and eligibility and each domain was rated as low, moderate or high risk of bias. Finally, an overall risk of bias for each primary study was determined, whereby the domains prognostic factor and outcome measurements were assigned a higher weight, as we considered these domains to be of decisive importance. Thus, if a study was rated with high risk in one of these domains, the overall risk of bias was also judged as high. In this context, assessment of periodontal disease was judged as low risk of bias if clinical measurements of CAL or PPD were obtained, or ICD-codes based on the previously valid classification system for periodontal disease by Armitage (according to CAL) were used^19^. Further, we considered the CPI, introduced by the World Health Organization (WHO), as appropriate method, since it is faster and more reproducible in large epidemiological studies. The extend of PPD is the main factor for establishing the code and thus provides an indication for CAL^20^. A self-reported diagnosis that is validated by a dentist, classification by the PI and ICD-codes from a health insurance database, without further specification, were judged with moderate risk of bias. Self-reported diagnosis without validation is rated with high risk. The assessment of the diabetes mellitus diagnosis was classified with low risk of bias, if one of the following diagnostic tests was conducted, in accordance with the criteria of the American Diabetes Association: values of HbA1c or of plasma glucose (FPG or 2-h FPG after an oral glucose tolerance test)^21^. If a study included a self-reported diagnosis, validated by a physician or on ICD-codes from a health insurance database, it was considered to be at moderate risk. Diagnosis based on self-reports without validation were rated with high risk. For the domain study confounding, we defined the following confounders as important, for which a study should have at least been adjusted: age, sex, body mass index (BMI)/overweight, smoking status and socio-economic status. This domain could not be rated higher than moderate risk of bias, because residual confounding cannot be completely excluded in observational studies. In detail, the signaling questions for the QUIPS tool can be found in Table S2.

### Statistical analysis

Meta-analyses were conducted for the following associations: (i) periodontal disease as exposure and incidence of diabetes mellitus as outcome, (ii) diabetes mellitus as exposure and incidence of periodontal disease as outcome. Summary relative risks (SRR) and 95% CIs for these associations were calculated by using random effects meta-analysis by DerSimonian and Laird^22^. In addition, we conducted dose–response meta-analyses for exposure on periodontitis, if it was measured as continuous variables (CPI or PPD), and studies provided findings (RRs and 95% CIs) for at least three quantified categories. For this, we calculated the study-specific slopes and corresponding 95% CIs from the natural logarithm of the reported RRs and 95% CIs across the exposure categories^23^. The shapes of the relationships were evaluated by using restricted cubic spline regression models, and a likelihood ratio test was used to test for non-linearity^24^.

Heterogeneity was evaluated by applying I^2^, tau^2^, and 95% prediction intervals (95% PIs), and was investigated in subgroups analyses. Subgroup meta-analyses were conducted by total risk of bias, risk of bias for the exposure and outcome domains (high, moderate, low), sex, geographic locations, type of diabetes, duration of follow-up, number of cases, smoking status, and by adjustment of the original studies for potentials confounders: education, smoking status, overweight, fruit and vegetable intake, alcohol intake, physical activity, other chronic diseases. Differences were tested by using meta-regression^25^. Small study effects and publication bias were assessed by using Egger’s test and by visual inspection of funnel plots^26^, if more than 10 studies were included in the meta-analysis^27^. Potential publication bias was indicated by asymmetry of the funnel plot and a *p* value < 0.01 for Egger’s test. All statistical analyses were conducted using Stata version 15.1 software (StataCorp, texas, US).

### Certainty of evidence assessment

We evaluated the certainty of evidence for each association using the updated Grading of Recommendations Assessment, Development and Evaluations (GRADE). The GRADE tool covers the following aspects: the study design, the risk of bias of the primary studies, imprecision of the findings, inconsistency between the primary studies, indirectness in the primary studies, publication bias, the magnitude of effect of the pooled findings, indication for dose–response relations and the impact of residual confounding. The certainty of evidence can be evaluated as high, moderate, low or very low. A high certainty of evidence means that it is very likely that the effect estimate lies close to the true effect, whereas a very low certainty of evidence means that it is very likely that the inclusion of future studies will change the estimate.

## Results

In total, 9384 studies were initially identified, and after removing 2427 duplicates and excluding 6411 articles by title and abstract, 546 were investigated in a full-text analysis (Figure S1). Finally, 15 cohort studies were included in our meta-analysis, the characteristics of all included studies are presented in the Supplemental Material (Table S4). A list of the excluded studies and the corresponding reasons are shown in the supplement (Table S3).

### Periodontal disease and incidence of diabetes mellitus

We identified 10 studies that investigated the association between periodontal disease and incidence of diabetes mellitus with a total of 427,620 participants and 114,361 identified cases of diabetes mellitus over a mean follow-up period of 9.9 years (range 5–17 years)^13,28–35^. Four studies achieved a low, three of them a moderate, and three a high overall risk of bias (Table S5). The assessment of periodontal disease was evaluated with low risk of bias in seven studies, moderate in one study, and high in two studies. The assessment of diabetes was judged as low risk of bias in seven, moderate in two studies, and high in one study. Eight studies focused on type 2 diabetes^13,28,31–35^, one on both types^30^, and for one it remains unclear which type of diabetes was investigated^29^.

**Figure 1:**
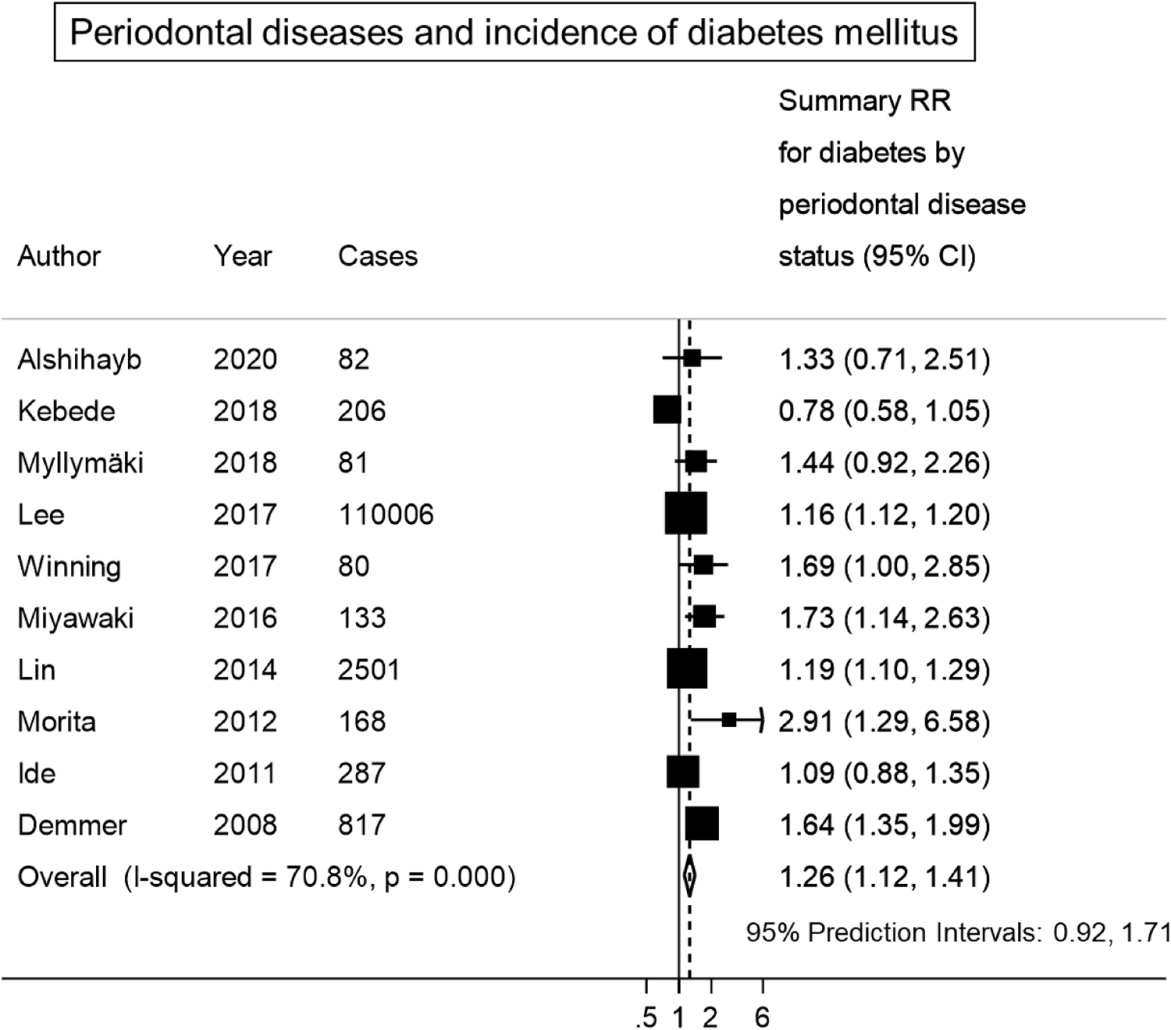
Meta-analysis of periodontal disease and incidence of diabetes mellitus.

**Figure 2:**
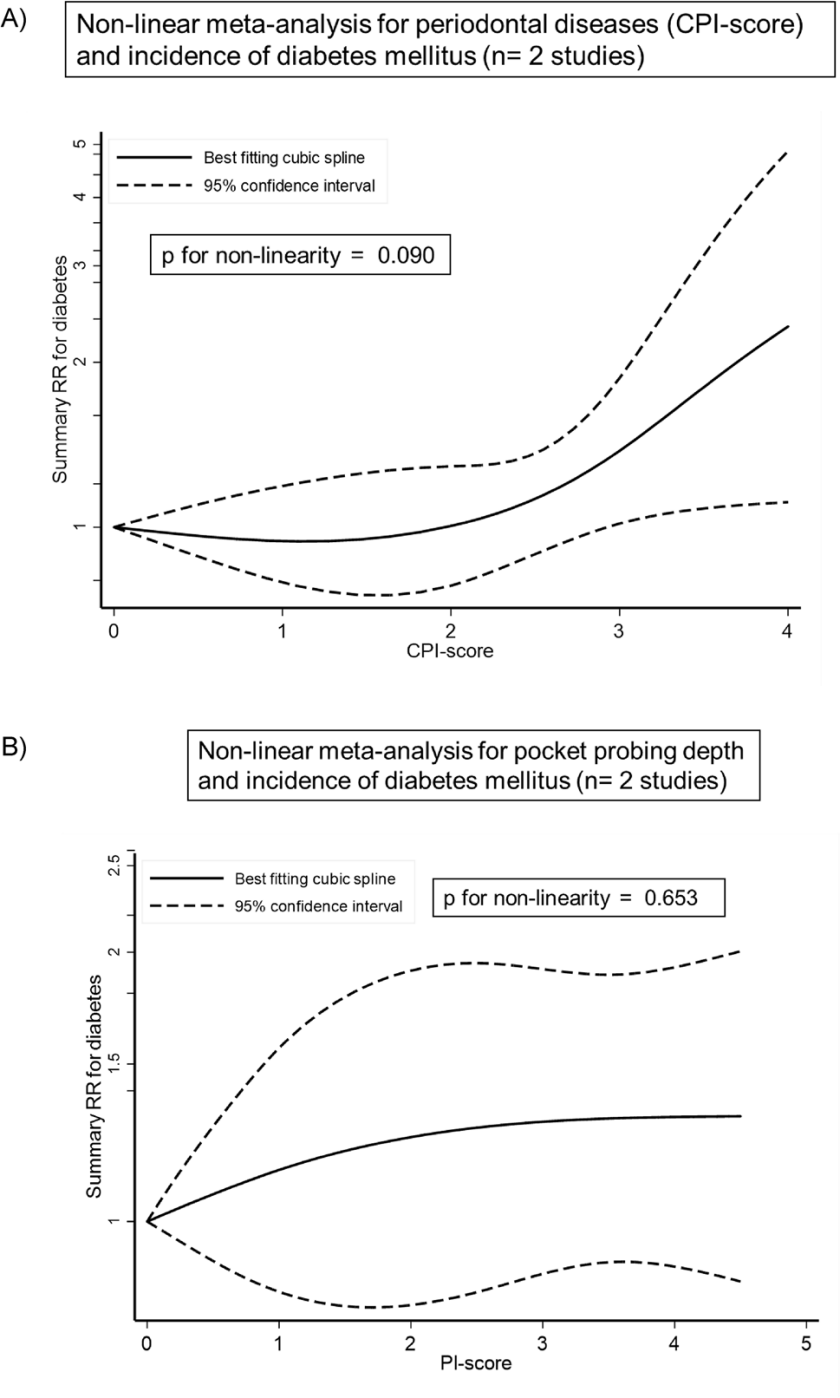
Non-linear dose–response meta-analysis for periodontal disease, defined as (**A**) CPI-score and (**B**) defined as PPD, and incidence of diabetes mellitus.

The SRR for incident diabetes mellitus was 1.26 (95% CI 1.12, 1.41; I^2^:71%, Tau^2^: 0.014, 95% PI: 0.92, 1.71), when comparing individuals with periodontitis to individuals without (Fig. 1). There was indication for a non-linear trend for the relationship between the CPI score and the incidence of diabetes. No association was observed for a CPI score ≤ 2, but an increase in the incidence of diabetes was observed after a CPI score ≥ 3 (score 3: SRR (95% CI): 1.38 (1.02, 1.87); score 4: SRR (95% CI): 2.33 (1.11, 4.87); p for non-linearity:0.090; n = 2) (Fig. 2A). The non-linear analysis for the relationship between PPD and the incidence of diabetes showed an increased relative risk for diabetes up to a PPD of 3 mm, then the graph reached a plateau with no further increase in risk (PPD 1.0 mm: SRR (95% CI): 1.15 (0.83, 1.59); PPD 2.5 mm: SRR (95% CI): 1.27 (0.83, 1.95), PPD 3,5 mm: SRR (95% CI): 1.30 (0.90, 1.89), and PPD 4,5 mm: SRR (95% CI): 1.31 (0.86, 2.01), based on n = 2; p for non-linearity: 0.653) (Fig. 2B). The certainty of evidence was judged as moderate for the association between periodontal disease and incidence of diabetes mellitus (Table S6).

### Diabetes mellitus and incidence of periodontal disease

The seven studies on diabetes mellitus and incidence of periodontitis included 295,804 participants and > 22,500 diagnosed cases of periodontal disease (missing information on cases for Lee et al*. *^36^)^33,36–41^. The mean follow-up period was 11.1 years (range 5–20 years). The overall risk of bias was low in one study, moderate in four studies, and high in two studies (Table S5). Two studies were rated as low risk of bias regarding the exposure assessment, four as moderate and one study with high. Four studies focused on type 2 diabetes^37,38,40,41^, one on type 1 diabetes^39^, one on both types of diabetes^36^, and in one it was not specified^33^. Outcome assessment was rated as low risk of bias in four studies, moderate for two studies, and high for one study.

**Figure 3:**
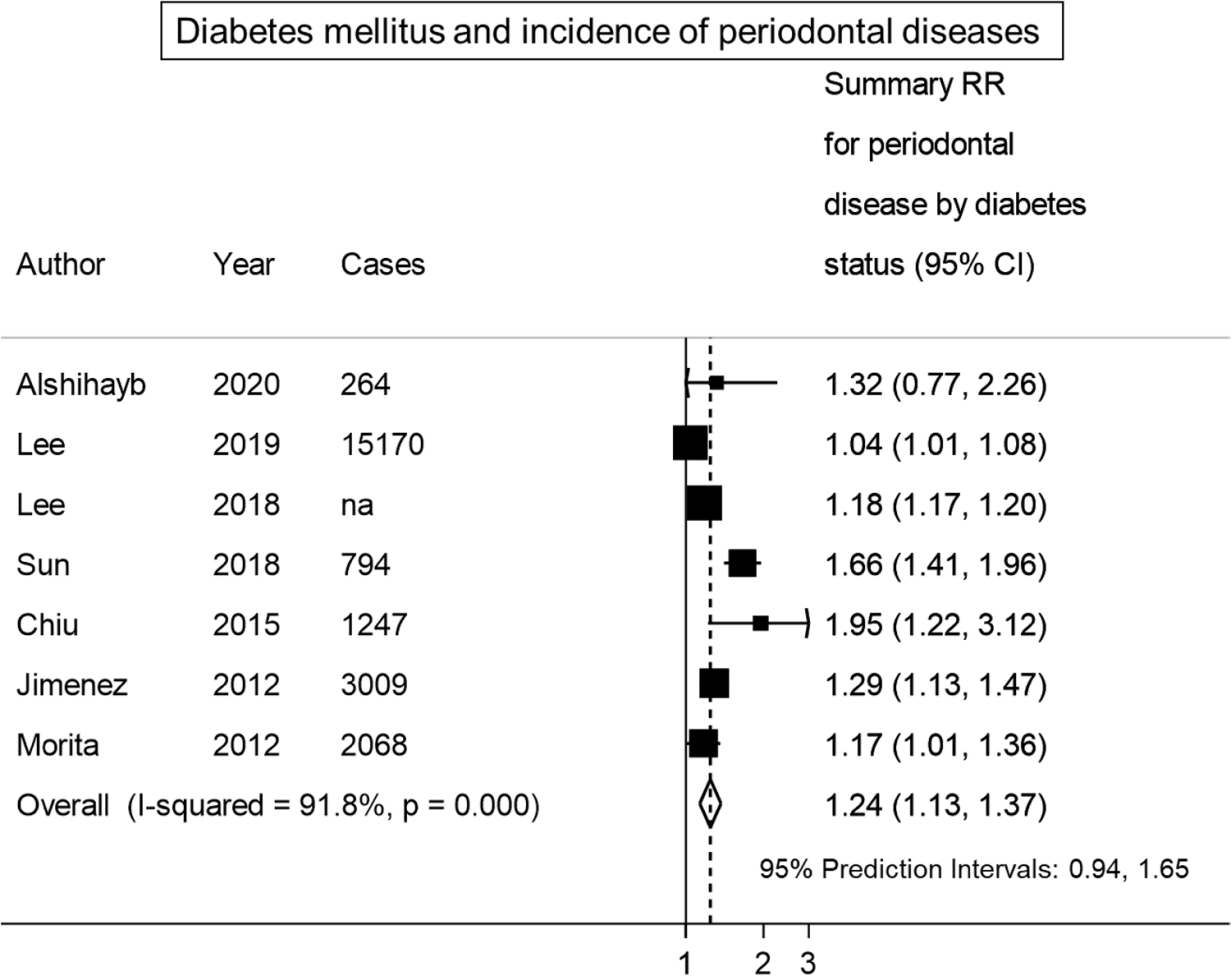
Meta-analysis of diabetes mellitus and incidence of periodontal disease.

The SRR for incident periodontitis was 1.24 (95% CI 1.13, 1.37; I^2^: 92%, tau^2^: 0.01, 95% PI 0.94, 1.65), comparing individuals with diabetes to individuals without diabetes (Fig. 3). The certainty of evidence was evaluated as moderate for the association between diabetes mellitus and incidence of periodontal disease (Table S6).

### Subgroup analyses

There was no significant heterogeneity between subgroups after stratification by overall risk of bias, risk of bias in the exposure and outcome domains, sex, geographical location, type of diabetes, duration of follow-up, number of included cases, adjustment for important confounders (e.g. smoking status, education, etc.) (Table S7–S8). Publication bias was only evaluated for the association between periodontal disease and incidence of diabetes mellitus because more than ten studies were available for these association, and no indication for publication bias was observed by visual inspection of the funnel plot (Figure S2) and Egger’s test (*p* = 0.194).

## Discussion

This systematic review and meta-analysis of 15 cohort studies showed that there was a positive bidirectional association between both periodontal disease and diabetes mellitus with a moderate certainty of evidence. For patients with diabetes, the data indicated a 24% (95% CI 13%, 37%) increase in the incidence of periodontal disease. For patients with periodontitis, the relative risk of developing diabetes mellitus was elevated by 26% (95% CI 12%, 41%). These results coincide with those of the meta-analysis by Nascimento et al., where an 86% (95% CI 30%, 180%) increased relative risk of periodontitis for individuals with diabetes was found. The fact that the relative risk is higher than in our analysis may be due to the fact that different degrees of severity of diabetes were taken into account as exposure and the outcome was not only incidence of periodontitis but also progression of the disease and its marker^9^. In contrast, the meta-analysis by Ziukaite et al. focused on the other direction of the association and reported a 27% (95% CI 11%, 74%) higher prevalence of diabetes for patients with periodontitis^42^, which is comparable to our findings. Risk of bias was high in the previous meta-analyses because different study designs, different definitions of exposure and outcomes were combined. In our meta-analysis, we considered both directions separately, included only prospective studies and did not mix disease status with pre-disease status or progression of the diseases, respectively.

In the risk of bias assessment of the individual studies, we particularly focused on the assessment and definition of periodontal disease and diabetes. In general, the diagnosis of a disease was defined by self- reports, clinical examinations or a combination of both. While a meta-analysis showed that the prevalence for diabetes was much lower for self-reported periodontitis compared to clinical periodontal measurements^42^, our meta-analyses did not show a significant difference here. However, subgroup meta-analyses relied on small numbers of studies and more studies with accurate assessment methods are needed. Although it was rarely applied among the studies, assessment via CAL has been considered the gold standard for the classification of chronic periodontitis^19^. But with this method measurement errors may occur especially in the initial phase of periodontitis^43^, for example, if the manual probe is applied with incorrect force, it can be advanced into intact attachment fibers^44^. It should also be taken into account that these measurements are associated with a standard deviation of 0.8–1.07 mm, even for experienced investigators^45^. Another applied assessment method is the determination of the PPD. While the assessment is more simple than the CAL, it has been shown that this method used alone, can lead to an underestimation of cases^46^ because, especially with increasing age, the extent of PPD no longer correlates with CAL^47^. In addition, there are studies that used indices such as Russell's PI^28^, or the CPI^13,33,37^ to classify the disease. Periodontitis diagnosis based on the PI is critical because this index is only visually assessed and does not include clinical measurements, and it includes gingivitis as an early form of periodontitis^48^. Although the CPI is characterized by its reproducibility and simplification^49^, it is not considered sufficient to describe the extent of periodontal disease^50^. In summary, the assessment and definition of periodontal disease vary widely across studies and there are no consistent thresholds for CAL/PPD and numbers of affected teeth to determine whether the disease is present or not. The Division of Oral Health at the Centers for Disease Control and Prevention in collaboration with the American Academy of Periodontology has provided a definition that combines measurements of CAL and PPD to assess periodontal disease to avoid misinterpretation of the periodontal status^48^. In order to make clear statements about the association between periodontitis and diabetes mellitus, it should be ensured in the future studies that established assessment of periodontitis is applied, and thus, enables comparability between studies.

There are many possible explanations for the observed bidirectional associations between periodontitis and diabetes, which are related to inflammatory processes. For example, on the one hand, untreated diabetes mellitus, both type 1 or 2 diabetes, lead to metabolic disorders caused by hyperglycemia^51^. Poor glycemic control in patients with diabetes has been shown to raise the level of systemic inflammation markers, e.g. interleukin-1ß, in the gingival crevicular fluid of a periodontal pocket^14^, which is associated with the onset and severity of periodontal disease^52^. On the other hand, it has been show that gram-negative bacteria in the periodontal pockets elevate serum inflammatory markers such as c-reactive protein^53^. This can induce hyperinflammatory immune cells and promote the release of proinflammatory cytokines, which lead to insulin resistance^54^.

Strengths of our meta-analysis is the bidirectional investigation and the dose–response meta-analysis between periodontal disease and diabetes mellitus by including only prospective studies and accounting for the major risk of bias sources, namely assessment of exposures and outcomes, and other sources of bias e.g. selection of participants and confounding. However, there are also limitations in our meta-analysis. First, as already described in detail, risk of bias regarding the assessment of periodontitis could not be ruled out in all of the studies. To account for this, we conducted subgroup analysis and did not observe significant differences. However, these analyses were based on small numbers of studies, and should be treated with caution. Second, most of the included studies did not clearly differentiate between type 1 and type 2 diabetes. In the subgroup meta-analysis by type of diabetes, we did not observe substantial differences, but again, the number of studies in the subgroups were small. Third, residual confounding cannot be excluded because of the observational study design of the included studies. Fourth, the included studies did not report on repeated measurements and thus, we could not account for changes of the disease status (impairment or also improvement due to treatment). Fifth, we did not search for grey literature, because we prefer to include only peer-reviewed studies. Last, we identified high heterogeneity between studies, which may arise from different methodological aspects e.g. different assessment methods of periodontal disease and/or diabetes. However, we conducted several subgroups analyses and meta-regression for total risk of bias and risk of bias of exposure and outcome assessment, further factors such as geographic location, and methodological aspects (number of cases, duration of follow-up, adjustment for specific confounders etc.) and heterogeneity remained unchanged.

Our comprehensive meta-analysis can help investigators to plan and conduct further research regarding bidirectional associations between periodontal disease and diabetes mellitus and can help for decision making in the clinical context. Our findings support existing guidelines for physicians and dentists regarding the screening of patients with diabetes, which recommend that every new confirmed patient with diabetes mellitus, should be informed that there is an increased risk of developing periodontal disease and that glycemic control is more difficult in this case. Thus, every initial examination should include a periodontal evaluation^55^. It has been shown, that 40.7% of the dental patients without a diagnosis of diabetes (< 45 years) had HbA1c values around 5,7% or higher^56^, thus, screening for diabetes mellitus in the dental office is as important.

In conclusion, there was a bidirectional association between periodontal disease and diabetes mellitus, even after stratifying for major risk of bias. However, only few studies with low risk of bias were available. To strengthen these findings more studies with valid assessment of periodontal diseases and diabetes are needed. The findings support current guidelines that patients with periodontitis should be screened for diabetes mellitus, and that patients with diabetes mellitus should be informed about their higher risk of developing periodontal diseases.

## Supplementary Information

Supplementary Information.

## Data Availability

All data are available in the manuscript and its Supplement file.

## Abbreviations

BMI: Body mass index
CAL: Clinical attachment loss
CPI: Community periodontal index
CI: Confidence interval
FPG: Fasting plasma glucose
Hb A1c: Haemoglobin A1c
HR: Hazard ratio
ICD: International classification of disease
OR: Odds ratio
PI: Periodontal index
PPD: Periodontal pocket depths
PI: Predication interval
QUIPS: Quality in prognosis studies
RR: Relative risk
SRR: Summary relative risk
WHO: World health organization
