# Supplementary material for: Bidirectional association between periodontal disease and diabetes mellitus: a systematic review and meta-analysis of cohort studies
Source: Sci Rep. 2021 Jul 1;11:13686. doi: 10.1038/s41598-021-93062-6 (PMC8249442; doi:10.1038/s41598-021-93062-6)
Supplement: Supplementary file 1 — Supplementary Information. [file 41598_2021_93062_MOESM1_ESM.pdf]

**Supplemental Material**

**Bidirectional association between periodontal disease and diabetes mellitus: A systematic review and meta-analysis of cohort studies**

Julia Stöhr[1], Janett Barbaresko[1], Manuela Neuenschwander[1,2], Sabrina Schlesinger[1,2]

[1] Institute for Biometrics and Epidemiology, German Diabetes Center, Leibniz Center for Diabetes Research at Heinrich Heine University Düsseldorf, Düsseldorf, Germany

[2] German Center for Diabetes Research, München-Neuherberg, Germany.

Corresponding author:

Dr. Sabrina Schlesinger, Institute for Biometry and Epidemiology, German Diabetes Center, Auf'm Hennekamp 65, 40225 Düsseldorf, Germany. Phone: +49 211 3382 231. Fax: +49 211 3382 677. Email: sabrina.schlesinger@ddz.de.

ORCID: 0000-0003-4244-0832

**Table S1.** Search term

| |
|---|
| **a) Pubmed (all fields)** |
| ((periodontal OR periodontitis OR "periodontal disease" OR “clinical attachment loss”) AND (diabetes OR Hba1c OR HbA1c OR A1c OR glycated OR glycosylated OR glucose OR "Glycated Hemoglobin A")) |
| |
| **b) Web of Science (title, abstract, author keywords, keywords plus®)** |
| **TOPIC**: (((periodontal OR periodontitis OR "periodontal disease" OR “clinical attachment loss”) AND (diabetes OR Hba1c OR HbA1c OR A1c OR glycated OR glycosylated OR glucose OR "Glycated Hemoglobin A"))) |

**Table S2**. Quality assessment using the Quality in Prognosis studies (QUIPS) tool.

| **Signalling question** | **Authors‘ judgement for `yes** |
|---|---|
| **Study participation: yes/no/unclear/NA (not applicable)** | |
| a) Adequate participation in the study by eligible people | 1) men and women ≥18 years without diabetes at baseline are eligible or<br>2) men and women ≥18 years without periodontal disease at baseline are eligible |
| b) Description of the source population or population of interest | Source population or population of interest is clearly described (e.g. region, age ...) |
| c) Description of the baseline study sample | Baseline study sample is clearly described |
| d) Adequate description of the sampling frame and recruitment | Recruitment, selection criteria and key characteristics of the source population are clearly described |
| e) Adequate description of the period and place of recruitment | Time period and place of recruitment are clearly described |
| f) Adequate description of inclusion and exclusion criteria | Inclusion and exclusion criteria are clearly described and presented. |
| **Study participation: risk of bias rating (low/moderate/high/unclear)** | **Low**: The study sample represents the population of interest and sufficient information on the recruitment and the selection process and the key characteristics of the population is available.<br>**Moderate:** The study sample represents the population of interest but some information on the recruitment and/or the selection process and/or the key characteristics of the population were not described.<br>**High:** The study sample does not represent the population of interest and/or insufficient information on the recruitment and the selection process and the key characteristics of the population is available.<br>**Unclear:** not sufficient information were provided to judge this domain. |
| **Study attrition: Yes/no/unclear/NA** | |
| a) Adequate response rate for study participants | Response rate was adequate: ≥80% (<5 years) or ≥70% (≥5 years) |
| b) Attempts to collect information on participants who dropped out described | Attempts to collect information on participants who dropped out are described (e.g. telephone contact, mail, registers) |
| c) Reasons for loss to follow-up provided | Information about the reason participants were lost to follow-up are set up (e.g. deceased participants, participants refused or moved) |
| d) Adequate description of participants lost to follow-up | Key characteristics of participants lost to follow-up are described (Age, sex, diabetes status/ periodontal status at baseline) |
| e) No important differences between participants who completed the study and those who did not | No important differences in baseline characteristics between responders and non-responders |

| | |
|---|---|
| **Study attrition: risk of bias rating (low/moderate/high/unclear)**<br><br>**Note:** In this domain, the first item was rated as the most important and thus decisively weighted | **Low:** If loss of follow-up was low and/or loss to follow-up was not associated with key characteristics and no potential bias of the observed association between the prognostic factor and the outcome is expected.<br>**Moderate:** If loss of follow-up was low but not sufficient information were provided to judge if remaining loss of follow-up was associated with key characteristics, and thus, potential bias of the observed association between the prognostic factor and the outcome cannot be ruled out completely.<br>**High:** If loss of follow-up was high and loss to follow-up was associated with key characteristics and potential bias of the observed association between the prognostic factor and the outcome is expected.<br>**Unclear:** not sufficient information were provided to judge this domain. |
| **Prognostic factor measurements: yes/no/unclear/NA** | |
| a) Clear definition or description provided | 1) Clear definition of periodontitis has been set by clinical oral examination (e.g. measurements of PPD or CAL) or<br>2) Clear definition of diabetes mellitus has been set by blood laboratory measurements (e.g. FPG, HbA1c values) |
| b) Adequately valid and reliable method of measurement | 1) Valid and reliable methods of assessment for periodontitis include measurements of CAL or PPD and can be presented by Community Periodontal Index;<br>We consider self-reported diagnosis that is validated by a physician and ICD-Codes from health insurance data as partial valid and reliable;<br>Percentage of radiographic bone loss or self-reported periodontal disease or self-reported symptoms of periodontal disease without validation are not reliable;<br>Technique for measurements of periodontal status is well described and measurements were performed by a dentist<br>2) Measurements of blood glucose or HbA1c levels are valid and reliable methods of measurement for diabetes mellitus;<br>Self-reported history of diabetes with validation of the diagnosis by a physician and information from medical records are partial reliable;<br>Only self-reported diagnosis without validation by a physician is not considered a valid and reliable method of measurement;<br>Technique for laboratory measurements is well described. |
| c) Continuous variables reported or appropriate cut points used | 1) Standard cut points for diagnosis of periodontitis: Probing pocket depth ≥ 4mm which corresponds to Community Periodontal Index code ≥ 3; |

| | |
|---|---|
| | clinical attachment loss: 1-2mm (mild); 3-4mm (moderate); ≥5mm (severe) or<br>2) Standard cut points for diagnosis of diabetes mellitus: Fasting plasma glucose ≥ 126 mg/dL or HbA1c >6,5%. |
| d) Same method and setting of measurement used in all study participants | 1) Measurements of periodontal status are the same for all study participants<br>2) Measurements of glycemic status are the same for all study participants. |
| e) Adequate proportion of the study sample had complete data | Number in final model with complete data |
| f) Appropriate methods of imputation were used for missing data | Complete case analysis for exposure and outcome variables are required. If the analysis was almost complete case analysis, imputation was not required |
| **Prognostic factor measurements: risk of bias rating (low/moderate/high/unclear)** | **Low:** The prognostic factor of interest is adequately defined and measured in an adequate proportion of the study sample to sufficiently limit potential bias.<br>**Moderate:** The prognostic factor of interest is partly adequately defined and measured in an adequate proportion of the study sample<br>**High:** The prognostic factor of interest is not adequately defined and/or measured and/or missing in a substantial proportion of the study participants leading to potential bias.<br>**Unclear:** not sufficient information were provided to judge this domain. |
| **Outcome measurement: yes/no/unclear/NA** | |
| a) Clear definition of the outcome provided | 1) Clear definition for the diagnosis of diabetes mellitus has been set (e.g. FPG levels, HbA1c values)<br>2) Clear definition for the diagnosis of periodontitis has been set (e.g. PPD or CAL levels) |
| b) Use of adequately valid and reliable methods of outcome measurement | 1) Valid and reliable methods of assessment for periodontitis include measurements of CAL or PPD and can be presented by Community Periodontal Index;<br>We consider self-reported diagnosis that is validated by a physician and ICD-Codes from health insurance data as partial valid and reliable;<br>percentage of radiographic bone loss or self-reported periodontal disease without validation are not reliable;<br>Technique for measurements of periodontal status is well described<br>2) Measurements of blood glucose or HbA1c levels are valid and reliable methods of measurement for diabetes mellitus;<br>Self-reported history of diabetes with validation of the diagnosis by a physician and information from medical records are partial reliable;<br>Only self-reported diagnosis without validation by a physician is not considered a valid and reliable method of measurement; |

| | |
|---|---|
| | technique for laboratory measurements is well describe |
| **c)** Use of same method and setting of outcome measurement in all study participants | 1) Measurements of type 2 diabetes mellitus are the same for all study participants or<br>2) Measurements of periodontal disease are the same for all study participants |
| **Outcome measurement: risk of bias rating (low/moderate/high/unclear)** | **Low:** The outcome of interest is adequately defined and measured in an adequate proportion of the study sample to sufficiently limit potential bias.<br>**Moderate:** The outcome of interest is partly adequately defined and measured in an adequate proportion of the study sample<br>**High:** The outcome of interest is not adequately defined and/or measured and/or missing in a substantial proportion of the study participants leading to potential bias.<br>**Unclear:** not sufficient information were provided to judge this domain. |
| **Study confounding: yes/no/unclear/NA** | |
| a) Measurement of all important confounders | Minimal adjusted models should include: Age, sex, socio-economic status, smoking and BMI as most important confounders |
| b) Provision of clear definitions of the important confounders measured | Measurements of confounders are described and defined |
| c) Adequately valid and reliable measurements of all important confounders | Measurements of confounding factors are valid and reliable (e.g. validated questionnaires, examination by trained personal, etc.) |
| d) Use of the same method and setting of confounding measurement in all study participants | The method and setting of confounding measurement are the same for all participants |
| e) Appropriate imputation methods used for missing confounders (if applicable) | Appropriate methods of imputation for missing covariate data are applied and described. |
| f) Important potential confounders were accounted for the study design | Important potential confounders are accounted for in the study design (e.g., matching for key variables, stratification, use of multivariable analysis etc.). |
| g) Important confounders were accounted for in the analysis | Important potential confounders are accounted for in the analysis (i.e., appropriate adjustment). |
| **Study confounding measurement: risk of bias rating (low/moderate/high/unclear)**<br><br>**Note:** Confounding is expected in all observational studies, because residual confounding cannot be completely excluded. Thus, for the item a) no study was assigned with “yes” and the total domain cannot be rated higher than moderate | **Low:** Important potential confounders were appropriately accounted for, and no potential bias of the observed association between the prognostic factor and the outcome is expected.<br>**Moderate:** Studies adjusted for the defined minimal adjustment set and confounders were adequately defined and measured to sufficiently limit potential bias.<br>**High:** Studies did not adjust for the defined minimal adjustment set and/or confounders were adequately defined and measured leading to potential bias.<br>**Unclear:** not sufficient information were provided to judge this domain. |
| | |

| Statistical analysis and reporting: yes/no/unclear/NA | |
|---|---|
| a) Sufficient presentation of data to assess the adequacy of the analytic strategy | There is sufficient presentation of data to assess the adequacy of the analysis (e.g. findings are displayed in a table or in the text). The results are expressed as risk ratios (e.g. hazard ratios, relative risks, odds ratios) with corresponding 95% confidence intervals |
| b) Strategy for model building is appropriate and based on conceptual framework model | The selection of the confounders are described and appropriate (e.g. literature-based); step-wise regression is not appropriate |
| c) Statistical model is adequate for the study design | Multivariable logistic regression or cox proportional hazard model are applied. Univariate methods are not appropriate. |
| d) No selective reporting of results | There is no selective reporting of results (e.g. findings are shown for a specify age group, time period etc.) |
| **Statistical analysis and reporting: risk of bias rating (low/moderate/high/unclear)** | **Low:** The statistical analysis was appropriate and the data are sufficiently reported.<br>**Moderate:**. The statistical analysis was appropriate but the strategy for model building is not adequately described and/or for the main findings there is no selective reporting of the data.<br>**High:** The statistical analysis was not appropriate and/ or there is selective reporting of the data.<br>**Unclear:** not sufficient information were provided to judge this domain. |

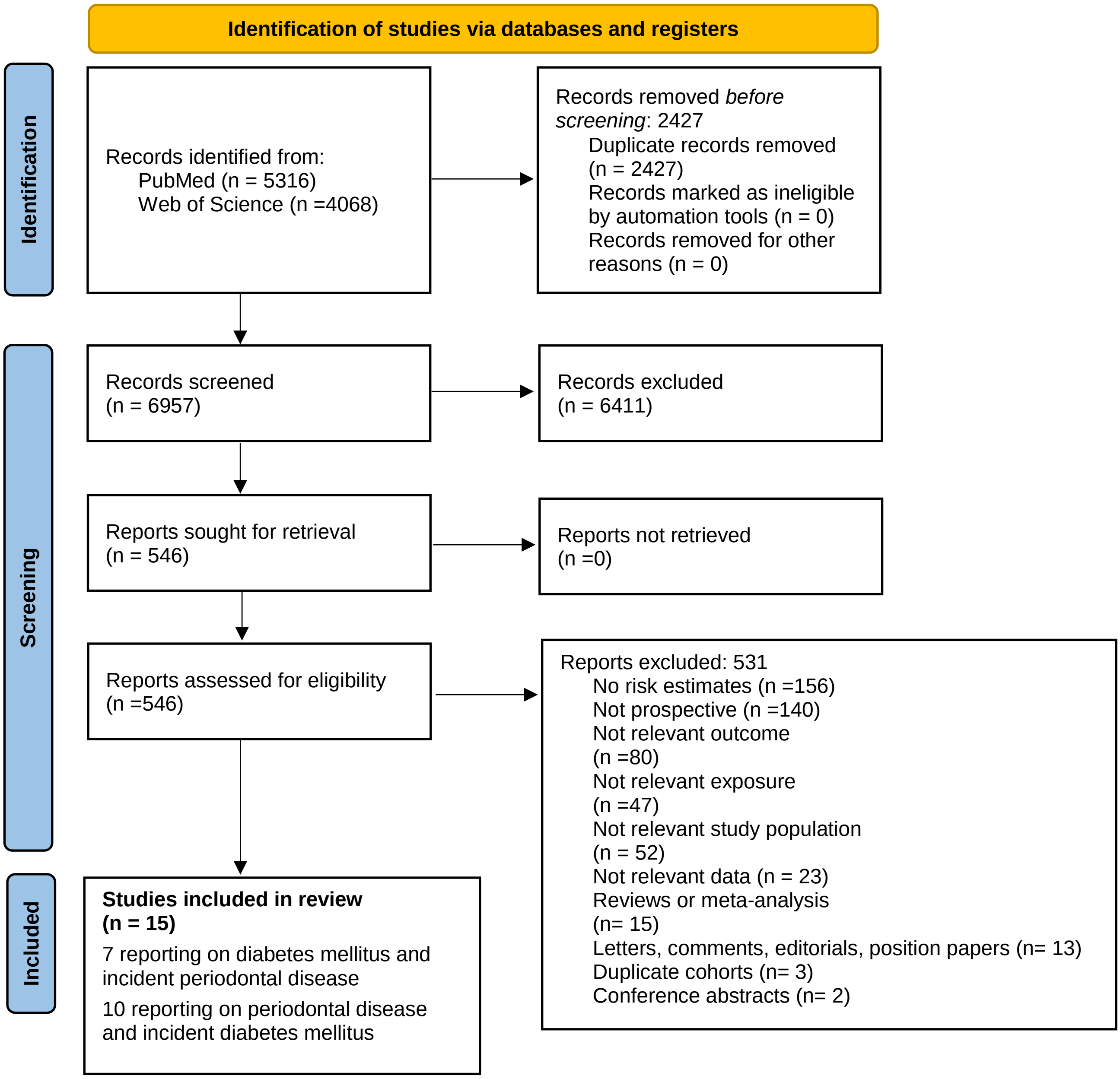


**Figure S1.** Flow chart of the study selection process

*Template from:* Page MJ, McKenzie JE, Bossuyt PM, Boutron I, Hoffmann TC, Mulrow CD, et al. The PRISMA 2020 statement: an updated guideline for reporting systematic reviews. BMJ 2021;372:n71. doi: 10.1136/bmj.n71.

**Table S3**. Excluded studies based on full-text screening

| **Exclusion criteria** | **Excluded studies** |
|---|---|
| No risk estimates (studies that do not have a risk ratio of the association of periodontal disease and diabetes mellitus) | [1-156] |
| Not prospective (cross-sectional studies, case-controls studies, no prospective associations) | [157-296] |
| Not relevant outcome (studies that do not have an indicator of periodontal disease or diagnosis of diabetes mellitus as outcome) | [297-376] |
| Not relevant study population (e.g. studies in children and adolescents, pregnant women) | [377-428] |
| Not relevant exposure (studies that do not have an indicator of periodontal disease or diagnosis of diabetes mellitus as exposure) | [429-475] |
| Not relevant data (studies that do not have a relevant exposure and outcome) | [476-498] |
| Systematic reviews/Meta-analysis | [499-513] |
| Letter, comments, editorials, position paper | [514-526] |
| Duplicate cohorts | [527-529] |
| Conference abstracts | [530, 531] |

**Table S4**. Study characteristics of cohort studies included in the meta-analysis

| First author, publication year, country | Study design, Study name or description, Follow-up | Sex, age | Number of participants, number of cases | Exposure, exposure assessment | Exposure categories | Outcome, outcome assessment | Relative Risk (95% CI) | Adjustment for confounders |
|---|---|---|---|---|---|---|---|---|
| **Studies of diabetes mellitus and incident periodontal diseases** | | | | | | | | |
| Alshihayb et al., 2020 USA[532] | Prospective cohort study, Veterans Affairs (VA) Dental Longitudinal Study (DLS) is a subset of the VA Normative Aging Study (NAS), 14 y | Men, 58.9y | 521, 264 | Type 2 diabetes, existing NAS medical records | Diabetes yes/no | Incident periodontal Disease (severe), Clinical periodontal examinations: probing pocket depth at multiple sites per tooth & attachment loss | 1.32 (0.77; 2.26) | Time-varying age, education, time-varying body mass index, time-varying comprehensive smoking index |
| Chiu et al., 2015, Taiwan[533] | Prospective cohort study, Keelung community-based integrated screening (KCIS) program, 5 y | Women and men, 35-44 y | 4387, 1247 | Type 2 diabetes, Fasting plasma glucose (FPG) or self-reported type 2 diabetes | FPG normal (<100 mg/dL), Type 2 diabetes (≥126 mg/dL) | Incident periodontal disease, measurement of periodontal pocket depth (PPD) and classified by Community Periodontal Index (CPI) | 1.95 (1.22; 3.13) | Age, gender, years of education, betel nut chewing, smoking, alcohol consumption, waist, triglyceride, HDL, elevated blood pressure, fruit intake, physical activity |
| Jimenez et al., 2012, USA[534] | Prospective cohort study Health Professionals Follow-Up Study (HPFS), 20 y | Men, 40-75 y | 35247, 3009 | Type 2 diabetes, self-reported: 1) ≥1classic symptom of diabetes and FPG of ≥140mg/dl (cases reported after 1996: ≥ 126mg/dl), non-FPG | Diabetes yes/no | Periodontal disease, Self-reported | 1.29 (1.13; 1.47) | Age, gender, smoking status, BMI, fruit/vegetable intake, number of teeth at baseline, physical activity, alcohol |

| | | | | | | | | |
|---|---|---|---|---|---|---|---|---|
| | | | | ≥200mg/dl;<br>or 2) elevated plasma glucose concentrations on at least two different occasions in the absence of symptoms;<br>or 3) hypoglycemic | | | | consumption, dental profession, baseline history of stroke, coronary artery bypass surgery or myocardial infarction |
| Morita et al., 2012, Japan[535] | Prospective cohort study, Participants in this study worked in and around Nagoya City and had annual health check-ups between December 1997 and February 2006,<br>5 y | Women and men,<br>30-69 y | 5856,<br>2068 | HbA1c level, measured in a fasting venous blood sample | Diabetes: no (HbA1c < 6,5%),<br>Diabetes: yes (HbA1c ≥ 6,5%) | Periodontal disease, The PPD around 10 index teeth were examined to obtain the CPI | 1.17 (1.01; 1.36) | Age, gender, smoking status, BMI |
| Lee et al., 2018, South Korea[536] | Prospective cohort study, National Health Insurance Service-Elderly Cohort (NHIS-EC),<br>11 y | Women and men,<br>≥ 60 y | 149785,<br>N.A. | Type 1 and 2 diabetes,<br>ICD-10 codes: E101, E105, E109, E111, E115, E119, E131, E135, E139, E141, E145, E149 | Diabetes yes/no | Periodontal disease, based on the guidelines of the Centers for Disease Control and American Academy of Periodontology using dental history questionnaires, clinical signs, and an oral examination and radiographic evaluation: diagnosis of PD by ICD-10 ([K052],[K053],[K054], [K055], [K056]) and who received periodontal treatment | 1.18 (1.17; 1.20) | Age, gender, household income, insurance status, residence area, health status and other chronic diseases |
| Lee, et al., 2019, | Prospective cohort study, National Health | Women and men, | 78768,<br>15170 | Mainly type diabetes 2, ICD-9 | Mainly type 2 diabetes yes/no | Periodontal Disease, ICD-9 code: | 1.04 (1.01; 1.08) | Age, sex, income, dyslipidemia, |

| | | | | | | | | |
|---|---|---|---|---|---|---|---|---|
| Taiwan[498] | Insurance database Taiwan (NHIRD), Approx. 9 y | >20 y | | codes 250 and 251, but diagnosis after age of 20 years | | periodontitis (523.0 and 523.5) | | hypertension, rheumatoid arthritis, Charlson comorbidity index |
| Sun et al., 2018, Taiwan[537] | Prospective cohort study, National Health Insurance database Taiwan (NHIRD), 14 y | Women and men, <40 y | 21240, 794 | Type 1 diabetes, ICD-9 codes 250.x1 and 250.x3) | Type 1 diabetes yes/no | Periodontal disease, ICD-9 code: periodontitis (523.3 and 523.4) | 1.66 (1.41; 1.96) | Age, gender, urbanization level, comorbidities of CAD, stroke, asthma, COPD, alcohol-related disease and mental disease |
| **Studies of periodontal disease and incident diabetes** | | | | | | | | |
| Alshihayb et al., 2020, USA[532] | Prospective cohort study, Veterans Affairs (VA) Dental Longitudinal Study (DLS) is a subset of the VA Normative Aging Study (NAS), 14 y | Men, 58.9y | 672, 82 | Periodontal disease Clinical periodontal examinations: probing pocket depth at multiple sites per tooth & attachment loss | No/mild vs. moderate/severe periodontal disease | Type 2 diabetes, medical examination interviews based on self-report | 1.33 (0.71; 2.52) | Time-varying age, education, time-varying body mass index, time-varying comprehensive smoking index . |
| Lin et al., 2014, Taiwan[538] | Retrospective cohort study, National Health Insurance Research Database (NHIRD), 5.47 y | Women and men, ≥ 53 y | 44601, 2501 | Periodontal disease, based on claims data (ICD-9-CM codes 523.4 and 523.5); needing subgingival curettage (procedure codes 91006C, 91007C, and 91008C) and periodontal flap procedure (procedure codes 91009C and 91010C) | Periodontitis needing surgical treatment no = comparison / yes = severe Periodontitis | Type 2 diabetes, patients who have been diagnosed with ICD-9-CM codes 250 at least two times and concomitantly received antidiabetic medications | 1.19 (1.10; 1.29) | Age, gender, income, urbanization, hypertension, CAD, hyperlipidemia, obesity |

| | | | | | | | | |
|---|---|---|---|---|---|---|---|---|
| Demmer et al, 2008, USA[539] | Prospective cohort study, National Health and Nutrition Examination Survey (NHANES I) and its Epidemi- ologic Follow-up Study (NHEFS), 17 y | Women and men, 25-74 y | 9296, 817 | Periodontal disease, measurement of gingival inflammation extent, the presence or absence of periodontal pockets, tooth mobility and classified by Periodontal Index (PI) | Category PI0: periodontal healthy (PI= 0); Category PI1: gingivitis (0< PI≤ 0.87); Category PI2: periodontitis (0.88≤ PI≤ 1.60); Category PI3: periodontitis (1.61≤ PI≤ 2.44); Category PI4: periodontitis (2.45≤ PI≤ 5.07); Category PI5: periodontitis (5.08 ≤ PI≤ 8.0); Edentulous | Type 2 diabetes, diabetes listed on the death certificate, self-reported physician diagnosis requiring pharmacological treatment, health care facility stay with a discharge diagnosis of diabetes | PI2: 1.03 (0.65; 1.64) PI3: 2.08 (1.51; 2.87) PI4: 1.71 (1.19; 2.45) PI5: 1.50 (0.99; 2.27) pooled to 1.64 (1.35, 1.99) | Age, gender, race, education, smoking status, BMI, physical activity, hypertension, total cholesterol, subscapular skinfold |
| Ide et al, 2011, Japan[540] | Prospective cohort study, worksite cohort study, 6.5 y | Women and men, 30-59 y | 5848, 287 | Periodontal disease, Community Periodontal Index (CPI) | no pathological pockets (CPI score: 0,1 or 2), moderate periodontitis (CPI score: 3), severe periodontitis (CPI score: 4) | Type 2 diabetes, FPG > 125 mg/dL | CPI score 3: 1.00 (0.77; 1.30) CPI score 4: 1.28 (0.89; 1.86) pooled to 1.09 (0.88, 1.35) | Age, gender, smoking status, BMI, triglyceride level, hypertension, high density lipoprotein level, gamma-glutamyl transpeptidase |

| | | | | | | | | |
|---|---|---|---|---|---|---|---|---|
| Miyawaki et al.,2016, Japan[541] | Prospective cohort study, MY Health Up study, 5 y | Men, 36-55 y | 2469, 133 | Periodontal status, Self-reported periodontal symptoms | Tooth loosening no/yes | Type 2 diabetes, self-reported or met blood test criteria at least once during the follow-up period (FPG level ≥126 mg/dl between 2005 and 2009 and/or HbA1c values ≥ 6,5% in 2008 and 2009) | 1.73 (1.14; .,64) | Age, current smoking habits, BMI, family history of diabetes, hypertension, alcohol heavy consumption (≥ 40 g/day), exercise habits (> 30min, ≥ 2 days/week) |
| Kebede et al.,2012, Germany[542] | Study of Health in Pomerania: SHIP-0 (baseline) and SHIP-2 (follow-up), 11.1 y | Women and men, 56.8 y | 2034, 206 | Periodontal disease, Periodontal measurements assessed at four sites per tooth according to the half-mouth method:<br>1) PPD<br>2) CAL | 1) Mean PPD: analyzed as quartiles and continuously,<br>2) Mean clinical attachment level analyzed in quartiles and continuously | Diabetes, known diabetes (self-reported physician diagnoses or treatment with antidiabetic medication) or HbA1c levels ≥ 6.5% or non-FPG levels ≥11,1 mmol/L | 1) Q2: 1.35 (0.82; 2.32)<br>Q3: 1.22 (0.75; 2.00)<br>Q4: 1.27 (0.78; 2.07)<br><br>2) Q2: 0.61 (0.36; 1.04)<br>Q3: 0.92 (0.56; 1.49)<br>Q4: 0.82 (0.49; 1.37)<br><br>pooled to 0.78 (0.88, 1.05) | Age, gender, highest level of general education, marital status, waist circumference, physical activity, smoking status, dental visits past 12 months, follow-up time |

| | | | | | | | | |
|---|---|---|---|---|---|---|---|---|
| Myllymaki et al.,2018, Finland[543] | Prospective cohort study, MY Health up study, 5 y | Women and men, 36-55 y | 394, 81 | Periodontal condition, Clinical oral examinations by 2 dentists: presence of deepened periodontal pockets (4mm or deeper) | periodontally healthy = No deepened periodontal pockets, PPD of 4-5 mm, PPD of ≥ 6 mm, edentulous, Number of sites with periodontal pockets 4 mm deep or deeper (continuous variable) | Type 2 diabetes, fasting venous plasma glucose ≥7,0 mmol/L and/or 2-hour venous plasma glucose ≥ 11,1 mmol/L after ingestion of 75g of an oral glucose load | PPD of 4-5 mm: 1.32 (0.69; 2.53)<br><br>PPD of ≥ 6 mm: 1.56 (0.58; 1.92)<br><br>pooled to 1.44 (0.93, 2.26) | Gender, risk of diabetes mellitus, physical activity, dietary habits, IGT at baseline, hypertriglyceridemia, low HDL-C, smoking status, BMI, absolute change in BMI during follow up time |
| Winning et al.,2017, Northern Ireland[544] | Prospective cohort study, PRIME study (Prospective Epidemiological Study of Myocardial Infarction), 7.8 y | Men, 58-72 y | 1331, 80 | Periodontal disease, clinical periodontal measurements: 1) PPD 2) CAL | no/mild periodontitis, moderate Periodontitis: ≥2 interproximal sites with CAL ≥4 mm, not on the same tooth or ≥2 interproximal sites with PPD ≥5 mm, not on the same tooth, severe periodontitis: ≥ 2 interproximal sites with CAL ≥6 mm, not on the same tooth, and ≥1 interproximal sites with PPD ≥5 mm | Type 2 diabetes, 1)self-reported: diabetes diagnosis, listing a medication that suggested diabetes management and 2) validated by general medical practioner: FPG ≥ 126mg/dl | Moderate periodontitis: 1.53 (0.86; 2.74)<br><br>Severe periodontitis: 1.85( 1.06; 3.22)<br><br>pooled to 1.69 (1.13, 3.22) | Age, number of teeth, smoking status, toothbrushing frequency, marital status, baseline BMI, baseline CRP, cholesterol, history of CAD, history of hypertension, education years, dental attendance, socio-economic status |

| | | | | | | | | |
|---|---|---|---|---|---|---|---|---|
| Morita et al., 2012, Japan[535] | Prospective cohort study, Participants in this study worked in and around Nagoya City and had annual health check-ups between December 1997 and February 2008, 5 y | Women and men, 30-69 y | 6125, 168 | Periodontal disease, The PPD around 10 index teeth were examined to obtain the CPI | CPI Code 0, CPI Code 3, CPI Code 4 | Type 2 diabetes (HbA1C ≥6,5%), measured in a fasting venous blood sample | CPI score 3: 2.47 (0.78; 7.79)<br><br>CPI score 4: 3.45 (1.08; 11.02)<br><br>pooled to 2.91 (1.29, 6.60) | Age, gender, BMI, alcohol consumption, smoking status |
| Lee et al., 2017, South Korea[545] | Prospective cohort study, National Health Insurance Service-Health Examinee Cohort (NHIS-HEC), 12 y | Women and men, 40-79 y | 354850, 110006 | Periodontal disease, diagnosed clinically in oral checkup examinations in accordance with criteria of the Centers for Disease Control and Prevention/American Academy of Periodontology by a general dentist or a periodontitis (KCD-6 codes K05.2–K05.6, corresponding to ICD-10 codes K05.2–K05.6) | Oral healthy, Periodontal disease | Type 1 and 2 diabetes, diagnosed by physicians or other medical professionals: diabetes mellitus (KCD-6 codes E10–E14, corresponding to ICD-10 codes E10–E14) | 1.16 (1.12; 1.02) | Age, gender, smoking status, income, insurance status, residence area |

**Table S5.** Risk of bias assessment of all included studies in the meta-analysis using the Quality in Prognosis studies (QUIPS) tool.

| | | | **Domains of bias: Low, moderate, high risk of bias, unclear** | | | | |
|---|---|---|---|---|---|---|---|
| **Author, year** | Study participation | Study attrition | Prognostic factor measurement | Outcome measurement | Study confounding | Statistical analysis and reporting | **Overall** |
| **Studies of diabetes mellitus and incident periodontal disease** | | | | | | | |
| Alshihayb, 2020[532] | Moderate | Low | High | Low | Moderate | Low | High |
| Chiu, 2015[533] | Low | Moderate | Low | Low | Moderate | Low | Low |
| Jimenez, 2012[534] | Low | Low | Moderate | High | Moderate | Low | High |
| Lee, 2018[536] | Moderate | Unclear | Moderate | Low | High | Low | Moderate |
| Lee, 2019[546] | Low | Unclear | Moderate | Moderate | High | Low | Moderate |
| Morita 2012[535] | Low | High | Low | Low | Moderate | Low | Moderate |
| Sun, 2018[537] | Low | Unclear | Moderate | Moderate | High | Low | Moderate |
| **Studies of periodontal disease and incident diabetes mellitus** | | | | | | | |
| Alshihayb, 2020[532] | Moderate | Low | Low | High | Moderate | Low | High |
| Demmer, 2008[539] | Low | Low | Moderate | Moderate | Moderate | Low | Moderate |
| Ide, 2011[540] | Low | Moderate | Low | Low | Moderate | Low | Low |
| Kebede, 2018[542] | Low | High | Low | Low | Moderate | Low | Low |
| Lee, 2017[545] | Low | Low | Low | Low | Moderate | Low | Low |
| Lin, 2014[538] | Low | Unclear | High | Moderate | High | Low | High |
| Miyawaki, 2016[541] | Low | Moderate | High | Low | Moderate | Low | High |
| Morita, 2012[535] | Low | High | Low | Low | Moderate | Low | Moderate |
| Myllymaki, 2018[543] | Moderate | High | Low | Low | Moderate | Low | Moderate |
| Winning, 2017[544] | Low | Low | Low | Low | Moderate | Low | Low |

**Table S6:** Certainty of evidence by applying the GRADE tool

| Certainty assessment | | | | | | | Relative risk (95% CI) | Certainty |
|---|---|---|---|---|---|---|---|---|
| № of studies | Study design | Risk of bias | Inconsistency | Indirectness | Imprecision | Other considerations | | |
| **Periodontal diseases and incidence of diabetes mellitus** | | | | | | | | |
| 10 | observational studies | not serious | serious[a] | serious[b] | not serious | Dose response gradient | **RR 1.26** (1.12 to 1.41) | ⨁⨁⨁◯ MODERATE |
| **Diabetes mellitus and incidence of periodontal diseases** | | | | | | | | |
| 7 | observational studies | not serious | not serious[c] | serious[b] | not serious | none | **RR 1.24** (1.13 to 1.37) | ⨁⨁⨁◯ MODERATE |

[a] RR ranges from 0.78 to 2.91 , and 95% CIs do not overlap; $I^2$: 71%

[b] different assessment methods were used to diagnose the disease (e.g. defined by self-reports, clinical examinations or a combination of both), some cohorts include participants with type 1 and type 2 diabetes, different time of follow-up

[c] $I^2$ high (92%), but RRs in same direction

**Table S7:** Summary relative risks (SRRs) and 95% confidence intervals (95% CIs) of periodontal diseases and incidence of diabetes mellitus by subgroups

| | n of studies | SRRs (95% CIs) | $I^2$ (%) | $P_{within}$ | $P_{between}$ |
|---|---|---|---|---|---|
| All studies | 10 | 1.26 (1.12, 1.41) | 71 | <0.0001 | |
| Sex[a] | | | | | |
| Men | 5 | 1.31 (1.01, 1.70) | 58 | 0.048 | 0.109 |
| Women | 2 | 2.00 (1.58, 2.54) | 0 | 0.974 | |
| Risk of bias | | | | | |
| Low | 4 | 1.09 (0.90, 1.32) | 67 | 0.028 | 0.127 |
| Moderate | 3 | 1.66 (1.35, 2.03) | 10 | 0.331 | |
| High | 3 | 1.30 (1.04, 1.63) | 34 | 0.219 | |
| Type of diabetes | | | | | |
| Type 1 diabetes | - | - | - | - | 0.080 |
| Type 2 diabetes | 8 | 1.41 (1.19, 1.67) | 63 | 0.009 | |
| Type 1 + type 2 diabetes | 2 | 0.98 (0.67, 1.44) | 85 | 0.009 | |
| Assessment of diabetes | | | | | |
| Low risk of bias | 7 | 1.24 (1.03, 1.50) | 67 | 0.005 | 0.951 |
| Moderate risk of bias | 2 | 1.38 (1.01, 1.89) | 89 | 0.002 | |
| High risk of bias | 1 | 1.33 (0.71, 2.51) | no meta-analysis n=1 | | |
| Assessment of periodontal diseases | | | | | |
| Low risk of bias | 7 | 1.19 (0.99, 1.42) | 60 | 0.019 | 0.613 |
| Moderate risk of bias | 1 | 1.64 (1.35, 1.99) | no meta-analysis n=1 | | |
| High risk of bias | 2 | 1.35 (0.96, 1.92) | 66 | 0.086 | |
| Geographical location | | | | | |
| Asia | 5 | 1.19 (1.09, 1.30) | 55 | 0.063 | 0.636 |
| USA | 2 | 1.61 (1.34, 1.94) | 0 | 0.535 | |
| Europe | 3 | 1.20 (0.72, 1.99) | 78 | 0.012 | |
| Duration of follow-up | | | | | |
| <10 years | 5 | 1.34 (1.09, 1.65) | 60 | 0.043 | 0.471 |
| ≥10 years | 5 | 1.22 (0.96, 1.55) | 80 | <0.0001 | |

| | | | | | |
|---|---|---|---|---|---|
| Number of cases | | | | | |
| Cases <1000 | 8 | 1.38 (1.08, 1.77) | 73 | <0.0001 | 0.495 |
| Cases ≥1000 | 2 | 1.16 (1.13, 1.20) | 0 | 0.564 | |
| By smoking status[b] | | | | | |
| Never | 3 | 1.39 (1.10, 1.76) | 7 | 0.340 | 0.718 |
| Ever | 2 | 1.28 (1.10, 1.66) | 78 | 0.033 | |
| Adjustment for education | | | | | |
| Yes | 4 | 1.29 (0.83, 1.99) | 83 | <0.0001 | 0.865 |
| No | 6 | 1.20 (1.10, 1.31) | 49 | 0.082 | |
| Adjustment for smoking status | | | | | |
| Yes | 9 | 1.31 (1.10, 1.56) | 74 | <0.0001 | 0.703 |
| No | 1 | 1.19 (1.10, 1.29) | no meta-analysis n=1 | | |
| Adjustment for overweight | | | | | |
| Yes | 9 | 1.32 (1.10, 1.58) | 72 | <0.0001 | 0.624 |
| No | 1 | 1.16 (1.12, 1.20) | no meta-analysis n=1 | | |
| Adjustment for fruit and vegetable intake | | | | | |
| Yes | - | - | - | - | - |
| No | 10 | 1.26 (1.12, 1.41) | 71 | <0.0001 | |
| Adjustment for alcohol intake | | | | | |
| Yes | 2 | 1.98 (1.27, 3.11) | 19 | 0.267 | 0.090 |
| No | 8 | 1.21 (1.09, 1.35) | 70 | 0.002 | |
| Adjustment for physical activity | | | | | |
| Yes | 4 | 1.33 (0.89, 1.97) | 84 | <0.0001 | 0.921 |
| No | 6 | 1.18 (1.10, 1.27) | 35 | 0.117 | |
| Adjustment for number of missing teeth | | | | | |
| Yes | 1 | 1.69 (1.00, 2.85) | no meta-analysis n=1 | | 0.488 |
| No | 9 | 1.24 (1.10, 1.39) | 76 | <0.0001 | |

$P_{within}$, P for heterogeneity within each subgroup; $P_{between}$, P for heterogeneity between subgroups with meta-regression

[a] information available from 5 studies [532, 539-541, 544]

[b] information available from 3 studies [539, 540, 543]

**Table S8:** Summary relative risks (RRs) and 95% confidence intervals (95% CIs) of diabetes mellitus and incidence of periodontal diseases by subgroups

| | n of studies | SRRs (95% CIs) | $I^2$ (%) | $P_{within}$ | $P_{between}$ |
|---|---|---|---|---|---|
| All studies | 7 | 1.24 (1.13, 1.37) | 92 | <0.0001 | |
| Sex[a] | | | | | |
| Men | 3 | 1.35 (1.21, 1.51) | 0 | 0.411 | 0.447 |
| Women | 1 | 1.57 (1.25, 1.98) | no meta-analysis n=1 | | |
| Risk of bias | | | | | |
| Low | 1 | 1.95 (1.22, 3.12) | no meta-analysis n=1 | | 0.404 |
| Moderate | 4 | 1.21 (1.08, 1.34) | 95 | <0.0001 | |
| High | 2 | 1.29 (1,14; 1.47) | 0 | 0.935 | |
| Type of diabetes | | | | | |
| Type 1 diabetes | 1 | 1.66 (1.41, 1.96) | no meta-analysis n=1 | | 0.306 |
| Type 2 diabetes | 4 | 1.25 (1.01, 1.55) | 82 | 0.001 | |
| Type 1 + type 2 diabetes | 2 | 1.18 (1.17, 1.19) | no meta-analysis n=1 | | |
| Assessment of diabetes | | | | | |
| Low risk of bias | 2 | 1.44 (0.88, 2.34) | 76 | 0.043 | 0.917 |
| Moderate risk of bias | 4 | 1.23 (1.11, 1.37) | 96 | <0.0001 | |
| High risk of bias | 1 | 1.32 (0.77, 2.26) | no meta-analysis n=1 | | |
| Assessment of periodontal diseases | | | | | |
| Low risk of bias | 3 | 1.36 (1.00, 1.86) | 56 | 0.124 | 0.911 |
| Moderate risk of bias | 3 | 1.22 (1.08, 1.38) | 97 | <0.0001 | |
| High risk of bias | 1 | 1.29 (1.13, 1.47) | no meta-analysis n=1 | | |
| Geographical location | | | | | |
| Asia | 5 | 1.23 (1.11, 1.37) | 94 | <0.0001 | 0.957 |
| USA | 2 | 1.29 (1.13, 1.47) | 0 | 0.94 | |
| Duration of follow-up | | | | | |
| <10 years | 3 | 1.18 (0.97, 1.43) | 78 | 0.011 | 0.484 |
| ≥10 years | 4 | 1.34 (1.13, 1.58) | 84 | <0.0001 | |
| Number of cases[b] | | | | | 0.201 |

| | | | | | |
|---|---|---|---|---|---|
| Cases <1000 | 2 | 1.63 (1.39, 1.91) | 0 | 0.425 | |
| Cases ≥1000 | 4 | 1.21 (1.03, 1.42) | 83 | <0.0001 | |
| By smoking status[c] | | | | | |
| Never | 1 | 1.32 (1.06, 1.64) | no meta-analysis n=1 | | - |
| Ever | 1 | 1.30 (1.14, 1.49) | no meta-analysis n=1 | | |
| Adjustment for education | | | | | |
| Yes | 2 | 1.64 (1.12, 2.40) | 13 | 0.285 | 0.272 |
| No | 5 | 1.22 (1.11, 1.34) | 94 | <0.0001 | |
| Adjustment for smoking status | | | | | |
| Yes | 4 | 1.28 (1.12, 1.47) | 32 | 0.211 | 0.706 |
| No | 3 | 1.22 (1.08, 1.38) | 97 | <0.0001 | |
| Adjustment for overweight | | | | | |
| Yes | 3 | 1.24 (1.12, 1.36) | 0 | 0.611 | 0.746 |
| No | 4 | 1.25 (1.11, 1.42) | 96 | <0.0001 | |
| Adjustment for fruit and vegetable intake | | | | | |
| Yes | 2 | 1.49 (1.01, 2.18) | 64 | 0.098 | 0.417 |
| No | 5 | 1.21 (1.09, 1.34) | 94 | <0.0001 | |
| Adjustment for alcohol intake | | | | | |
| Yes | 2 | 1.49 (1.01, 2.18) | 64 | 0.098 | 0.417 |
| No | 5 | 1.21 (1.09, 1.34) | 94 | <0.0001 | |
| Adjustment for physical activity | | | | | |
| Yes | 2 | 1.49 (1.01, 2.18) | 64 | 0.098 | 0.417 |
| No | 5 | 1.21 (1.09, 1.34) | 94 | <0.0001 | |
| Adjustment for number of missing teeth at baseline | | | | | |
| Yes | 1 | 1.29 (1.13, 1.47) | no meta-analysis n=1 | | 0.994 |
| No | 6 | 1.24 (1.11, 1.37) | 93 | <0.0001 | |

$P_{within}$, P for heterogeneity within each subgroup; $P_{between}$, P for heterogeneity between subgroups with meta-regression

[a] information available from 3 studies[532], [534], [537]

[b] information available from 6 studies: one study did not provide number of cases in the text [536]

[c] information available from 1 study [534]

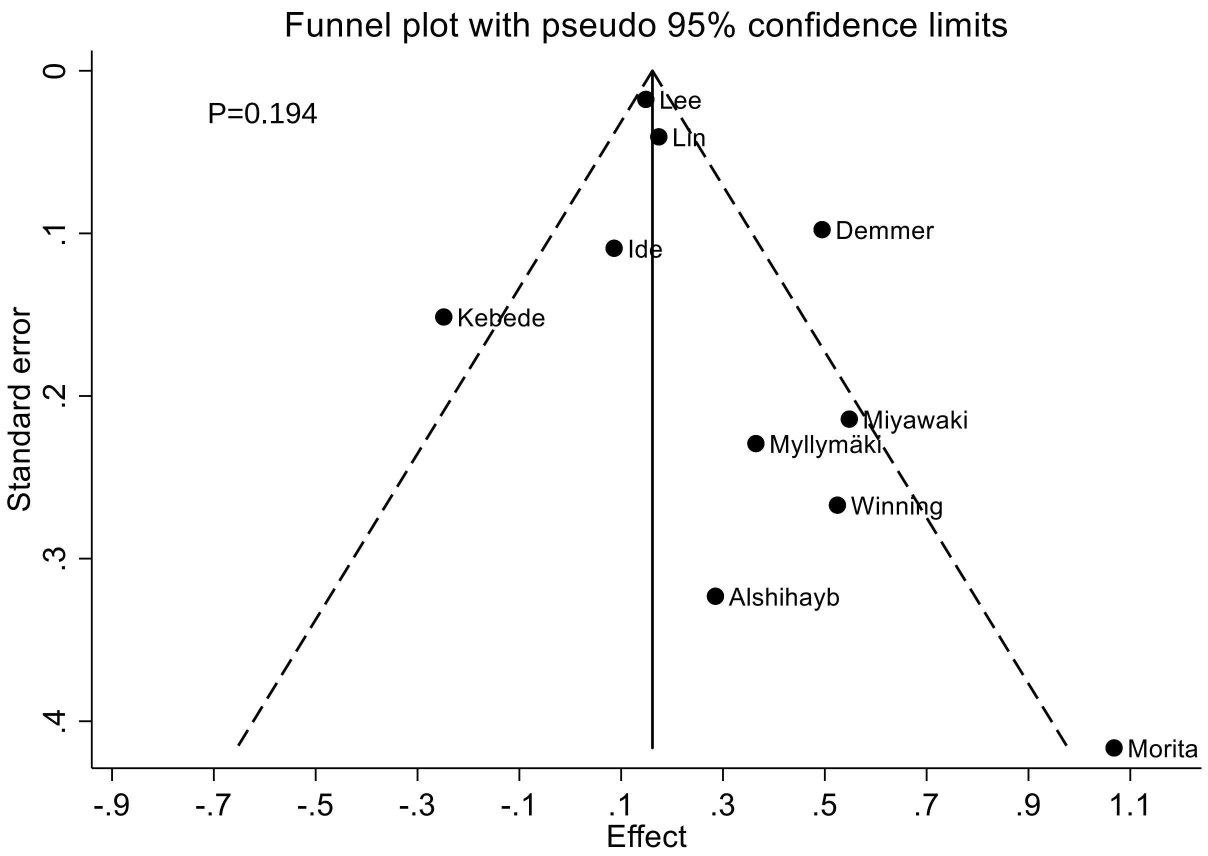


**Figure S2.** Funnel plot for association between periodontal disease and incidence of diabetes mellitus

**References**


1. The relationship between diabetes and oral health among Australian adults. Australian dental journal. 2008;53(1):93-6. Epub 2008/02/29. doi: 10.1111/j.1834-7819.2007.00014.x. PubMed PMID: 18304247.

2. Acharya A, Cheng B, Koralkar R, Olson B, Lamster IB, Kunzel C, et al. Screening for Diabetes Risk Using Integrated Dental and Medical Electronic Health Record Data. JDR clinical and translational research. 2018;3(2):188-94. Epub 2018/03/24. doi: 10.1177/2380084418759496. PubMed PMID: 29568804; PubMed Central PMCID: PMCPMC5858652.

3. Alasqah M, Mokeem S, Alrahlah A, Al-Hamoudi N, Abduljabbar T, Akram Z, et al. Periodontal parameters in prediabetes, type 2 diabetes mellitus, and non-diabetic patients. Brazilian oral research. 2018;32:e81. Epub 2018/08/09. doi: 10.1590/1807-3107bor-2018.vol32.0081. PubMed PMID: 30088554.

4. Albrecht M, Banoczy J, Tamas G, Jr. Dental and oral symptoms of diabetes mellitus. Community dentistry and oral epidemiology. 1988;16(6):378-80. Epub 1988/12/01. PubMed PMID: 3203498.

5. Almas K, Al-Qahtani M, Al-Yami M, Khan N. The relationship between periodontal disease and blood glucose level among type II diabetic patients. The journal of contemporary dental practice. 2001;2(4):18-25. Epub 2002/08/09. PubMed PMID: 12167917.

6. Almeida Abdo J, Cirano FR, Casati MZ, Ribeiro FV, Giampaoli V, Casarin RCV, et al. Influence of dyslipidemia and diabetes mellitus on chronic periodontal disease. Journal of periodontology. 2013;84(10):1401-8. Epub 2012/11/10. doi: 10.1902/jop.2012.120366. PubMed PMID: 23136946.

7. Altamash M, Arledal S, Klinge B, Engstrom PE. Pre-diabetes and diabetes: Medical risk factors and periodontal conditions. Acta odontologica Scandinavica. 2013;71(6):1625-31. Epub 2013/04/17. doi: 10.3109/00016357.2013.788207. PubMed PMID: 23586625.

8. Amaliya A, Laine ML, Delanghe JR, Loos BG, Van Wijk AJ, Van der Velden U. Java project on periodontal diseases: periodontal bone loss in relation to environmental and systemic conditions. Journal of clinical periodontology. 2015;42(4):325-32. Epub 2015/02/17. doi: 10.1111/jcpe.12381. PubMed PMID: 25683157.

9. Aren G, Sepet E, Ozdemir D, Dinccag N, Guvener B, Firatli E. Periodontal health, salivary status, and metabolic control in children with type 1 diabetes mellitus. Journal of periodontology. 2003;74(12):1789-95. Epub 2004/02/21. doi: 10.1902/jop.2003.74.12.1789. PubMed PMID: 14974821.

10. Arrieta-Blanco JJ, Bartolome-Villar B, Jimenez-Martinez E, Saavedra-Vallejo P, Arrieta-Blanco FJ. Dental problems in patients with diabetes mellitus (II): gingival index and periodontal disease. Medicina oral : organo oficial de la Sociedad Espanola de Medicina Oral y de la Academia Iberoamericana de Patologia y Medicina Bucal. 2003;8(4):233-47. Epub 2003/08/26. PubMed PMID: 12937385.

11. Awartani F. Evaluation of the relationship between type 2 diabetes and periodontal disease. Odonto-stomatologie tropicale = Tropical dental journal. 2009;32(128):33-9. Epub 2010/07/10. PubMed PMID: 20614697.

12. Bacic M, Plancak D, Granic M. CPITN assessment of periodontal disease in diabetic patients. Journal of periodontology. 1988;59(12):816-22. Epub 1988/12/01. doi: 10.1902/jop.1988.59.12.816. PubMed PMID: 3225728.

13. Bahru Y, Abdu SS. A study of dental problems in diabetic patients. Ethiopian medical journal. 1992;30(2):95-103. Epub 1992/04/01. PubMed PMID: 1606949.

14. Bajaj S, Prasad S, Gupta A, Singh VB. Oral manifestations in type-2 diabetes and related complications. Indian journal of endocrinology and metabolism. 2012;16(5):777-9. Epub 2012/10/23. doi: 10.4103/2230-8210.100673. PubMed PMID: 23087863; PubMed Central PMCID: PMCPMC3475903.

15. Balkaran R, Naidu R, Teelucksingh S, Seemungal T, Pinto Pereira L, Prayman E, et al. A preliminary investigation of periodontal disease and diabetes in Trinidad. The West Indian medical journal. 2011;60(1):86-90. Epub 2011/08/04. PubMed PMID: 21809719.

16. Barnett ML, Baker RL, Yancey JM, MacMillan DR, Kotoyan M. Absence of periodontitis in a population of insulin-dependent diabetes mellitus (IDDM) patients. Journal of periodontology. 1984;55(7):402-5. Epub 1984/07/01. doi: 10.1902/jop.1984.55.7.402. PubMed PMID: 6589390.

17. Bharateesh J, Ahmed M, Kokila G. Diabetes and Oral Health: A Case-control Study. International journal of preventive medicine. 2012;3(11):806-9. Epub 2012/11/29. PubMed PMID: 23189233; PubMed Central PMCID: PMCPMC3506093.

18. Bokhari SA, Suhail AM, Malik AR, Imran MF. Periodontal disease status and associated risk factors in patients attending a Dental Teaching Hospital in Rawalpindi, Pakistan. Journal of Indian Society of Periodontology. 2015;19(6):678-82. Epub 2016/03/05. doi: 10.4103/0972-124x.156882. PubMed PMID: 26941520; PubMed Central PMCID: PMCPMC4753714.

19. Bridges RB, Anderson JW, Saxe SR, Gregory K, Bridges SR. Periodontal status of diabetic and non-diabetic men: effects of smoking, glycemic control, and socioeconomic factors. Journal of periodontology. 1996;67(11):1185-92. Epub 1996/11/01. doi: 10.1902/jop.1996.67.11.1185. PubMed PMID: 8959568.

20. Brothwell D, Ghiabi E. Periodontal health status of the Sandy Bay First Nation in Manitoba, Canada. International journal of circumpolar health. 2009;68(1):23-33. Epub 2009/04/01. PubMed PMID: 19331239.

21. Buysschaert M, Tshongo Muhindo C, Alexopoulou O, Rahelic D, Reychler H, Preumont V. Oral hygiene behaviours and tooth-loss assessment in patients with diabetes: A report from a diabetology centre in Belgium. Diabetes & metabolism. 2017;43(3):272-4. Epub 2016/10/04. doi: 10.1016/j.diabet.2016.08.003. PubMed PMID: 27692742.

22. Campbell MJ. Epidemiology of periodontal disease in the diabetic and the non-diabetic. Australian dental journal. 1972;17(4):274-8. Epub 1972/08/01. PubMed PMID: 4510020.

23. Cherry-Peppers G, Ship JA. Oral health in patients with type II diabetes and impaired glucose tolerance. Diabetes care. 1993;16(4):638-41. Epub 1993/04/01. PubMed PMID: 8462394.

24. Chrysanthakopoulos NA, Chrysanthakopoulos PA. Association of periodontal disease with self-reported systemic disorders in Greece. Oral health & preventive dentistry.

2013;11(3):251-60. Epub 2013/07/24. doi: 10.3290/j.ohpd.a30167. PubMed PMID: 23878836.

25. Cohen DW, Friedman LA, Shapiro J, Kyle GC, Franklin S. Diabetes mellitus and periodontal disease: two-year longitudinal observations. I. Journal of periodontology. 1970;41(12):709-12. Epub 1970/12/01. doi: 10.1902/jop.1970.41.12.709. PubMed PMID: 5275715.

26. Collin HL, Uusitupa M, Niskanen L, Kontturi-Narhi V, Markkanen H, Koivisto AM, et al. Periodontal findings in elderly patients with non-insulin dependent diabetes mellitus. Journal of periodontology. 1998;69(9):962-6. Epub 1998/10/17. doi: 10.1902/jop.1998.69.9.962. PubMed PMID: 9776023.

27. Commisso L, Monami M, Mannucci E. Periodontal disease and oral hygiene habits in a type 2 diabetic population. International journal of dental hygiene. 2011;9(1):68-73. Epub 2011/01/14. doi: 10.1111/j.1601-5037.2009.00439.x. PubMed PMID: 21226853.

28. Costa KL, Taboza ZA, Angelino GB, Silveira VR, Montenegro R, Jr., Haas AN, et al. Influence of Periodontal Disease on Changes of Glycated Hemoglobin Levels in Patients With Type 2 Diabetes Mellitus: A Retrospective Cohort Study. Journal of periodontology. 2017;88(1):17-25. Epub 2016/08/27. doi: 10.1902/jop.2016.160140. PubMed PMID: 27562220.

29. Cutler CW, Machen RL, Jotwani R, Iacopino AM. Heightened gingival inflammation and attachment loss in type 2 diabetics with hyperlipidemia. Journal of periodontology. 1999;70(11):1313-21. Epub 1999/12/10. doi: 10.1902/jop.1999.70.11.1313. PubMed PMID: 10588494.

30. Das M, Upadhyaya V, Ramachandra SS, Jithendra KD. Periodontal treatment needs in diabetic and non-diabetic individuals: a case-control study. Indian journal of dental research : official publication of Indian Society for Dental Research. 2011;22(2):291-4. Epub 2011/09/06. doi: 10.4103/0970-9290.84307. PubMed PMID: 21891902.

31. de Pommereau V, Dargent-Pare C, Robert JJ, Brion M. Periodontal status in insulin-dependent diabetic adolescents. Journal of clinical periodontology. 1992;19(9 Pt 1):628-32. Epub 1992/10/01. PubMed PMID: 1430290.

32. Deguchi M, Mau M, Davis J, Niederman R. Preventable Tooth Loss in Hawai'i: The Role of Socioeconomic Status, Diabetes, and Dental Visits. Preventing chronic disease. 2017;14:E115. Epub 2017/11/18. doi: 10.5888/pcd14.170214. PubMed PMID: 29144892; PubMed Central PMCID: PMCPMC5695642.

33. Finestone AJ, Boorujy SR. Diabetes mellitus and periodontal disease. Diabetes. 1967;16(5):336-40. Epub 1967/05/01. PubMed PMID: 6022561.

34. Firatli E. The relationship between clinical periodontal status and insulin-dependent diabetes mellitus. Results after 5 years. Journal of periodontology. 1997;68(2):136-40. Epub 1997/02/01. doi: 10.1902/jop.1997.68.2.136. PubMed PMID: 9058330.

35. Fontana G, Lapolla A, Sanzari M, Piva E, Mussap M, De Toni S, et al. An immunological evaluation of type II diabetic patients with periodontal disease. Journal of diabetes and its complications. 1999;13(1):23-30. Epub 1999/05/08. PubMed PMID: 10232706.

36. Furukawa T, Wakai K, Yamanouchi K, Oshida Y, Miyao M, Watanabe T, et al. Associations of periodontal damage and tooth loss with atherogenic factors among patients

with type 2 diabetes mellitus. Internal medicine (Tokyo, Japan). 2007;46(17):1359-64. Epub 2007/09/11. PubMed PMID: 17827833.

37. Geisinger ML, Morris AB, Kaur M, Hardy SL, Abou Arraj RV, Geurs NC, et al. Glycemic control among patients with physician-managed type 2 diabetes. General dentistry. 2018;66(5):52-5. Epub 2018/09/07. PubMed PMID: 30188857.

38. Gupta N, Gupta ND, Garg S, Goyal L, Gupta A, Khan S, et al. The effect of type 2 diabetes mellitus and smoking on periodontal parameters and salivary matrix metalloproteinase-8 levels. Journal of oral science. 2016;58(1):1-6. Epub 2016/03/30. doi: 10.2334/josnusd.58.1. PubMed PMID: 27021533.

39. Gupta N, Gupta ND, Gupta A, Goyal L, Garg S. The influence of type 2 diabetes mellitus on salivary matrix metalloproteinase-8 levels and periodontal parameters: A study in an Indian population. European journal of dentistry. 2015;9(3):319-23. Epub 2015/10/03. doi: 10.4103/1305-7456.163222. PubMed PMID: 26430357; PubMed Central PMCID: PMCPMC4569980.

40. Haseeb M, Khawaja KI, Ataullah K, Munir MB, Fatima A. Periodontal disease in type 2 diabetes mellitus. Journal of the College of Physicians and Surgeons--Pakistan : JCPSP. 2012;22(8):514-8. Epub 2012/08/08. doi: 08.2012/jcpsp.514518. PubMed PMID: 22868018.

41. Hayashida H, Kawasaki K, Yoshimura A, Kitamura M, Furugen R, Nakazato M, et al. Relationship between periodontal status and HbA1c in nondiabetics. Journal of public health dentistry. 2009;69(3):204-6. Epub 2009/06/03. doi: 10.1111/j.1752-7325.2009.00122.x. PubMed PMID: 19486462.

42. Hayden P, Buckley LA. Diabetes mellitus and periodontal disease in an Irish population. Journal of periodontal research. 1989;24(5):298-302. Epub 1989/09/01. PubMed PMID: 2533253.

43. Hove KA, Stallard RE. Diabetes and the periodontal patient. Journal of periodontology. 1970;41(12):713-8. Epub 1970/12/01. doi: 10.1902/jop.1970.41.12.713. PubMed PMID: 5275716.

44. Hugoson A, Thorstensson H, Falk H, Kuylenstierna J. Periodontal conditions in insulin-dependent diabetics. Journal of clinical periodontology. 1989;16(4):215-23. Epub 1989/04/01. PubMed PMID: 2785536.

45. Izuora K, Ezeanolue E, Schlauch K, Neubauer M, Gewelber C, Umpierrez G. Impact of periodontal disease on outcomes in diabetes. Contemporary clinical trials. 2015;41:93-9. Epub 2015/01/28. doi: 10.1016/j.cct.2015.01.011. PubMed PMID: 25623292; PubMed Central PMCID: PMCPMC4380752.

46. Jansson H, Lindholm E, Lindh C, Groop L, Bratthall G. Type 2 diabetes and risk for periodontal disease: a role for dental health awareness. Journal of clinical periodontology. 2006;33(6):408-14. Epub 2006/05/09. doi: 10.1111/j.1600-051X.2006.00929.x. PubMed PMID: 16677329.

47. Javed F, Al-Askar M, Al-Rasheed A, Babay N, Galindo-Moreno P, Al-Hezaimi K. Comparison of self-perceived oral health, periodontal inflammatory conditions and socioeconomic status in individuals with and without prediabetes. The American journal of the medical sciences. 2012;344(2):100-4. Epub 2011/12/14. doi: 10.1097/MAJ.0b013e31823650a7. PubMed PMID: 22157389.

48. Javed F, Al-Kheraif AA, Salazar-Lazo K, Yanez-Fontenla V, Aldosary KM, Alshehri M, et al. Periodontal Inflammatory Conditions Among Smokers and Never-Smokers With and Without Type 2 Diabetes Mellitus. Journal of periodontology. 2015;86(7):839-46. Epub 2015/04/17. doi: 10.1902/jop.2015.150120. PubMed PMID: 25879874.

49. Javed F, Nasstrom K, Benchimol D, Altamash M, Klinge B, Engstrom PE. Comparison of periodontal and socioeconomic status between subjects with type 2 diabetes mellitus and non-diabetic controls. Journal of periodontology. 2007;78(11):2112-9. Epub 2007/11/01. doi: 10.1902/jop.2007.070186. PubMed PMID: 17970677.

50. Kakade SP, Shetiya SH, Kakodkar P, Shirahatti RV, Agrawal D. Periodontal status of type I diabetics compared to non-diabetic participants: a preliminary study. The Ceylon medical journal. 2014;59(1):19-20. Epub 2014/04/01. doi: 10.4038/cmj.v59i1.5259. PubMed PMID: 24682193.

51. Kalsi DS, Chopra J, Sood A. Association of lipid profile test values, type-2 diabetes mellitus, and periodontitis. Indian journal of dentistry. 2015;6(2):81-4. Epub 2015/06/23. doi: 10.4103/0975-962x.157270. PubMed PMID: 26097337; PubMed Central PMCID: PMCPMC4455160.

52. Karikoski A, Ilanne-Parikka P, Murtomaa H. Oral self-care and periodontal health indicators among adults with diabetes in Finland. Acta odontologica Scandinavica. 2001;59(6):390-5. Epub 2002/02/08. PubMed PMID: 11831490.

53. Karthik SJ, Anoop S, Kumar RS, Rani MVU. Predictors for Gingival Index in Middle-Aged Asian Indians with Type 2 Diabetes from South India: A Cross-Sectional Observational Study. TheScientificWorldJournal. 2018;2018:9081572. Epub 2018/02/27. doi: 10.1155/2018/9081572. PubMed PMID: 29479293; PubMed Central PMCID: PMCPMC5816844.

54. Katagiri S, Nitta H, Nagasawa T, Izumi Y, Kanazawa M, Matsuo A, et al. Effect of glycemic control on periodontitis in type 2 diabetic patients with periodontal disease. Journal of diabetes investigation. 2013;4(3):320-5. Epub 2013/09/03. doi: 10.1111/jdi.12026. PubMed PMID: 23997922; PubMed Central PMCID: PMCPMC3752968.

55. Kathiresan TS, Masthan KMK, Sarangarajan R, Babu NA, Kumar P. A Study of Diabetes Associated Oral Manifestations. Journal of pharmacy & bioallied sciences. 2017;9(Suppl 1):S211-s6. Epub 2017/12/30. doi: 10.4103/jpbs.JPBS_157_17. PubMed PMID: 29284966; PubMed Central PMCID: PMCPMC5731015.

56. Katz J, Chaushu G, Sgan-Cohen HD. Relationship of blood glucose level to community periodontal index of treatment needs and body mass index in a permanent Israeli military population. Journal of periodontology. 2000;71(10):1521-7. Epub 2000/11/04. doi: 10.1902/jop.2000.71.10.1521. PubMed PMID: 11063383.

57. Kawamura M, Fukuda S, Kawabata K, Iwamoto Y. Comparison of health behaviour and oral/medical conditions in non-insulin-dependent (type II) diabetics and non-diabetics. Australian dental journal. 1998;43(5):315-20. Epub 1998/12/16. PubMed PMID: 9848981.

58. Khader YS, Albashaireh ZS, Hammad MM. Periodontal status of type 2 diabetics compared with nondiabetics in north Jordan. Eastern Mediterranean health journal = La revue de sante de la Mediterranee orientale = al-Majallah al-sihhiyah li-sharq al-mutawassit. 2008;14(3):654-61. Epub 2008/08/30. PubMed PMID: 18720630.

59. Khader YS, Rice JC, Lefante JJ. Factors associated with periodontal diseases in a dental teaching clinic population in northern Jordan. Journal of periodontology.

2003;74(11):1610-7. Epub 2003/12/20. doi: 10.1902/jop.2003.74.11.1610. PubMed PMID: 14682657.

60. Khanuja PK, Narula SC, Rajput R, Sharma RK, Tewari S. Association of periodontal disease with glycemic control in patients with type 2 diabetes in Indian population. Frontiers of medicine. 2017;11(1):110-9. Epub 2017/01/05. doi: 10.1007/s11684-016-0484-5. PubMed PMID: 28050765.

61. Kiedrowicz M, Dembowska E, Banach J, Safranow K, Pynka S. A comparison of the periodontal status in patients with type 2 diabetes based on glycated haemoglobin levels and other risk factors. Advances in medical sciences. 2015;60(1):156-61. Epub 2015/02/28. doi: 10.1016/j.advms.2015.01.007. PubMed PMID: 25723568.

62. Kim EK, Lee SG, Choi YH, Won KC, Moon JS, Merchant AT, et al. Association between diabetes-related factors and clinical periodontal parameters in type-2 diabetes mellitus. BMC oral health. 2013;13:64. Epub 2013/11/08. doi: 10.1186/1472-6831-13-64. PubMed PMID: 24195646; PubMed Central PMCID: PMCPMC3829373.

63. Kogawa EM, Grisi DC, Falcao DP, Amorim IA, Rezende TM, da Silva IC, et al. Impact of glycemic control on oral health status in type 2 diabetes individuals and its association with salivary and plasma levels of chromogranin A. Archives of oral biology. 2016;62:10-9. Epub 2015/11/26. doi: 10.1016/j.archoralbio.2015.11.005. PubMed PMID: 26605682.

64. Lagervall M, Jansson L. Relationship between tooth loss/probing depth and systemic disorders in periodontal patients. Swedish dental journal. 2007;31(1):1-9. Epub 2007/05/19. PubMed PMID: 17508705.

65. Lagervall M, Jansson L, Bergstrom J. Systemic disorders in patients with periodontal disease. Journal of clinical periodontology. 2003;30(4):293-9. Epub 2003/04/16. PubMed PMID: 12694426.

66. Lalla E, Park DB, Papapanou PN, Lamster IB. Oral disease burden in Northern Manhattan patients with diabetes mellitus. American journal of public health. 2004;94(5):755-8. Epub 2004/05/01. PubMed PMID: 15117696; PubMed Central PMCID: PMCPMC1448333.

67. Lamster IB, Cheng B, Burkett S, Lalla E. Periodontal findings in individuals with newly identified pre-diabetes or diabetes mellitus. Journal of clinical periodontology. 2014;41(11):1055-60. Epub 2014/09/10. doi: 10.1111/jcpe.12307. PubMed PMID: 25195497.

68. Lee HK, Lee KD, Merchant AT, Lee SK, Song KB, Lee SG, et al. More missing teeth are associated with poorer general health in the rural Korean elderly. Archives of gerontology and geriatrics. 2010;50(1):30-3. Epub 2009/02/24. doi: 10.1016/j.archger.2009.01.005. PubMed PMID: 19230988.

69. Lim LP, Tay FB, Sum CF, Thai AC. Relationship between markers of metabolic control and inflammation on severity of periodontal disease in patients with diabetes mellitus. Journal of clinical periodontology. 2007;34(2):118-23. Epub 2007/02/21. doi: 10.1111/j.1600-051X.2006.01032.x. PubMed PMID: 17309586.

70. Loe H. Periodontal disease. The sixth complication of diabetes mellitus. Diabetes care. 1993;16(1):329-34. Epub 1993/01/01. PubMed PMID: 8422804.

71. Losche W, Karapetow F, Pohl A, Pohl C, Kocher T. Plasma lipid and blood glucose levels in patients with destructive periodontal disease. Journal of clinical periodontology. 2000;27(8):537-41. Epub 2000/08/26. PubMed PMID: 10959778.

72. Lu HK, Yang PC. Cross-sectional analysis of different variables of patients with non-insulin dependent diabetes and their periodontal status. The International journal of periodontics & restorative dentistry. 2004;24(1):71-9. Epub 2004/02/27. PubMed PMID: 14984148.

73. Mansour AA, Abd-Al-Sada N. Periodontal disease among diabetics in Iraq. MedGenMed : Medscape general medicine. 2005;7(3):2. Epub 2005/12/22. PubMed PMID: 16369228; PubMed Central PMCID: PMCPMC1681616.

74. Mattout C, Bourgeois D, Bouchard P. Type 2 diabetes and periodontal indicators: epidemiology in France 2002-2003. Journal of periodontal research. 2006;41(4):253-8. Epub 2006/07/11. doi: 10.1111/j.1600-0765.2006.00862.x. PubMed PMID: 16827717.

75. Matu NK, Stephen L, Lalloo R. Prevalence and severity of periodontal disease: type 2 diabetics versus non-diabetics. SADJ : journal of the South African Dental Association = tydskrif van die Suid-Afrikaanse Tandheelkundige Vereniging. 2009;64(2):64, 6-8. Epub 2009/06/13. PubMed PMID: 19517857.

76. Meenawat A, Punn K, Srivastava V, Meenawat AS, Dolas RS, Govila V. Periodontal disease and type I diabetes mellitus: Associations with glycemic control and complications. Journal of Indian Society of Periodontology. 2013;17(5):597-600. Epub 2013/11/01. doi: 10.4103/0972-124x.119286. PubMed PMID: 24174752; PubMed Central PMCID: PMCPMC3808013.

77. Molloy J, Wolff LF, Lopez-Guzman A, Hodges JS. The association of periodontal disease parameters with systemic medical conditions and tobacco use. Journal of clinical periodontology. 2004;31(8):625-32. Epub 2004/07/20. doi: 10.1111/j.1600-051X.2004.00539.x. PubMed PMID: 15257739.

78. Morton AA, Williams RW, Watts TL. Initial study of periodontal status in non-insulin-dependent diabetics in Mauritius. Journal of dentistry. 1995;23(6):343-5. Epub 1995/12/01. PubMed PMID: 8530724.

79. Mourao LC, Cataldo Dde M, Moutinho H, Canabarro A. Impact of chronic periodontitis on quality-of-life and on the level of blood metabolic markers. Journal of Indian Society of Periodontology. 2015;19(2):155-8. Epub 2015/05/28. doi: 10.4103/0972-124x.149935. PubMed PMID: 26015664; PubMed Central PMCID: PMCPMC4439623.

80. Natto ZS, Aladmawy M, Alasqah M, Papas A. Factors contributing to tooth loss among the elderly: A cross sectional study. Singapore dental journal. 2014;35:17-22. Epub 2014/12/17. doi: 10.1016/j.sdj.2014.11.002. PubMed PMID: 25496581.

81. Nibali L, D'Aiuto F, Griffiths G, Patel K, Suvan J, Tonetti MS. Severe periodontitis is associated with systemic inflammation and a dysmetabolic status: a case-control study. Journal of clinical periodontology. 2007;34(11):931-7. Epub 2007/09/20. doi: 10.1111/j.1600-051X.2007.01133.x. PubMed PMID: 17877746.

82. Nichols C, Laster LL, Bodak-Gyovai LZ. Diabetes mellitus and periodontal disease. Journal of periodontology. 1978;49(2):85-8. Epub 1978/02/01. doi: 10.1902/jop.1978.49.2.85. PubMed PMID: 276596.

83. Novaes AB, Jr., Gutierrez FG, Novaes AB. Periodontal disease progression in type II non-insulin-dependent diabetes mellitus patients (NIDDM). Part I--Probing pocket depth and clinical attachment. Brazilian dental journal. 1996;7(2):65-73. Epub 1996/01/01. PubMed PMID: 9206356.

84. Novaes AB, Jr., Silva MA, Batista EL, Jr., dos Anjos BA, Novaes AB, Pereira AL. Manifestations of insulin-dependent diabetes mellitus in the periodontium of young Brazilian patients. A 10-year follow-up study. Journal of periodontology. 1997;68(4):328-34. Epub 1997/04/01. doi: 10.1902/jop.1997.68.4.328. PubMed PMID: 9150037.

85. Novak MJ, Potter RM, Blodgett J, Ebersole JL. Periodontal disease in Hispanic Americans with type 2 diabetes. Journal of periodontology. 2008;79(4):629-36. Epub 2008/04/03. doi: 10.1902/jop.2008.070442. PubMed PMID: 18380555.

86. Ogunbodede EO, Fatusi OA, Akintomide A, Kolawole K, Ajayi A. Oral health status in a population of Nigerian diabetics. The journal of contemporary dental practice. 2005;6(4):75-84. Epub 2005/11/22. PubMed PMID: 16299609.

87. Oliver RC, Brown LJ, Loe H. Periodontal diseases in the United States population. Journal of periodontology. 1998;69(2):269-78. Epub 1998/04/04. doi: 10.1902/jop.1998.69.2.269. PubMed PMID: 9526927.

88. Oliver RC, Tervonen T. Periodontitis and tooth loss: comparing diabetics with the general population. Journal of the American Dental Association (1939). 1993;124(12):71-6. Epub 1993/12/01. PubMed PMID: 8277062.

89. Orbak R, Tezel A, Canakci V, Demir T. The influence of smoking and non-insulin-dependent diabetes mellitus on periodontal disease. The Journal of international medical research. 2002;30(2):116-25. Epub 2002/05/25. doi: 10.1177/147323000203000203. PubMed PMID: 12025518.

90. Peck T, Price C, English P, Gill G. Oral health in rural South African type 2 diabetic patients. Tropical doctor. 2006;36(2):111-2. Epub 2006/04/14. doi: 10.1258/004947506776593477. PubMed PMID: 16611449.

91. Plancak D, Vizner B, Jorgic-Srdjak K, Slaj M. Endocrinological status of patients with periodontal disease. Collegium antropologicum. 1998;22 Suppl:51-5. Epub 1999/02/10. PubMed PMID: 9951140.

92. Poplawska-Kita A, Siewko K, Szpak P, Krol B, Telejko B, Klimiuk PA, et al. Association between type 1 diabetes and periodontal health. Advances in medical sciences. 2014;59(1):126-31. Epub 2014/05/07. doi: 10.1016/j.advms.2014.01.002. PubMed PMID: 24797988.

93. Preferansow E, Sawczuk B, Golebiewska M, Gorska M. Pathologies of the oral cavity in patients with non-controlled diabetes type 1 and type 2--analysis of periodontal status and periodontal treatment needs. Endokrynologia Polska. 2015;66(5):428-33. Epub 2015/10/13. doi: 10.5603/ep.2015.0053. PubMed PMID: 26457498.

94. Preshaw PM, de Silva N, McCracken GI, Fernando DJ, Dalton CF, Steen ND, et al. Compromised periodontal status in an urban Sri Lankan population with type 2 diabetes. Journal of clinical periodontology. 2010;37(2):165-71. Epub 2010/07/27. doi: 10.1111/j.1600-051X.2009.01519.x. PubMed PMID: 20653819.

95. Rajan P, Nera M, Pavalura AK, Medandrao N, Kumar SC. Comparison of glycosylated hemoglobin (HbA1C) levels in patients with chronic periodontitis and healthy controls. Dental research journal. 2013;10(3):389-93. Epub 2013/09/11. PubMed PMID: 24019810; PubMed Central PMCID: PMCPMC3760365.

96. Rajhans NS, Kohad RM, Chaudhari VG, Mhaske NH. A clinical study of the relationship between diabetes mellitus and periodontal disease. Journal of Indian Society of

Periodontology. 2011;15(4):388-92. Epub 2012/03/01. doi: 10.4103/0972-124x.92576. PubMed PMID: 22368365; PubMed Central PMCID: PMCPMC3283938.

97. Rao Deepika PC, Saxena RM. Comparison of glycosylated hemoglobin levels in severe periodontitis patients and healthy controls: a study in an Indian population. Quintessence international (Berlin, Germany : 1985). 2013;44(4):319-25. Epub 2013/03/13. doi: 10.3290/j.qi.a29144. PubMed PMID: 23479572.

98. Renvert S, Persson RE, Persson GR. Tooth loss and periodontitis in older individuals: results from the Swedish National Study on Aging and Care. Journal of periodontology. 2013;84(8):1134-44. Epub 2012/10/24. doi: 10.1902/jop.2012.120378. PubMed PMID: 23088532.

99. Rylander H, Ramberg P, Blohme G, Lindhe J. Prevalence of periodontal disease in young diabetics. Journal of clinical periodontology. 1987;14(1):38-43. Epub 1987/01/01. PubMed PMID: 3468127.

100. Sadzeviciene R, Paipaliene P, Zekonis G, Zilinskas J. The influence of microvascular complications caused by diabetes mellitus on the inflammatory pathology of periodontal tissues. Stomatologija. 2005;7(4):121-4. Epub 2006/02/28. PubMed PMID: 16501314.

101. Safkan-Seppala B, Ainamo J. Periodontal conditions in insulin-dependent diabetes mellitus. Journal of clinical periodontology. 1992;19(1):24-9. Epub 1992/01/01. PubMed PMID: 1732306.

102. Sandberg GE, Sundberg HE, Fjellstrom CA, Wikblad KF. Type 2 diabetes and oral health: a comparison between diabetic and non-diabetic subjects. Diabetes research and clinical practice. 2000;50(1):27-34. Epub 2000/08/11. PubMed PMID: 10936666.

103. Santos VR, Lima JA, Miranda TS, Feres M, Zimmermann GS, Nogueira-Filho Gda R, et al. Relationship between glycemic subsets and generalized chronic periodontitis in type 2 diabetic Brazilian subjects. Archives of oral biology. 2012;57(3):293-9. Epub 2011/09/06. doi: 10.1016/j.archoralbio.2011.08.003. PubMed PMID: 21890108.

104. Sardi JC, Duque C, Camargo GA, Hofling JF, Goncalves RB. Periodontal conditions and prevalence of putative periodontopathogens and Candida spp. in insulin-dependent type 2 diabetic and non-diabetic patients with chronic periodontitis--a pilot study. Archives of oral biology. 2011;56(10):1098-105. Epub 2011/05/03. doi: 10.1016/j.archoralbio.2011.03.017. PubMed PMID: 21529777.

105. Sastrowijoto SH, Hillemans P, van Steenbergen TJ, Abraham-Inpijn L, de Graaff J. Periodontal condition and microbiology of healthy and diseased periodontal pockets in type 1 diabetes mellitus patients. Journal of clinical periodontology. 1989;16(5):316-22. Epub 1989/05/01. PubMed PMID: 2723104.

106. Saxena RM, Deepika PC. Comparison of glycosylated hemoglobin levels in periodontitis patients and healthy controls: a pilot study in Indian population. Indian journal of dental research : official publication of Indian Society for Dental Research. 2012;23(3):368-72. Epub 2012/10/13. doi: 10.4103/0970-9290.102231. PubMed PMID: 23059575.

107. Schjetlein AL, Jorgensen ME, Lauritzen T, Pedersen ML. Periodontal status among patients with diabetes in Nuuk, Greenland. International journal of circumpolar health. 2014;73:26093. Epub 2014/12/17. doi: 10.3402/ijch.v73.26093. PubMed PMID: 25498562; PubMed Central PMCID: PMCPMC4265130.

108. Schmalz G, Schiffers N, Schwabe S, Vasko R, Muller GA, Haak R, et al. Dental and periodontal health, and microbiological and salivary conditions in patients with or without diabetes undergoing haemodialysis. International dental journal. 2017;67(3):186-93. Epub 2017/05/27. doi: 10.1111/idj.12282. PubMed PMID: 28547773.

109. Seethalakshmi C, Reddy RC, Asifa N, Prabhu S. Correlation of Salivary pH, Incidence of Dental Caries and Periodontal Status in Diabetes Mellitus Patients: A Cross-sectional Study. Journal of clinical and diagnostic research : JCDR. 2016;10(3):Zc12-4. Epub 2016/05/03. doi: 10.7860/jcdr/2016/16310.7351. PubMed PMID: 27134992; PubMed Central PMCID: PMCPMC4843377.

110. Seppala B, Ainamo J. A site-by-site follow-up study on the effect of controlled versus poorly controlled insulin-dependent diabetes mellitus. Journal of clinical periodontology. 1994;21(3):161-5. Epub 1994/03/01. PubMed PMID: 8157767.

111. Seppala B, Seppala M, Ainamo J. A longitudinal study on insulin-dependent diabetes mellitus and periodontal disease. Journal of clinical periodontology. 1993;20(3):161-5. Epub 1993/03/01. PubMed PMID: 8450080.

112. Serrano C, Perez C, Rodriguez M. Periodontal conditions in a group of Colombian type 2 diabetic patients with different degrees of metabolic control. Acta odontologica latinoamericana : AOL. 2012;25(1):132-9. Epub 2012/08/30. PubMed PMID: 22928393.

113. Shanon IL, Kilgore WG. Fasting and postprandial serum glucose in relation to periodontal status. Journal of periodontal research. 1968;3(2):129-31. Epub 1968/01/01. PubMed PMID: 4249989.

114. Soory M. Periodontal disease severity and systemic diseases prevalent in a Caribbean catchment area of patients. The West Indian medical journal. 2007;56(2):190-3. Epub 2007/10/04. PubMed PMID: 17910154.

115. Sudiono J. The features of degenerative diseases and their association with the loss of teeth in the elderly of East Jakarta (Indonesia). The Southeast Asian journal of tropical medicine and public health. 2008;39(1):184-9. Epub 2008/06/24. PubMed PMID: 18567460.

116. Takeda M, Ojima M, Yoshioka H, Inaba H, Kogo M, Shizukuishi S, et al. Relationship of serum advanced glycation end products with deterioration of periodontitis in type 2 diabetes patients. Journal of periodontology. 2006;77(1):15-20. Epub 2006/04/04. doi: 10.1902/jop.2006.77.1.15. PubMed PMID: 16579698.

117. Tanwir F, Altamash M, Gustafsson A. Effect of diabetes on periodontal status of a population with poor oral health. Acta odontologica Scandinavica. 2009;67(3):129-33. Epub 2009/04/16. doi: 10.1080/00016350802208406. PubMed PMID: 19367474.

118. Tanwir F, Tariq A. Effect of glycemic conrol on periodontal status. Journal of the College of Physicians and Surgeons--Pakistan : JCPSP. 2012;22(6):371-4. Epub 2012/05/29. doi: 06.2012/jcpsp.371374. PubMed PMID: 22630096.

119. Teeuw WJ, Kosho MX, Poland DC, Gerdes VE, Loos BG. Periodontitis as a possible early sign of diabetes mellitus. BMJ open diabetes research & care. 2017;5(1):e000326. Epub 2017/03/21. doi: 10.1136/bmjdrc-2016-000326. PubMed PMID: 28316794; PubMed Central PMCID: PMCPMC5337701.

120. Tervonen T, Knuuttila M. Relation of diabetes control to periodontal pocketing and alveolar bone level. Oral surgery, oral medicine, and oral pathology. 1986;61(4):346-9. Epub 1986/04/01. PubMed PMID: 3458146.

121. Tervonen T, Oliver RC. Long-term control of diabetes mellitus and periodontitis. Journal of clinical periodontology. 1993;20(6):431-5. Epub 1993/07/01. PubMed PMID: 8349834.

122. Thorstensson H, Hugoson A. Periodontal disease experience in adult long-duration insulin-dependent diabetics. Journal of clinical periodontology. 1993;20(5):352-8. Epub 1993/05/01. PubMed PMID: 8501275.

123. Thorstensson H, Kuylenstierna J, Hugoson A. Medical status and complications in relation to periodontal disease experience in insulin-dependent diabetics. Journal of clinical periodontology. 1996;23(3 Pt 1):194-202. Epub 1996/03/01. PubMed PMID: 8707978.

124. Ueno M, Takeuchi S, Oshiro A, Shinada K, Ohara S, Kawaguchi Y. Association between diabetes mellitus and oral health status in Japanese adults. International journal of oral science. 2010;2(2):82-9. Epub 2010/08/27. doi: 10.4248/ijos10025. PubMed PMID: 20737934; PubMed Central PMCID: PMCPMC3733588.

125. Vadakkekuttical RJ, Kaushik PC, Mammen J, George JM. Does periodontal inflammation affect glycosylated haemoglobin level in otherwise systemically healthy individuals? - A hospital based study. Singapore dental journal. 2017;38:55-61. Epub 2017/12/13. doi: 10.1016/j.sdj.2017.08.002. PubMed PMID: 29229075.

126. Wahi S, Tripathi A, Wahi S, Mishra VD, Singh AP, Sinha N. Assessment of Levels of Glycosylated Hemoglobin in Patients with Periodontal Pathologies: A Comparative Study. The journal of contemporary dental practice. 2017;18(6):506-9. Epub 2017/06/18. PubMed PMID: 28621283.

127. Wellapuli N, Ekanayake L. Prevalence, severity and extent of chronic periodontitis among Sri Lankan adults. Community dental health. 2017;34(3):152-6. Epub 2017/09/06. doi: 10.1922/CDH_4070Wellapuli05. PubMed PMID: 28872809.

128. Wernicke K, Zeissler S, Mooren FC, Frech T, Hellmann S, Stiesch M, et al. Probing depth is an independent risk factor for HbA1c levels in diabetic patients under physical training: a cross-sectional pilot-study. BMC oral health. 2018;18(1):46. Epub 2018/03/20. doi: 10.1186/s12903-018-0491-9. PubMed PMID: 29548317; PubMed Central PMCID: PMCPMC5857102.

129. Wolff RE, Wolff LF, Michalowicz BS. A pilot study of glycosylated hemoglobin levels in periodontitis cases and healthy controls. Journal of periodontology. 2009;80(7):1057-61. Epub 2009/07/01. doi: 10.1902/jop.2009.080664. PubMed PMID: 19563284.

130. Zadik Y, Bechor R, Galor S, Levin L. Periodontal disease might be associated even with impaired fasting glucose. British dental journal. 2010;208(10):E20. Epub 2010/03/27. doi: 10.1038/sj.bdj.2010.291. PubMed PMID: 20339371.

131. Zhang DH, Yuan QN, Zabala PM, Zhang F, Ngo L, Darby IB. Diabetic and cardiovascular risk in patients diagnosed with periodontitis. Australian dental journal. 2015;60(4):455-62. Epub 2014/11/21. doi: 10.1111/adj.12253. PubMed PMID: 25410297.

132. Zielinski MB, Fedele D, Forman LJ, Pomerantz SC. Oral health in the elderly with non-insulin-dependent diabetes mellitus. Special care in dentistry : official publication of the American Association of Hospital Dentists, the Academy of Dentistry for the Handicapped, and the American Society for Geriatric Dentistry. 2002;22(3):94-8. Epub 2002/09/21. PubMed PMID: 12240893.

133. Ziukaite L, Slot DE, Cobb CM, Coucke W, Van der Weijden GA. Prevalence of diabetes among patients diagnosed with periodontitis: A retrospective cross-sectional study. International journal of dental hygiene. 2018;16(2):305-11. Epub 2017/05/04. doi: 10.1111/idh.12280. PubMed PMID: 28464544.

134. Abdo JA, Cirano FR, Casati MZ, Ribeiro FV, Giampaoli V, Casarin RCV, et al. Influence of Dyslipidemia and Diabetes Mellitus on Chronic Periodontal Disease. Journal of Periodontology. 2013;84(10):1401-8. doi: 10.1902/jop.2012.120366. PubMed PMID: WOS:000328685900009.

135. Basit HMB, Ahmed S, Malik U. The relations among obesity, diabetes mellitus and periodontitis in common people of Pakistan: A study analysis. International Journal of Advanced Biotechnology and Research. 2017;8(4):1973-7. PubMed PMID: WOS:000446389700139.

136. Belting CM, Hiniker JJ, Dummett CO. Influence of Diabetes Mellitus on Severity of Periodontal Disease. Journal of Periodontology. 1964;35(6):476-&. doi: 10.1902/jop.1964.35.6.476. PubMed PMID: WOS:A19646414B00002.

137. Deshmukh A, Sengupta N, Goswami S, Bandyopadhyay P, Saha S. An Analytical Approach to Explore Clinico-Biochemical Relationship between Type 2 Diabetes Mellitus (T2dm) and Oro-Dental Diseases Including Chronic Periodontitis. Journal of Evolution of Medical and Dental Sciences-Jemds. 2016;5(70):5118-22. doi: 10.14260/jemds/2016/1161. PubMed PMID: WOS:000394670000015.

138. Gupta S, Maharjan A, Dhami B, Amgain P, Katwal S, Adhikari B, et al. Status of Tobacco Smoking and Diabetes with Periodontal Disease. Journal of Nepal Medical Association. 2018;56(213):818-24. doi: 10.31729/jnma.3610. PubMed PMID: WOS:000449463300002.

139. Gupta VK, Hiremath SS, Malhotra S. Application of community periodontal index of treatment need (CPITN) in a group of insulin dependent diabetes mellitus (IDDM) patients. International Journal of Diabetes in Developing Countries. 2013;33(1):55-9. doi: 10.1007/s13410-012-0106-y. PubMed PMID: WOS:000314712800011.

140. Hamasha AAH, Almogbel L, Alshehri A, Alssafi F, Alghamdi H, Alajmi A, et al. Medical conditions associated with tooth loss among Saudi adults. Archives of Orofacial Science. 2018;13(1):16-21. PubMed PMID: WOS:000434839100003.

141. Hayden P, Buckley LA. Diabetes-Mellitus and Periodontal-Disease in an Irish Population. Journal of Periodontal Research. 1989;24(5):298-302. doi: 10.1111/j.1600-0765.1989.tb00873.x. PubMed PMID: WOS:A1989AX09800002.

142. Ilyas A, Saeed A, Akram S. Oral manifestations in Diabetic patients under treatment for Cardiomyopathy. Pakistan Journal of Medical & Health Sciences. 2018;12(1):180-2. PubMed PMID: WOS:000432248900053.

143. Javali MA, Khader MA, Al-Qahtani NA. Prevalence of Self-reported Medical Conditions among Dental Patients. Saudi journal of medicine & medical sciences. 2017;5(3):238-41. Epub 2017/09/01. doi: 10.4103/sjmms.sjmms_78_16. PubMed PMID: 30787795; PubMed Central PMCID: PMCPMC6298307.

144. Nichols C, Laster LL, Bodakgyovai LZ. Diabetes-Mellitus and Periodontal-Disease. Journal of Periodontology. 1978;49(2):85-8. doi: 10.1902/jop.1978.49.2.85. PubMed PMID: WOS:A1978EV07700007.

145. Nordin MM, Rahman SA, Raman RPC, Vaithilingam RD. Periodontal Status and Oral Health Knowledge among a Selected Population of Malaysian Type 2 Diabetics. Sains Malaysiana. 2014;43(8):1157-63. PubMed PMID: WOS:000343795600006.

146. Notohartojo IT, Suratri MAL. Teeth Brush, Fruit and Vegetables Consumption, Physical Activities, Diabetes Mellitus and Periodontal Tissue Health in Indonesia, Year 2013. Buletin Penelitian Sistem Kesehatan. 2016;19(4):219-25. doi: 10.22435/hsr.v19i4.6839.219-225. PubMed PMID: WOS:000404903100001.

147. Paraschiv C, Esanu I, Gavrilescu CM, Ghiuru R, Munteanu D, Manea P. Risk Factors for Periodontal Disease in Diabetic Patients. Romanian Journal of Oral Rehabilitation. 2018;10(3):199-204. PubMed PMID: WOS:000444858500025.

148. Peacock ME, Carson RE. Frequency of Self-Reported Medical Conditions in Periodontal Patients. Journal of Periodontology. 1995;66(11):1004-7. doi: 10.1902/jop.1995.66.11.1004. PubMed PMID: WOS:A1995TF54900012.

149. Ravindran R, Deepa MG, Sruthi AK, Kuruvila C, Priya S, Sunil S, et al. Evaluation of Oral Health in Type II Diabetes Mellitus Patients. Oral & Maxillofacial Pathology Journal. 2015;6(1):525-31. doi: 10.5005/jp-journals-10037-1030. PubMed PMID: WOS:000216491200001.

150. Shlossman M, Knowler WC, Pettitt DJ, Genco RJ. Type-2 Diabetes-Mellitus and Periodontal-Disease. Journal of the American Dental Association. 1990;121(4):532-6. doi: 10.14219/jada.archive.1990.0211. PubMed PMID: WOS:A1990ED21100024.

151. Trentin MS, De Carli JP, Ferreira MD, Gambin DJ, da Silva SO, Lisboa H. Prevalence and Severity of Periodontal Disease in Type 2 Diabetes Mellitus Patients: A Cross-Sectional Study. Bioscience Journal. 2018;34(4):1114-23. PubMed PMID: WOS:000441184700029.

152. Wang CX, Ma LL, Yang Y, Xu MR, Wang X, Feng XP, et al. Oral Health Knowledge, Attitudes, Behaviour and Oral Health Status of Chinese Diabetic Patients Aged 55 to 74 Years. The Chinese journal of dental research : the official journal of the Scientific Section of the Chinese Stomatological Association (CSA). 2018;21(4):267-73. Epub 2018/09/29. doi: 10.3290/j.cjdr.a41085. PubMed PMID: 30264043.

153. Al-Emadi A, Bissada N, Farah C, Siegel B, Al-Zaharani M. Systemic diseases among patients with and without alveolar bone loss. Quintessence international (Berlin, Germany : 1985). 2006;37(10):761-5. Epub 2006/11/03. PubMed PMID: 17078273.

154. Ngakinya NM, Ngotho BN, Kariuki HN, Kaimenyi JT. Periodontal health status of patients attending Diabetic Clinic at Kenyatta National Hospital, Nairobi, Kenya. Indian journal of dental research : official publication of Indian Society for Dental Research. 1997;8(4):105-10. Epub 1998/05/20. PubMed PMID: 9586523.

155. Sagana M, Duraisamy R, Jeevitha M. Correlation Between Diabetes and Completely Edentulous Condition-A Retrospective Study. Journal of Research in Medical and Dental Science. 2020;8(7):163-7. PubMed PMID: WOS:000595155900027.

156. Suzuki S, Noda T, Nishioka Y, Imamura T, Kamijo H, Sugihara N. Evaluation of tooth loss among patients with diabetes mellitus and using the National Database of Health Insurance Claims and Specific Health Checkups of Japan. International Dental Journal. 2020;70(4):308-15. doi: 10.1111/idj.12561. PubMed PMID: WOS:000516531600001.

157. Akinkugbe AA, Saraiya VM, Preisser JS, Offenbacher S, Beck JD. Bias in estimating the cross-sectional smoking, alcohol, obesity and diabetes associations with moderate-

severe periodontitis in the Atherosclerosis Risk in Communities study: comparison of full versus partial-mouth estimates. Journal of clinical periodontology. 2015;42(7):609-21. Epub 2015/06/17. doi: 10.1111/jcpe.12425. PubMed PMID: 26076661; PubMed Central PMCID: PMCPMC4509916.

158. Al-Khabbaz AK. Type 2 diabetes mellitus and periodontal disease severity. Oral health & preventive dentistry. 2014;12(1):77-82. Epub 2014/03/13. doi: 10.3290/j.ohpd.a31223. PubMed PMID: 24619786.

159. Al-Shammari KF, Al-Khabbaz AK, Al-Ansari JM, Neiva R, Wang HL. Risk indicators for tooth loss due to periodontal disease. Journal of periodontology. 2005;76(11):1910-8. Epub 2005/11/09. doi: 10.1902/jop.2005.76.11.1910. PubMed PMID: 16274310.

160. Ando A, Ohsawa M, Yaegashi Y, Sakata K, Tanno K, Onoda T, et al. Factors related to tooth loss among community-dwelling middle-aged and elderly Japanese men. Journal of epidemiology. 2013;23(4):301-6. Epub 2013/07/03. PubMed PMID: 23812101; PubMed Central PMCID: PMCPMC3709550.

161. Aoyama N, Suzuki JI, Kobayashi N, Hanatani T, Ashigaki N, Yoshida A, et al. Japanese Cardiovascular Disease Patients with Diabetes Mellitus Suffer Increased Tooth Loss in Comparison to Those without Diabetes Mellitus -A Cross-sectional Study. Internal medicine (Tokyo, Japan). 2018;57(6):777-82. Epub 2017/11/21. doi: 10.2169/internalmedicine.9578-17. PubMed PMID: 29151540; PubMed Central PMCID: PMCPMC5891513.

162. Awuti G, Younusi K, Li L, Upur H, Ren J. Epidemiological survey on the prevalence of periodontitis and diabetes mellitus in Uyghur adults from rural Hotan area in Xinjiang. Experimental diabetes research. 2012;2012:758921. Epub 2011/08/10. doi: 10.1155/2012/758921. PubMed PMID: 21826136; PubMed Central PMCID: PMCPMC3150776.

163. Bakhshandeh S, Murtomaa H, Mofid R, Vehkalahti MM, Suomalainen K. Periodontal treatment needs of diabetic adults. Journal of clinical periodontology. 2007;34(1):53-7. Epub 2007/01/25. doi: 10.1111/j.1600-051X.2006.01028.x. PubMed PMID: 17243996.

164. Beck JD, Moss KL, Morelli T, Offenbacher S. Periodontal profile class is associated with prevalent diabetes, coronary heart disease, stroke, and systemic markers of C-reactive protein and interleukin-6. Journal of periodontology. 2018;89(2):157-65. Epub 2018/03/10. doi: 10.1002/jper.17-0426. PubMed PMID: 29520823.

165. Bissong M, Azodo CC, Agbor MA, Nkuo-Akenji T, Fon PN. Oral health status of diabetes mellitus patients in Southwest Cameroon. Odonto-stomatologie tropicale = Tropical dental journal. 2015;38(150):49-57. Epub 2016/03/05. PubMed PMID: 26934773.

166. Botero JE, Yepes FL, Roldan N, Castrillon CA, Hincapie JP, Ochoa SP, et al. Tooth and periodontal clinical attachment loss are associated with hyperglycemia in patients with diabetes. Journal of periodontology. 2012;83(10):1245-50. Epub 2012/01/18. doi: 10.1902/jop.2012.110681. PubMed PMID: 22248217.

167. Branco-de-Almeida LS, Alves CM, Lopes FF, Pereira Ade F, Guerra RN, Pereira AL. Salivary IgA and periodontal treatment needs in diabetic patients. Brazilian oral research. 2011;25(6):550-5. Epub 2011/12/08. PubMed PMID: 22147237.

168. Campus G, Salem A, Uzzau S, Baldoni E, Tonolo G. Diabetes and periodontal disease: a case-control study. Journal of periodontology. 2005;76(3):418-25. Epub 2005/04/29. doi: 10.1902/jop.2005.76.3.418. PubMed PMID: 15857077.

169. Chatzopoulos GS, Cisneros A, Sanchez M, Lunos S, Wolff LF. Validity of self-reported periodontal measures, demographic characteristics, and systemic medical conditions. Journal of periodontology. 2018;89(8):924-32. Epub 2018/04/07. doi: 10.1002/jper.17-0586. PubMed PMID: 29624676.

170. Chatzopoulos GS, Cisneros A, Sanchez M, Wolff LF. Systemic medical conditions and periodontal status in older individuals. Special care in dentistry : official publication of the American Association of Hospital Dentists, the Academy of Dentistry for the Handicapped, and the American Society for Geriatric Dentistry. 2018. Epub 2018/09/02. doi: 10.1111/scd.12319. PubMed PMID: 30171823.

171. Chatzopoulos GS, Tsalikis L, Menexes G. Influence of Body Mass Index and Other Periodontitis-associated Risk Factors and Risk Indicators on Periodontal Treatment Needs: A Cross-sectional Study. Oral health & preventive dentistry. 2017;15(2):191-7. Epub 2017/03/23. doi: 10.3290/j.ohpd.a37933. PubMed PMID: 28322365.

172. Chen L, Wei B, Li J, Liu F, Xuan D, Xie B, et al. Association of periodontal parameters with metabolic level and systemic inflammatory markers in patients with type 2 diabetes. Journal of periodontology. 2010;81(3):364-71. Epub 2010/03/03. doi: 10.1902/jop.2009.090544. PubMed PMID: 20192862.

173. Choi YH, McKeown RE, Mayer-Davis EJ, Liese AD, Song KB, Merchant AT. Association between periodontitis and impaired fasting glucose and diabetes. Diabetes care. 2011;34(2):381-6. Epub 2011/01/11. doi: 10.2337/dc10-1354. PubMed PMID: 21216848; PubMed Central PMCID: PMCPMC3024353.

174. de Araujo Nobre M, Malo P. Prevalence of periodontitis, dental caries, and peri-implant pathology and their relation with systemic status and smoking habits: Results of an open-cohort study with 22009 patients in a private rehabilitation center. Journal of dentistry. 2017;67:36-42. Epub 2017/07/29. doi: 10.1016/j.jdent.2017.07.013. PubMed PMID: 28750777.

175. Delgado-Perez VJ, De La Rosa-Santillana R, Marquez-Corona ML, Avila-Burgos L, Islas-Granillo H, Minaya-Sanchez M, et al. Diabetes or hypertension as risk indicators for missing teeth experience: An exploratory study in a sample of Mexican adults. Nigerian journal of clinical practice. 2017;20(10):1335-41. Epub 2017/12/02. doi: 10.4103/njcp.njcp_52_17. PubMed PMID: 29192641.

176. Demmer RT, Kocher T, Schwahn C, Volzke H, Jacobs DR, Jr., Desvarieux M. Refining exposure definitions for studies of periodontal disease and systemic disease associations. Community dentistry and oral epidemiology. 2008;36(6):493-502. Epub 2008/04/22. doi: 10.1111/j.1600-0528.2008.00435.x. PubMed PMID: 18422705; PubMed Central PMCID: PMCPMC5651676.

177. Dhir S, Kumar V. Are cardiovascular risk parameters and glycemic levels associated with periodontitis in type 2 diabetes patients? A clinical study. Indian heart journal. 2018;70(3):430-2. Epub 2018/07/03. doi: 10.1016/j.ihj.2017.10.002. PubMed PMID: 29961463; PubMed Central PMCID: PMCPMC6034023.

178. Dolan TA, Gilbert GH, Ringelberg ML, Legler DW, Antonson DE, Foerster U, et al. Behavioral risk indicators of attachment loss in adult Floridians. Journal of clinical periodontology. 1997;24(4):223-32. Epub 1997/04/01. PubMed PMID: 9144044.

179. Eke PI, Wei L, Thornton-Evans GO, Borrell LN, Borgnakke WS, Dye B, et al. Risk Indicators for Periodontitis in US Adults: NHANES 2009 to 2012. Journal of periodontology.

2016;87(10):1174-85. Epub 2016/07/02. doi: 10.1902/jop.2016.160013. PubMed PMID: 27367420.

180. Emrich LJ, Shlossman M, Genco RJ. Periodontal disease in non-insulin-dependent diabetes mellitus. Journal of periodontology. 1991;62(2):123-31. Epub 1991/02/01. doi: 10.1902/jop.1991.62.2.123. PubMed PMID: 2027060.

181. Fernandes JK, Wiegand RE, Salinas CF, Grossi SG, Sanders JJ, Lopes-Virella MF, et al. Periodontal disease status in gullah african americans with type 2 diabetes living in South Carolina. Journal of periodontology. 2009;80(7):1062-8. Epub 2009/07/01. doi: 10.1902/jop.2009.080486. PubMed PMID: 19563285; PubMed Central PMCID: PMCPMC2862011.

182. Figueiredo A, Soares S, Lopes H, dos Santos JN, Ramalho LM, Cangussu MC, et al. Destructive periodontal disease in adult Indians from Northeast Brazil: cross-sectional study of prevalence and risk indicators. Journal of clinical periodontology. 2013;40(11):1001-6. Epub 2013/10/30. doi: 10.1111/jcpe.12147. PubMed PMID: 24164568.

183. Garcia D, Tarima S, Okunseri C. Periodontitis and glycemic control in diabetes: NHANES 2009 to 2012. Journal of periodontology. 2015;86(4):499-506. Epub 2014/11/28. doi: 10.1902/jop.2014.140364. PubMed PMID: 25427615.

184. Garcia-Perez A, Borges-Yanez SA, Jimenez-Corona A, Jimenez-Corona ME, Ponce-de-Leon S. Self-report of gingival problems and periodontitis in indigenous and non-indigenous populations in Chiapas, Mexico. International dental journal. 2016;66(2):105-12. Epub 2016/01/24. doi: 10.1111/idj.12213. PubMed PMID: 26800859.

185. Goulart AC, Armani F, Arap AM, Nejm T, Andrade JB, Bufarah HB, et al. Relationship between periodontal disease and cardiovascular risk factors among young and middle-aged Brazilians. Cross-sectional study. Sao Paulo medical journal = Revista paulista de medicina. 2017;135(3):226-33. Epub 2017/07/27. doi: 10.1590/1516-3180.2016.0357300117. PubMed PMID: 28746658.

186. Greenblatt AP, Salazar CR, Northridge ME, Kaplan RC, Taylor GW, Finlayson TL, et al. Association of diabetes with tooth loss in Hispanic/Latino adults: findings from the Hispanic Community Health Study/Study of Latinos. BMJ open diabetes research & care. 2016;4(1):e000211. Epub 2016/05/31. doi: 10.1136/bmjdrc-2016-000211. PubMed PMID: 27239319; PubMed Central PMCID: PMCPMC4873949.

187. Grossi SG, Zambon JJ, Ho AW, Koch G, Dunford RG, Machtei EE, et al. Assessment of risk for periodontal disease. I. Risk indicators for attachment loss. Journal of periodontology. 1994;65(3):260-7. Epub 1994/03/01. doi: 10.1902/jop.1994.65.3.260. PubMed PMID: 8164120.

188. Han DH, Lim S, Kim JB. The association of smoking and diabetes with periodontitis in a Korean population. Journal of periodontology. 2012;83(11):1397-406. Epub 2012/03/02. doi: 10.1902/jop.2012.110686. PubMed PMID: 22376209.

189. Han K, Park JB. Clinical implication of fasting glucose and systolic/diastolic blood pressure on the prevalence of periodontitis in non-diabetic and non-hypertensive adults using nationally representative data. Experimental and therapeutic medicine. 2018;16(2):671-8. Epub 2018/08/18. doi: 10.3892/etm.2018.6260. PubMed PMID: 30116321; PubMed Central PMCID: PMCPMC6090277.

190. Hastings JF, Vasquez E. Diabetes and Tooth Loss among Working-Age African Americans: A National Perspective. Social work in public health. 2017;32(7):443-51. Epub

2017/08/11. doi: 10.1080/19371918.2017.1358126. PubMed PMID: 28796580; PubMed Central PMCID: PMCPMC5881378.

191. Hodge PJ, Robertson D, Paterson K, Smith GL, Creanor S, Sherriff A. Periodontitis in non-smoking type 1 diabetic adults: a cross-sectional study. Journal of clinical periodontology. 2012;39(1):20-9. Epub 2011/11/19. doi: 10.1111/j.1600-051X.2011.01791.x. PubMed PMID: 22092931.

192. Holm NC, Belstrom D, Ostergaard JA, Schou S, Holmstrup P, Grauballe MB. Identification of Individuals With Undiagnosed Diabetes and Pre-Diabetes in a Danish Cohort Attending Dental Treatment. Journal of periodontology. 2016;87(4):395-402. Epub 2016/01/09. doi: 10.1902/jop.2016.150266. PubMed PMID: 26745612.

193. Hong JW, Noh JH, Kim DJ. The Prevalence and Associated Factors of Periodontitis According to Fasting Plasma Glucose in the Korean Adults: The 2012-2013 Korea National Health and Nutrition Examination Survey. Medicine. 2016;95(14):e3226. Epub 2016/04/09. doi: 10.1097/md.0000000000003226. PubMed PMID: 27057854; PubMed Central PMCID: PMCPMC4998770.

194. Jafer M. The Periodontal Status and Associated Systemic Health Problems among an Elderly Population Attending the Outpatient Clinics of a Dental School. The journal of contemporary dental practice. 2015;16(12):950-6. Epub 2015/01/01. PubMed PMID: 27018029.

195. Jiang Y, Okoro CA, Oh J, Fuller DL. Sociodemographic and health-related risk factors associated with tooth loss among adults in Rhode Island. Preventing chronic disease. 2013;10:E45. Epub 2013/03/30. doi: 10.5888/pcd10.110285. PubMed PMID: 23537519; PubMed Central PMCID: PMCPMC3614421.

196. Jung SH, Ryu JI, Jung DB. Association of total tooth loss with socio-behavioural health indicators in Korean elderly. Journal of oral rehabilitation. 2011;38(7):517-24. Epub 2010/12/02. doi: 10.1111/j.1365-2842.2010.02178.x. PubMed PMID: 21118289.

197. Jung YS, Shin MH, Kweon SS, Lee YH, Kim OJ, Kim YJ, et al. Periodontal disease associated with blood glucose levels in urban Koreans aged 50 years and older: the Dong-gu study. Gerodontology. 2015;32(4):267-73. Epub 2014/01/17. doi: 10.1111/ger.12107. PubMed PMID: 24428748.

198. Kapp JM, Boren SA, Yun S, LeMaster J. Diabetes and tooth loss in a national sample of dentate adults reporting annual dental visits. Preventing chronic disease. 2007;4(3):A59. Epub 2007/06/19. PubMed PMID: 17572963; PubMed Central PMCID: PMCPMC1955413.

199. Karikoski A, Murtomaa H, Ilanne-Parikka P. Assessment of periodontal treatment needs among adults with diabetes in Finland. International dental journal. 2002;52(2):75-80. Epub 2002/05/16. PubMed PMID: 12013254.

200. Katz J. Elevated blood glucose levels in patients with severe periodontal disease. Journal of clinical periodontology. 2001;28(7):710-2. Epub 2001/06/26. PubMed PMID: 11422596.

201. Kaur G, Holtfreter B, Rathmann W, Schwahn C, Wallaschofski H, Schipf S, et al. Association between type 1 and type 2 diabetes with periodontal disease and tooth loss. Journal of clinical periodontology. 2009;36(9):765-74. Epub 2009/07/23. doi: 10.1111/j.1600-051X.2009.01445.x. PubMed PMID: 19622096.

202. Khader YS. Factors associated with periodontal diseases in Jordan: principal component and factor analysis approach. Journal of oral science. 2006;48(2):77-84. Epub 2006/07/22. PubMed PMID: 16858136.

203. Kim DW, Park JC, Rim TT, Jung UW, Kim CS, Donos N, et al. Socioeconomic disparities of periodontitis in Koreans based on the KNHANES IV. Oral diseases. 2014;20(6):551-9. Epub 2013/09/17. doi: 10.1111/odi.12168. PubMed PMID: 24033864.

204. Klein BE, Klein R, Knudtson MD. Life-style correlates of tooth loss in an adult Midwestern population. Journal of public health dentistry. 2004;64(3):145-50. Epub 2004/09/03. PubMed PMID: 15341137.

205. Kongstad J, Enevold C, Christensen LB, Fiehn NE, Holmstrup P. Impact of Periodontitis Case Criteria: A Cross-Sectional Study of Lifestyle. Journal of periodontology. 2017;88(6):602-9. Epub 2017/01/28. doi: 10.1902/jop.2017.160426. PubMed PMID: 28128681.

206. Koo HS, Hong SM. Prevalence and Risk Factors for Periodontitis Among Patients with Metabolic Syndrome. Metabolic syndrome and related disorders. 2018;16(7):375-81. Epub 2018/06/13. doi: 10.1089/met.2018.0003. PubMed PMID: 29893616.

207. Kowall B, Holtfreter B, Volzke H, Schipf S, Mundt T, Rathmann W, et al. Pre-diabetes and well-controlled diabetes are not associated with periodontal disease: the SHIP Trend Study. Journal of clinical periodontology. 2015;42(5):422-30. Epub 2015/03/27. doi: 10.1111/jcpe.12391. PubMed PMID: 25808753.

208. Laguzzi PN, Schuch HS, Medina LD, de Amores AR, Demarco FF, Lorenzo S. Tooth loss and associated factors in elders: results from a national survey in Uruguay. Journal of public health dentistry. 2016;76(2):143-51. Epub 2015/10/16. doi: 10.1111/jphd.12123. PubMed PMID: 26465229.

209. Lee JH, Lee JS, Park JY, Choi JK, Kim DW, Kim YT, et al. Association of Lifestyle-Related Comorbidities With Periodontitis: A Nationwide Cohort Study in Korea. Medicine. 2015;94(37):e1567. Epub 2015/09/17. doi: 10.1097/md.0000000000001567. PubMed PMID: 26376407; PubMed Central PMCID: PMCPMC4635821.

210. Leung WK, Siu SC, Chu FC, Wong KW, Jin L, Sham AS, et al. Oral health status of low-income, middle-aged to elderly Hong Kong Chinese with type 2 diabetes mellitus. Oral health & preventive dentistry. 2008;6(2):105-18. Epub 2008/07/22. PubMed PMID: 18637388.

211. Levine ME, Kim JK, Crimmins EM. The role of physiological markers of health in the association between demographic factors and periodontal disease. Journal of periodontal research. 2013;48(3):367-72. Epub 2012/12/13. doi: 10.1111/jre.12016. PubMed PMID: 23231345; PubMed Central PMCID: PMCPMC3609891.

212. Luo H, Pan W, Sloan F, Feinglos M, Wu B. Forty-Year Trends in Tooth Loss Among American Adults With and Without Diabetes Mellitus: An Age-Period-Cohort Analysis. Preventing chronic disease. 2015;12:E211. Epub 2015/12/04. doi: 10.5888/pcd12.150309. PubMed PMID: 26632952; PubMed Central PMCID: PMCPMC4674438.

213. Marjanovic M, Buhlin K. Periodontal and systemic diseases among Swedish dental school patients - a retrospective register study. Oral health & preventive dentistry. 2013;11(1):49-55. Epub 2013/03/20. doi: 10.3290/j.ohpd.a29375. PubMed PMID: 23507681.

214. Najafipour H, Malek Mohammadi T, Rahim F, Haghdoost AA, Shadkam M, Afshari M. Association of oral health and cardiovascular disease risk factors "results from a community based study on 5900 adult subjects". ISRN cardiology. 2013;2013:782126. Epub 2013/08/21. doi: 10.1155/2013/782126. PubMed PMID: 23956878; PubMed Central PMCID: PMCPMC3727197.

215. Nand KY, Oommen AM, Chacko RK, Abraham VJ. Chronic periodontitis among diabetics and nondiabetics aged 35-65 years, in a rural block in Vellore, Tamil Nadu: A cross-sectional study. Journal of Indian Society of Periodontology. 2017;21(4):309-14. Epub 2018/02/20. doi: 10.4103/jisp.jisp_217_17. PubMed PMID: 29456306; PubMed Central PMCID: PMCPMC5813346.

216. Nesse W, Dijkstra PU, Abbas F, Spijkervet FK, Stijger A, Tromp JA, et al. Increased prevalence of cardiovascular and autoimmune diseases in periodontitis patients: a cross-sectional study. Journal of periodontology. 2010;81(11):1622-8. Epub 2010/06/30. doi: 10.1902/jop.2010.100058. PubMed PMID: 20583916.

217. Newton KM, Chaudhari M, Barlow WE, Inge RE, Theis MK, Spangler LA, et al. A population-based study of periodontal care among those with and without diabetes. Journal of periodontology. 2011;82(12):1650-6. Epub 2011/05/26. doi: 10.1902/jop.2011.100609. PubMed PMID: 21609255.

218. Noack B, Aslanhan Z, Boue J, Petig C, Teige M, Schaper F, et al. Potential association of paraoxonase-1, type 2 diabetes mellitus, and periodontitis. Journal of periodontology. 2013;84(5):614-23. Epub 2012/07/10. doi: 10.1902/jop.2012.120062. PubMed PMID: 22769439.

219. Ozcaka O, Becerik S, Bicakci N, Kiyak AH. Periodontal disease and systemic diseases in an older population. Archives of gerontology and geriatrics. 2014;59(2):474-9. Epub 2014/07/13. doi: 10.1016/j.archger.2014.05.011. PubMed PMID: 25015876.

220. Patel MH, Kumar JV, Moss ME. Diabetes and tooth loss: an analysis of data from the National Health and Nutrition Examination Survey, 2003-2004. Journal of the American Dental Association (1939). 2013;144(5):478-85. Epub 2013/05/02. PubMed PMID: 23633695.

221. Patino Marin N, Loyola Rodriguez JP, Medina Solis CE, Pontigo Loyola AP, Reyes Macias JF, Ortega Rosado JC, et al. Caries, periodontal disease and tooth loss in patients with diabetes mellitus types 1 and 2. Acta odontologica latinoamericana : AOL. 2008;21(2):127-33. Epub 2009/01/31. PubMed PMID: 19177848.

222. Pham TAV, Tran TTP. The interaction among obesity, Type 2 diabetes mellitus, and periodontitis in Vietnamese patients. Clinical and experimental dental research. 2018;4(3):63-71. Epub 2018/06/30. doi: 10.1002/cre2.106. PubMed PMID: 29955389; PubMed Central PMCID: PMCPMC6010795.

223. Plessas A, Robertson DP, Hodge PJ. Radiographic bone loss in a Scottish non-smoking type 1 diabetes mellitus population: A bitewing radiographic study. Journal of periodontology. 2018;89(9):1043-51. Epub 2018/05/17. doi: 10.1002/jper.16-0788. PubMed PMID: 29766516.

224. Pranckeviciene A, Siudikiene J, Ostrauskas R, Machiulskiene V. Severity of periodontal disease in adult patients with diabetes mellitus in relation to the type of diabetes. Biomedical papers of the Medical Faculty of the University Palacky, Olomouc, Czechoslovakia. 2014;158(1):117-23. Epub 2014/02/11. doi: 10.5507/bp.2013.098. PubMed PMID: 24510008.

225. Ribeiro LS, Santos JN, Vieira CL, Caramelli B, Ramalho LM, Cury PR. Association of dental infections with systemic diseases in Brazilian Native Indigenous: a cross-sectional study. Journal of the American Society of Hypertension : JASH. 2016;10(5):413-9. Epub 2016/04/04. doi: 10.1016/j.jash.2016.02.012. PubMed PMID: 27039160.

226. Roberts-Thomson KF, Do LG, Bartold PM, Daniels J, Grosse A, Meihubers S. Prevalence, extent and severity of severe periodontal destruction in an urban Aboriginal and Torres Strait Islander population. Australian dental journal. 2014;59(1):43-7. Epub 2014/02/08. doi: 10.1111/adj.12138. PubMed PMID: 24502510.

227. Sensorn W, Chatrchaiwiwatana S, Bumrerraj S. Relationship between diabetes mellitus and tooth loss in adults residing in Ubonratchathani province, Thailand. Journal of the Medical Association of Thailand = Chotmaihet thangphaet. 2012;95(12):1593-605. Epub 2013/02/09. PubMed PMID: 23390792.

228. Silva AM, Vargas AM, Ferreira EF, de Abreu MH. Periodontitis in individuals with diabetes treated in the public health system of Belo Horizonte, Brazil. Revista brasileira de epidemiologia = Brazilian journal of epidemiology. 2010;13(1):118-25. Epub 2010/08/05. PubMed PMID: 20683560.

229. Silvestre FJ, Miralles L, Llambes F, Bautista D, Sola-Izquierdo E, Hernandez-Mijares A. Type 1 diabetes mellitus and periodontal disease: relationship to different clinical variables. Medicina oral, patologia oral y cirugia bucal. 2009;14(4):E175-9. Epub 2009/03/21. PubMed PMID: 19300353.

230. Stoykova M, Musurlieva N, Boyadzhiev D. Risk factors for development of chronic periodontitis in Bulgarian patients (pilot research). Biotechnology, biotechnological equipment. 2014;28(6):1150-4. Epub 2015/05/29. doi: 10.1080/13102818.2014.974328. PubMed PMID: 26019602; PubMed Central PMCID: PMCPMC4433785.

231. Taboza ZA, Costa KL, Silveira VR, Furlaneto FA, Montenegro R, Jr., Russell S, et al. Periodontitis, edentulism and glycemic control in patients with type 2 diabetes: a cross-sectional study. BMJ open diabetes research & care. 2018;6(1):e000453. Epub 2018/04/03. doi: 10.1136/bmjdrc-2017-000453. PubMed PMID: 29607049; PubMed Central PMCID: PMCPMC5873546.

232. Torrungruang K, Ongphiphadhanakul B, Jitpakdeebordin S, Sarujikumjornwatana S. Mediation analysis of systemic inflammation on the association between periodontitis and glycaemic status. Journal of clinical periodontology. 2018;45(5):548-56. Epub 2018/03/04. doi: 10.1111/jcpe.12884. PubMed PMID: 29500831.

233. Torrungruang K, Tamsailom S, Rojanasomsith K, Sutdhibhisal S, Nisapakultorn K, Vanichjakvong O, et al. Risk indicators of periodontal disease in older Thai adults. Journal of periodontology. 2005;76(4):558-65. Epub 2005/04/29. doi: 10.1902/jop.2005.76.4.558. PubMed PMID: 15857096.

234. Tsai C, Hayes C, Taylor GW. Glycemic control of type 2 diabetes and severe periodontal disease in the US adult population. Community dentistry and oral epidemiology. 2002;30(3):182-92. Epub 2002/05/10. PubMed PMID: 12000341.

235. Wakai K, Kawamura T, Umemura O, Hara Y, Machida J, Anno T, et al. Associations of medical status and physical fitness with periodontal disease. Journal of clinical periodontology. 1999;26(10):664-72. Epub 1999/10/16. PubMed PMID: 10522778.

236. Wang TT, Chen TH, Wang PE, Lai H, Lo MT, Chen PY, et al. A population-based study on the association between type 2 diabetes and periodontal disease in 12,123 middle-

aged Taiwanese (KCIS No. 21). Journal of clinical periodontology. 2009;36(5):372-9. Epub 2009/05/08. doi: 10.1111/j.1600-051X.2009.01386.x. PubMed PMID: 19419435.

237. Wellapuli N, Ekanayake L. Risk factors for chronic periodontitis in Sri Lankan adults: a population based case-control study. BMC research notes. 2017;10(1):460. Epub 2017/09/09. doi: 10.1186/s13104-017-2778-3. PubMed PMID: 28882173; PubMed Central PMCID: PMCPMC5590132.

238. Zhang Q, Li Z, Wang C, Shen T, Yang Y, Chotivichien S, et al. Prevalence and predictors for periodontitis among adults in China, 2010. Global health action. 2014;7:24503. Epub 2014/07/11. doi: 10.3402/gha.v7.24503. PubMed PMID: 25008055; PubMed Central PMCID: PMCPMC4090366.

239. Zuk A, Quinonez C, Lebenbaum M, Rosella LC. The association between undiagnosed glycaemic abnormalities and cardiometabolic risk factors with periodontitis: results from 2007-2009 Canadian Health Measures Survey. Journal of clinical periodontology. 2017;44(2):132-41. Epub 2016/12/29. doi: 10.1111/jcpe.12684. PubMed PMID: 28028834.

240. Zuk AM, Quinonez CR, Saarela O, Demmer RT, Rosella LC. Joint effects of serum vitamin D insufficiency and periodontitis on insulin resistance, pre-diabetes, and type 2 diabetes: results from the National Health and Nutrition Examination Survey (NHANES) 2009-2010. BMJ open diabetes research & care. 2018;6(1):e000535. Epub 2018/08/04. doi: 10.1136/bmjdrc-2018-000535. PubMed PMID: 30073089; PubMed Central PMCID: PMCPMC6067347.

241. Ahmad B, Ahmad O, Afzal M. The Etiology of Tooth Loss and Risk Factors Causing Periodontal Disease. Indo American Journal of Pharmaceutical Sciences. 2018;5(12):14009-14. doi: 10.5281/zenodo.1841775. PubMed PMID: WOS:000454558500078.

242. Bakhshandeh S, Murtomaa H, Vehkalahti M, Mofid R, Suomalainen K. Dental findings in diabetic adults. Caries Research. 2008;42(1):14-8. doi: 10.1159/000111745. PubMed PMID: WOS:000252706700003.

243. de Miguel-Infante A, Martinez-Huedo MA, Mora-Zamorano E, Hernandez-Barrera V, Jimenez-Trujillo I, de Burgos-Lunar C, et al. Periodontal disease in adults with diabetes, prevalence and risk factors. Results of an observational study. International journal of clinical practice. 2018:e13294. Epub 2018/11/18. doi: 10.1111/ijcp.13294. PubMed PMID: 30444571.

244. Dhir S, Wangnoo S, Kumar V. Impact of Glycemic Levels in Type 2 Diabetes on Periodontitis. Indian journal of endocrinology and metabolism. 2018;22(5):672-7. Epub 2018/10/09. doi: 10.4103/ijem.IJEM_566_17. PubMed PMID: 30294579; PubMed Central PMCID: PMCPMC6166544.

245. Emrich LJ, Shlossman M, Genco RJ. Periodontal-Disease in Non-Insulin-Dependent Diabetes-Mellitus. Journal of Periodontology. 1991;62(2):123-31. doi: 10.1902/jop.1991.62.2.123. PubMed PMID: WOS:A1991EX52900005.

246. Izuora K, Yousif A, Allenback G, Gewelber C, Neubauer M. Relationship between dental loss and health outcomes among hospitalized patients with and without diabetes. Journal of investigative medicine : the official publication of the American Federation for Clinical Research. 2019;67(3):669-73. Epub 2018/10/23. doi: 10.1136/jim-2018-000842. PubMed PMID: 30344140.

247. Kurahashi T, Kitagawa M, Matsukubo T. Factors Associated with Number of Present Teeth in Adults in Japanese Urban City. Bulletin of Tokyo Dental College. 2017;58(2):85-94. doi: 10.2209/tdcpublication.2016-2200. PubMed PMID: WOS:000415866600002.

248. Montero E, Carasol M, Fernandez-Meseguer A, Calvo-Bonacho E, Garcia-Margallo MT, Sanz M, et al. Prediabetes and diabetes prevalence in the Workers' Oral Health Study. Clinical oral investigations. 2019. Epub 2019/03/02. doi: 10.1007/s00784-019-02875-3. PubMed PMID: 30820824.

249. Rikawarastuti, Anggreni E, Ngatemi. Diabetes Melitus and Severity of Periodontal Tissue. Kesmas-National Public Health Journal. 2015;9(3):277-81. PubMed PMID: WOS:000410070200012.

250. Simila T, Auvinen J, Puukka K, Keinanen-Kiukaanniemi S, Virtanen JI. Impaired glucose metabolism is associated with tooth loss in middle-aged adults: The Northern Finland Birth Cohort Study 1966. Diabetes research and clinical practice. 2018;142:110-9. Epub 2018/06/02. doi: 10.1016/j.diabres.2018.05.035. PubMed PMID: 29857092.

251. Tsai C, Hayes C, Taylor GW. Glycemic control of type 2 diabetes and severe periodontitis in the US adult population. Journal of Dental Research. 2000;79:625-. PubMed PMID: WOS:000084937003841.

252. Leung WK, Movva LR, Wong MC, Corbet EF, Siu SC, Kawamura M. Health behaviour, metabolic control and periodontal status in medically treated Chinese with type 2 diabetes mellitus. Annals of the Royal Australasian College of Dental Surgeons. 2008;19:102-10. Epub 2008/06/01. PubMed PMID: 22073462.

253. Shlossman M, Knowler WC, Pettitt DJ, Genco RJ. Type 2 diabetes mellitus and periodontal disease. Journal of the American Dental Association (1939). 1990;121(4):532-6. Epub 1990/10/01. PubMed PMID: 2212346.

254. Thorstensson H. Periodontal disease in adult insulin-dependent diabetics. Swedish dental journal Supplement. 1995;107:1-68. Epub 1995/01/01. PubMed PMID: 7638766.

255. Moore PA. The diabetes-oral health connection. Compendium of continuing education in dentistry (Jamesburg, NJ : 1995). 2002;23(12 Suppl):14-20. Epub 2003/06/07. PubMed PMID: 12789997.

256. AlShwaimi E, Idrees M, Berri Z, El-Sakka H, Kujan O. Association between Diabetes Mellitus and Periodontal Diseases: A Survey of the Opinions of Dental Professionals. Medical Principles and Practice. 2019;28(2):141-9. doi: 10.1159/000495881. PubMed PMID: WOS:000467682900006.

257. Assiri KI, Sandeepa NC, Asiri RS, Mulawi SA, Najmi SM, Srivastava KC. Assessment of Oral-Systemic Disease Association amongst Dental Patients: A Retrospective Panoramic Radiographic Study. J Contemp Dent Pract. 2020;21(7):748-55. Epub 2020/10/07. PubMed PMID: 33020357.

258. Chen HH, Chen DY, Huang LG, Chen YM, Hsieh CW, Hung WT, et al. Association between periodontitis and the risk of inadequate disease control in patients with rheumatoid arthritis under biological treatment. J Clin Periodontol. 2020;47(2):148-59. Epub 2019/11/05. doi: 10.1111/jcpe.13213. PubMed PMID: 31677352.

259. Cozier YC, Heaton B, Bethea TN, Freudenheim JL, Garcia RI, Rosenberg L. Predictors of self-reported oral health in the Black Women's Health Study. Journal of Public

Health Dentistry. 2020;80(1):70-8. doi: 10.1111/jphd.12351. PubMed PMID: WOS:000502670300001.

260. Dar-Odeh N, Borzangy S, Babkair H, Farghal L, Shahin G, Fadhlalmawla S, et al. Association of Dental Caries, Retained Roots, and Missing Teeth with Physical Status, Diabetes Mellitus and Hypertension in Women of the Reproductive Age. International Journal of Environmental Research and Public Health. 2019;16(14). doi: 10.3390/ijerph16142565. PubMed PMID: WOS:000480659300118.

261. de Miguel-Infante A, Martinez-Huedo MA, Mora-Zamorano E, Hernandez-Barrera V, Jimenez-Trujillo I, de Burgos-Lunar C, et al. Periodontal disease in adults with diabetes, prevalence and risk factors. Results of an observational study. International Journal of Clinical Practice. 2019;73(3). doi: 10.1111/ijcp.13294. PubMed PMID: WOS:000462615600002.

262. Dhir S, Kumar V. Are cardiovascular risk parameters and glycemic levels associated with periodontitis in type 2 diabetes patients? A clinical study. Indian Heart Journal. 2018;70(3):430-2. doi: 10.1016/j.ihj.2017.10.002. PubMed PMID: WOS:000487586400019.

263. Furuta M, Fukai K, Aida J, Shimazaki Y, Ando Y, Miyazaki H, et al. Periodontal status and self-reported systemic health of periodontal patients regularly visiting dental clinics in the 8020 Promotion Foundation Study of Japanese Dental Patients. J Oral Sci. 2019;61(2):238-45. Epub 2019/06/21. doi: 10.2334/josnusd.18-0128. PubMed PMID: 31217373.

264. Izuora K, Yousif A, Allenback G, Gewelber C, Neubauer M. Relationship between dental loss and health outcomes among hospitalized patients with and without diabetes. Journal of Investigative Medicine. 2019;67(3):669-73. doi: 10.1136/jim-2018-000842. PubMed PMID: WOS:000471860800004.

265. Janakiram C, Taha F, Joseph J, Ramanarayanan V. Assessment of Common Risk Factors Between Oral Diseases and Non-communicable Diseases in a Hospital-based Population in Kerala, India-A Cross-sectional Study. Journal of Clinical and Diagnostic Research. 2019;13(3):ZC16-ZC20. doi: 10.7860/JCDR/2019/38182.12712. PubMed PMID: WOS:000460573100019.

266. Kim TY, Yeun YR, Kim HY. Effect of diabetes index on periodontal disease in Korean adults. Ethiopian Journal of Health Development. 2020;34:78-83. PubMed PMID: WOS:000602486500010.

267. Mataftsi M, Koukos G, Sakellari D. Prevalence of undiagnosed diabetes and pre-diabetes in chronic periodontitis patients assessed by an HbA1c chairside screening protocol. Clin Oral Investig. 2019;23(12):4365-70. Epub 2019/04/11. doi: 10.1007/s00784-019-02888-y. PubMed PMID: 30968241.

268. Obradovic R, Igic M, Mitic A, Pejcic A, Boskovic M, Todorovic K. Diabetes Mellitus as a Risk Factor for Periodontal Disease Development. Journal of Environmental Protection and Ecology. 2019;20(3):1487-95. PubMed PMID: WOS:000497992700046.

269. Philips KH, Zhang S, Moss K, Ciarrocca K, Beck JD. Periodontal disease, undiagnosed diabetes, and body mass index: Implications for diabetes screening by dentists. J Am Dent Assoc. 2021;152(1):25-35. Epub 2020/12/02. doi: 10.1016/j.adaj.2020.09.002. PubMed PMID: 33256949.

270. Puscasu CG, Dumea E, Petcu LC. Cardiovascular Disease and Diabetes, Potential Risk Factors for Periodontal Disease. Acta Medica Mediterranea. 2019;35(6):3177-82. doi: 10.19193/0393-6384_2019_6_499. PubMed PMID: WOS:000496261400046.

271. Rawal I, Ghosh S, Hameed SS, Shivashankar R, Ajay VS, Patel SA, et al. Association between poor oral health and diabetes among Indian adult population: potential for integration with NCDs. BMC Oral Health. 2019;19(1):191. Epub 2019/08/21. doi: 10.1186/s12903-019-0884-4. PubMed PMID: 31429749; PubMed Central PMCID: PMCPMC6701092.

272. Susin C, Dalla Vecchia CF, Oppermann RV, Haugejorden O, Albandar JM. Periodontal attachment loss in an urban population of Brazilian adults: effect of demographic, behavioral, and environmental risk indicators. J Periodontol. 2004;75(7):1033-41. Epub 2004/09/03. doi: 10.1902/jop.2004.75.7.1033. PubMed PMID: 15341364.

273. Wahab NA, Majid, II, Taib H. Periodontal Disease in Patients with Type 2 Diabetes and Its Relationship with Dry Mouth Condition. Archives of Orofacial Science. 2019;14(2):77-86. doi: 10.21315/aos2019.14.2.380. PubMed PMID: WOS:000508663700002.

274. Zuk AM, Quinonez CR, Saarela O, Demmer RT, Rosella LC. Joint effects of serum vitamin D insufficiency and periodontitis on insulin resistance, pre-diabetes, and type 2 diabetes: results from the National Health and Nutrition Examination Survey (NHANES) 2009-2010. Bmj Open Diabetes Research & Care. 2018;6(1). doi: 10.1136/bmjdrc-2018-000535. PubMed PMID: WOS:000506239800005.

275. Ajita M, Karan P, Vivek G, S MA, Anuj M. Periodontal disease and type 1 diabetes mellitus: associations with glycemic control and complications: an Indian perspective. Diabetes & metabolic syndrome. 2013;7(2):61-3. Epub 2013/05/18. doi: 10.1016/j.dsx.2013.03.001. PubMed PMID: 23680241.

276. Al-Khabbaz AK, Al-Shammari KF, Hasan A, Abdul-Rasoul M. Periodontal health of children with type 1 diabetes mellitus in Kuwait: a case-control study. Medical principles and practice : international journal of the Kuwait University, Health Science Centre. 2013;22(2):144-9. Epub 2012/10/19. doi: 10.1159/000342624. PubMed PMID: 23075471; PubMed Central PMCID: PMCPMC5586723.

277. Apoorva SM, Sridhar N, Suchetha A. Prevalence and severity of periodontal disease in type 2 diabetes mellitus (non-insulin-dependent diabetes mellitus) patients in Bangalore city: An epidemiological study. Journal of Indian Society of Periodontology. 2013;17(1):25-9. Epub 2013/05/02. doi: 10.4103/0972-124x.107470. PubMed PMID: 23633768; PubMed Central PMCID: PMCPMC3636938.

278. Boland MR, Hripcsak G, Albers DJ, Wei Y, Wilcox AB, Wei J, et al. Discovering medical conditions associated with periodontitis using linked electronic health records. Journal of clinical periodontology. 2013;40(5):474-82. Epub 2013/03/19. doi: 10.1111/jcpe.12086. PubMed PMID: 23495669; PubMed Central PMCID: PMCPMC3690348.

279. Chavada MG, Shah HM, Jethal BS. Influence of diabetes mellitus on periodontal disease. Indian journal of dental research : official publication of Indian Society for Dental Research. 1993;4(2):55-8. Epub 1993/04/01. PubMed PMID: 8180284.

280. Chrysanthakopoulos NA, Chrysanthakopoulos PA. Association between indices of clinically-defined periodontitis and self-reported history of systemic medical conditions. Journal of investigative and clinical dentistry. 2016;7(1):27-36. Epub 2014/07/23. doi: 10.1111/jicd.12119. PubMed PMID: 25048420.

281. Dumitrescu AL. Occurrence of self-reported systemic medical conditions in patients with periodontal disease. Romanian journal of internal medicine = Revue roumaine de medecine interne. 2006;44(1):35-48. Epub 2007/01/24. PubMed PMID: 17236286.

282. Galea H, Aganovic I, Aganovic M. The dental caries and periodontal disease experience of patients with early onset insulin dependent diabetes. International dental journal. 1986;36(4):219-24. Epub 1986/12/01. PubMed PMID: 3468081.

283. Knight ET, Leichter JW, Tawse-Smith A, Thomson WM. Quantifying the Association Between Self-Reported Diabetes and Periodontitis in the New Zealand Population. Journal of periodontology. 2015;86(8):945-54. Epub 2015/04/24. doi: 10.1902/jop.2015.150048. PubMed PMID: 25903984.

284. Mohamed HG, Idris SB, Ahmed MF, Boe OE, Mustafa K, Ibrahim SO, et al. Association between oral health status and type 2 diabetes mellitus among Sudanese adults: a matched case-control study. PloS one. 2013;8(12):e82158. Epub 2013/12/19. doi: 10.1371/journal.pone.0082158. PubMed PMID: 24349205; PubMed Central PMCID: PMCPMC3859584.

285. Moore PA, Weyant RJ, Mongelluzzo MB, Myers DE, Rossie K, Guggenheimer J, et al. Type 1 diabetes mellitus and oral health: assessment of periodontal disease. Journal of periodontology. 1999;70(4):409-17. Epub 1999/05/18. doi: 10.1902/jop.1999.70.4.409. PubMed PMID: 10328653.

286. Natto ZS, Al-Zahrani MS. Periodontal bone loss and self-reported medical conditions in a dental school patient population. Journal of the International Academy of Periodontology. 2010;12(4):104-9. Epub 2010/12/07. PubMed PMID: 21128528.

287. Oberoi SS, Harish Y, Hiremath S, Puranik M. A cross-sectional survey to study the relationship of periodontal disease with cardiovascular disease, respiratory disease, and diabetes mellitus. Journal of Indian Society of Periodontology. 2016;20(4):446-52. Epub 2017/03/17. doi: 10.4103/0972-124x.186946. PubMed PMID: 28298829; PubMed Central PMCID: PMCPMC5341322.

288. Persson RE, Hollender LG, MacEntee MI, Wyatt CC, Kiyak HA, Persson GR. Assessment of periodontal conditions and systemic disease in older subjects. Journal of clinical periodontology. 2003;30(3):207-13. Epub 2003/03/13. PubMed PMID: 12631178.

289. Randolph WM, Ostir GV, Markides KS. Prevalence of tooth loss and dental service use in older Mexican Americans. Journal of the American Geriatrics Society. 2001;49(5):585-9. Epub 2001/05/31. PubMed PMID: 11380751.

290. Sperr M, Kundi M, Tursic V, Bristela M, Moritz A, Andrukhov O, et al. Prevalence of Comorbidities in Periodontitis Patients Compared to the General Austrian Population. Journal of periodontology. 2017:1-13. Epub 2017/08/29. doi: 10.1902/jop.2017.170333. PubMed PMID: 28844189.

291. Susanto H, Nesse W, Dijkstra PU, Agustina D, Vissink A, Abbas F. Periodontitis prevalence and severity in Indonesians with type 2 diabetes. Journal of periodontology. 2011;82(4):550-7. Epub 2010/10/12. doi: 10.1902/jop.2010.100285. PubMed PMID: 20932156.

292. Syrjala AM, Ylostalo P, Niskanen MC, Knuuttila ML. Role of smoking and HbA1c level in periodontitis among insulin-dependent diabetic patients. Journal of clinical periodontology. 2003;30(10):871-5. Epub 2004/01/09. PubMed PMID: 14710767.

293. Cheema S, Maisonneuve P, Al-Thani MH, Al-Thani AAM, Abraham A, Al-Mannai GA, et al. Oral health behavior and factors associated with poor oral status in Qatar: results from a national health survey. Journal of Public Health Dentistry. 2017;77(4):308-16. doi: 10.1111/jphd.12209. PubMed PMID: WOS:000418429800005.

294. Sperr M, Kundi M, Tursic V, Bristela M, Moritz A, Andrukhov O, et al. Prevalence of comorbidities in periodontitis patients compared with the general Austrian population. Journal of Periodontology. 2018;89(1):19-27. doi: 10.1902/jop.2017.170333. PubMed PMID: WOS:000424513500002.

295. Ghalaut P, Sharma TK, Ghalaut VS, Singh R, Ghalaut PS. Glycohemoglobin levels with severity of periodontitis in non-diabetic population. Clinical laboratory. 2013;59(5-6):491-5. Epub 2013/07/20. PubMed PMID: 23865346.

296. Campus G, Salem A, Milia E, Baldoni E, Luglie P. Periodontal conditions in type 2 diabetes in an Italian population. A case control study. Journal of Dental Research. 2003;82:B287-B. PubMed PMID: WOS:000202893602643.

297. Abariga SA, Whitcomb BW. Periodontitis and gestational diabetes mellitus: a systematic review and meta-analysis of observational studies. BMC pregnancy and childbirth. 2016;16(1):344. Epub 2016/11/09. doi: 10.1186/s12884-016-1145-z. PubMed PMID: 27825315; PubMed Central PMCID: PMCPMC5101727.

298. Abbass MM, Korany NS, Salama AH, Dmytryk JJ, Safiejko-Mroczka B. The relationship between receptor for advanced glycation end products expression and the severity of periodontal disease in the gingiva of diabetic and non diabetic periodontitis patients. Archives of oral biology. 2012;57(10):1342-54. Epub 2012/07/17. doi: 10.1016/j.archoralbio.2012.06.007. PubMed PMID: 22795565.

299. Acharya AB, Thakur S, Muddapur MV. Evaluation of serum interleukin-10 levels as a predictor of glycemic alteration in chronic periodontitis and type 2 diabetes mellitus. Journal of Indian Society of Periodontology. 2015;19(4):388-92. Epub 2015/09/24. doi: 10.4103/0972-124x.150876. PubMed PMID: 26392685; PubMed Central PMCID: PMCPMC4555794.

300. Aggarwal A, Panat SR. Oral health behavior and HbA1c in Indian adults with type 2 diabetes. Journal of oral science. 2012;54(4):293-301. Epub 2012/12/12. PubMed PMID: 23221154.

301. Alhabashneh R, Khader Y, Herra Z, Asa'ad F, Assad F. The association between periodontal disease and metabolic syndrome among outpatients with diabetes in Jordan. Journal of diabetes and metabolic disorders. 2015;14:67. Epub 2015/08/19. doi: 10.1186/s40200-015-0192-8. PubMed PMID: 26280008; PubMed Central PMCID: PMCPMC4537782.

302. Allen EM, Matthews JB, DJ OH, Griffiths HR, Chapple IL. Oxidative and inflammatory status in Type 2 diabetes patients with periodontitis. Journal of clinical periodontology. 2011;38(10):894-901. Epub 2011/09/03. doi: 10.1111/j.1600-051X.2011.01764.x. PubMed PMID: 21883360.

303. Altamash M, Klinge B, Engstrom PE. Periodontal treatment and HbA1c levels in subjects with diabetes mellitus. Journal of oral rehabilitation. 2016;43(1):31-8. Epub 2015/09/04. doi: 10.1111/joor.12339. PubMed PMID: 26332936.

304. Alves C, Menezes R, Brandao M. Salivary flow and dental caries in Brazilian youth with type 1 diabetes mellitus. Indian journal of dental research : official publication of Indian Society for Dental Research. 2012;23(6):758-62. Epub 2013/05/08. doi: 10.4103/0970-9290.111254. PubMed PMID: 23649059.

305. Arana C, Moreno-Fernandez AM, Gomez-Moreno G, Morales-Portillo C, Serrano-Olmedo I, de la Cuesta Mayor MC, et al. Increased salivary oxidative stress parameters in

patients with type 2 diabetes: Relation with periodontal disease. Endocrinologia, diabetes y nutricion. 2017;64(5):258-64. Epub 2017/05/13. doi: 10.1016/j.endinu.2017.03.005. PubMed PMID: 28495321.

306. Arora N, Papapanou PN, Rosenbaum M, Jacobs DR, Jr., Desvarieux M, Demmer RT. Periodontal infection, impaired fasting glucose and impaired glucose tolerance: results from the Continuous National Health and Nutrition Examination Survey 2009-2010. Journal of clinical periodontology. 2014;41(7):643-52. Epub 2014/04/09. doi: 10.1111/jcpe.12258. PubMed PMID: 24708451; PubMed Central PMCID: PMCPMC4072528.

307. Boillot A, Bouchard P, Moss K, Offenbacher S, Czernichow S. Periodontitis and retinal microcirculation in the Atherosclerosis Risk in Communities study. Journal of clinical periodontology. 2015;42(4):342-9. Epub 2015/03/03. doi: 10.1111/jcpe.12388. PubMed PMID: 25728988.

308. Botero JE, Yepes FL, Ochoa SP, Hincapie JP, Roldan N, Ospina CA, et al. Effects of periodontal non-surgical therapy plus azithromycin on glycemic control in patients with diabetes: a randomized clinical trial. Journal of periodontal research. 2013;48(6):706-12. Epub 2013/02/28. doi: 10.1111/jre.12058. PubMed PMID: 23441920.

309. Cairo F, Nieri M, Gori AM, Rotundo R, Castellani S, Abbate R, et al. Periodontal variables may predict sub-clinical atherosclerosis and systemic inflammation in young adults. A cross-sectional study. European journal of oral implantology. 2009;2(2):125-33. Epub 2010/05/15. PubMed PMID: 20467611.

310. Camen GC, Caraivan O, Olteanu M, Camen A, Bunget A, Popescu FC, et al. Inflammatory reaction in chronic periodontopathies in patients with diabetes mellitus. Histological and immunohistochemical study. Romanian journal of morphology and embryology = Revue roumaine de morphologie et embryologie. 2012;53(1):55-60. Epub 2012/03/08. PubMed PMID: 22395500.

311. Castrillon CA, Hincapie JP, Yepes FL, Roldan N, Moreno SM, Contreras A, et al. Occurrence of red complex microorganisms and Aggregatibacter actinomycetemcomitans in patients with diabetes. Journal of investigative and clinical dentistry. 2015;6(1):25-31. Epub 2013/07/17. doi: 10.1111/jicd.12051. PubMed PMID: 23857867.

312. Chaudhari M, Hubbard R, Reid RJ, Inge R, Newton KM, Spangler L, et al. Evaluating components of dental care utilization among adults with diabetes and matched controls via hurdle models. BMC oral health. 2012;12:20. Epub 2012/07/11. doi: 10.1186/1472-6831-12-20. PubMed PMID: 22776352; PubMed Central PMCID: PMCPMC3528407.

313. Corlan Puscu D, Ciuluvica RC, Anghel A, Malaescu GD, Ciursas AN, Popa GV, et al. Periodontal disease in diabetic patients - clinical and histopathological aspects. Romanian journal of morphology and embryology = Revue roumaine de morphologie et embryologie. 2016;57(4):1323-9. Epub 2017/02/09. PubMed PMID: 28174799.

314. Darby I, Phan L, Post M. Periodontal health of dental clients in a community health setting. Australian dental journal. 2012;57(4):486-92. Epub 2012/11/29. doi: 10.1111/j.1834-7819.2012.01723.x. PubMed PMID: 23186575.

315. Demmer RT, Desvarieux M, Holtfreter B, Jacobs DR, Jr., Wallaschofski H, Nauck M, et al. Periodontal status and A1C change: longitudinal results from the study of health in Pomerania (SHIP). Diabetes care. 2010;33(5):1037-43. Epub 2010/02/27. doi: 10.2337/dc09-1778. PubMed PMID: 20185742; PubMed Central PMCID: PMCPMC2858171.

316. Develioglu H, Ozdemir H, Bostanci V. Comparative analysis of the blood flow values of patients with type 2 diabetes mellitus presenting with chronic periodontitis, patients with chronic periodontitis only and healthy individuals. The West Indian medical journal. 2014;63(4):359-63. Epub 2014/11/28. doi: 10.7727/wimj.2013.174. PubMed PMID: 25429482; PubMed Central PMCID: PMCPMC4663915.

317. Eklund SA, Burt BA. Risk factors for total tooth loss in the United States; longitudinal analysis of national data. Journal of public health dentistry. 1994;54(1):5-14. Epub 1994/01/01. PubMed PMID: 8164192.

318. Ervasti T, Knuuttila M, Pohjamo L, Haukipuro K. Relation between control of diabetes and gingival bleeding. Journal of periodontology. 1985;56(3):154-7. Epub 1985/03/01. doi: 10.1902/jop.1985.56.3.154. PubMed PMID: 3872936.

319. Gatke D, Holtfreter B, Biffar R, Kocher T. Five-year change of periodontal diseases in the Study of Health in Pomerania (SHIP). Journal of clinical periodontology. 2012;39(4):357-67. Epub 2012/03/06. doi: 10.1111/j.1600-051X.2011.01849.x. PubMed PMID: 22385207.

320. Gujjar KR, Khadija H, Suleiman MO, Amith HV. Gingival health status of 2- to 15-year-old Benghazi children with type-I diabetes mellitus. Journal of dentistry for children (Chicago, Ill). 2011;78(2):96-101. Epub 2011/11/02. PubMed PMID: 22041114.

321. Haas AN, Wagner MC, Oppermann RV, Rosing CK, Albandar JM, Susin C. Risk factors for the progression of periodontal attachment loss: a 5-year population-based study in South Brazil. Journal of clinical periodontology. 2014;41(3):215-23. Epub 2013/12/07. doi: 10.1111/jcpe.12213. PubMed PMID: 24304168.

322. Hintao J, Teanpaisan R, Chongsuvivatwong V, Dahlen G, Rattarasarn C. Root surface and coronal caries in adults with type 2 diabetes mellitus. Community dentistry and oral epidemiology. 2007;35(4):302-9. Epub 2007/07/07. doi: 10.1111/j.1600-0528.2007.00325.x. PubMed PMID: 17615017.

323. Hintao J, Teanpaisan R, Chongsuvivatwong V, Ratarasan C, Dahlen G. The microbiological profiles of saliva, supragingival and subgingival plaque and dental caries in adults with and without type 2 diabetes mellitus. Oral microbiology and immunology. 2007;22(3):175-81. Epub 2007/05/10. doi: 10.1111/j.1399-302X.2007.00341.x. PubMed PMID: 17488443.

324. Hong M, Kim HY, Seok H, Yeo CD, Kim YS, Song JY, et al. Prevalence and risk factors of periodontitis among adults with or without diabetes mellitus. The Korean journal of internal medicine. 2016;31(5):910-9. Epub 2016/09/09. doi: 10.3904/kjim.2016.031. PubMed PMID: 27604799; PubMed Central PMCID: PMCPMC5016291.

325. Huang DL, Chan KC, Young BA. Poor oral health and quality of life in older U.S. adults with diabetes mellitus. Journal of the American Geriatrics Society. 2013;61(10):1782-8. Epub 2013/09/05. doi: 10.1111/jgs.12452. PubMed PMID: 24001058; PubMed Central PMCID: PMCPMC3855434.

326. Imran A, Parakh MK, Kumar SM, Nachiammai N, Sriram K. Periodontal health status and implication of periodic acid-Schiff diastase - a key in exfoliative cytology among diabetics mellitus patients: A case-control study. European journal of dentistry. 2016;10(4):475-9. Epub 2017/01/04. doi: 10.4103/1305-7456.195166. PubMed PMID: 28042261; PubMed Central PMCID: PMCPMC5166302.

327. Islas-Granillo H, Borges-Yanez SA, Lucas-Rincon SE, Medina-Solis CE, Casanova-Rosado AJ, Marquez-Corona ML, et al. Edentulism risk indicators among Mexican elders 60-

year-old and older. Archives of gerontology and geriatrics. 2011;53(3):258-62. Epub 2011/01/19. doi: 10.1016/j.archger.2010.12.014. PubMed PMID: 21242008.

328. Joshipura KJ, Munoz-Torres FJ, Dye BA, Leroux BG, Ramirez-Vick M, Perez CM. Longitudinal association between periodontitis and development of diabetes. Diabetes research and clinical practice. 2018;141:284-93. Epub 2018/04/22. doi: 10.1016/j.diabres.2018.04.028. PubMed PMID: 29679620; PubMed Central PMCID: PMCPMC6016543.

329. Kalakonda B, Koppolu P, Baroudi K, Mishra A. Periodontal Systemic Connections-Novel Associations-A Review of the Evidence with Implications for Medical Practitioners. International journal of health sciences. 2016;10(2):293-307. Epub 2016/04/23. PubMed PMID: 27103910; PubMed Central PMCID: PMCPMC4825901.

330. Lappin DF, Robertson D, Hodge P, Treagus D, Awang RA, Ramage G, et al. The Influence of Glycated Hemoglobin on the Cross Susceptibility Between Type 1 Diabetes Mellitus and Periodontal Disease. Journal of periodontology. 2015;86(11):1249-59. Epub 2015/08/08. doi: 10.1902/jop.2015.150149. PubMed PMID: 26252750.

331. Lee IC, Shieh TY, Yang YH, Tsai CC, Wang KH. Individuals' perception of oral health and its impact on the health-related quality of life. Journal of oral rehabilitation. 2007;34(2):79-87. Epub 2007/01/25. doi: 10.1111/j.1365-2842.2006.01694.x. PubMed PMID: 17244229.

332. Li C, Liu J, Tan L, Yu N, Lin L, Geng F, et al. The sociodemographic characteristics, periodontal health status, and subgingival microbiota of patients with chronic periodontitis and type 2 diabetes mellitus: a case-control study in a Chinese population. Journal of periodontology. 2013;84(8):1058-66. Epub 2012/10/04. doi: 10.1902/jop.2012.120282. PubMed PMID: 23030242.

333. Li S, Williams PL, Douglass CW. Development of a clinical guideline to predict undiagnosed diabetes in dental patients. Journal of the American Dental Association (1939). 2011;142(1):28-37. Epub 2011/01/05. PubMed PMID: 21193764.

334. Lorentz TC, Cota LO, Cortelli JR, Vargas AM, Costa FO. Prospective study of complier individuals under periodontal maintenance therapy: analysis of clinical periodontal parameters, risk predictors and the progression of periodontitis. Journal of clinical periodontology. 2009;36(1):58-67. Epub 2008/11/20. doi: 10.1111/j.1600-051X.2008.01342.x. PubMed PMID: 19017035.

335. Macek MD, Taylor GW, Tomar SL. Dental care visits among dentate adults with diabetes, United States, 2003. Journal of public health dentistry. 2008;68(2):102-10. Epub 2008/01/29. doi: 10.1111/j.1752-7325.2007.00064.x. PubMed PMID: 18221318.

336. Mayard-Pons ML, Rilliard F, Libersa JC, Musset AM, Farge P. Database analysis of a French type 2 diabetic population shows a specific age pattern of tooth extractions and correlates health care utilization. Journal of diabetes and its complications. 2015;29(8):993-7. Epub 2015/10/16. doi: 10.1016/j.jdiacomp.2015.09.007. PubMed PMID: 26463898.

337. Minaya-Sanchez M, Medina-Solis CE, Casanova-Rosado JF, Vallejos-Sanchez AA, Casanova-Rosado AJ, Marquez-Corona ML, et al. Prevalence of Functional Dentition in a Group of Mexican Adult Males. Puerto Rico health sciences journal. 2017;36(3):146-51. Epub 2017/09/16. PubMed PMID: 28915303.

338. Morita T, Yamazaki Y, Fujiharu C, Ishii T, Seto M, Nishinoue N, et al. Association Between the Duration of Periodontitis and Increased Cardiometabolic Risk Factors: A 9-Year

Cohort Study. Metabolic syndrome and related disorders. 2016;14(10):475-82. Epub 2016/10/16. doi: 10.1089/met.2016.0018. PubMed PMID: 27740886.

339. Motegi K, Nakano Y, Ueno T. Clinical studies on diabetes mellitus and diseases of the oral region. The Bulletin of Tokyo Medical and Dental University. 1975;22(3):243-7. Epub 1975/09/01. PubMed PMID: 1064491.

340. Natto ZS, Aladmawy M, Alasqah M, Papas A. Is there a relationship between periodontal disease and causes of death? A cross sectional study. Brazilian dental journal. 2015;26(1):33-8. Epub 2015/02/13. doi: 10.1590/0103-6440201300117. PubMed PMID: 25672381.

341. Nesse W, Linde A, Abbas F, Spijkervet FK, Dijkstra PU, de Brabander EC, et al. Dose-response relationship between periodontal inflamed surface area and HbA1c in type 2 diabetics. Journal of clinical periodontology. 2009;36(4):295-300. Epub 2009/05/12. doi: 10.1111/j.1600-051X.2009.01377.x. PubMed PMID: 19426175.

342. Nikbin A, Bayani M, Jenabian N, Khafri S, Motallebnejad M. Oral health-related quality of life in diabetic patients: comparison of the Persian version of Geriatric Oral Health Assessment Index and Oral Health Impact Profile: A descriptive-analytic study. Journal of diabetes and metabolic disorders. 2014;13(1):32. Epub 2014/02/06. doi: 10.1186/2251-6581-13-32. PubMed PMID: 24495383; PubMed Central PMCID: PMCPMC4015305.

343. Oh J, Gjelsvik A, Fuller D, Walsh E, Paine V, Leonard L. Less than optimal dental care among Rhode Island adults with diabetes: the need to assure oral health care for all adults with diabetes. Medicine and health, Rhode Island. 2012;95(3):91-3. Epub 2012/04/27. PubMed PMID: 22533227.

344. Oliveira EJP, Rocha VFB, Nogueira DA, Pereira AA. Quality of life and oral health among hypertensive and diabetic people in a Brazilian Southeastern city. Ciencia & saude coletiva. 2018;23(3):763-72. Epub 2018/03/15. doi: 10.1590/1413-81232018233.00752016. PubMed PMID: 29538557.

345. Preferansow E, Golebiewska M, Kulikowska-Bielaczyc E, Gorska M. The assessment of periodontium in patients with uncontrolled diabetes. Advances in medical sciences. 2006;51 Suppl 1:170-2. Epub 2007/04/27. PubMed PMID: 17458085.

346. Puttaswamy KA, Puttabudhi JH, Raju S. Correlation between Salivary Glucose and Blood Glucose and the Implications of Salivary Factors on the Oral Health Status in Type 2 Diabetes Mellitus Patients. Journal of International Society of Preventive & Community Dentistry. 2017;7(1):28-33. Epub 2017/03/21. doi: 10.4103/2231-0762.200703. PubMed PMID: 28316946; PubMed Central PMCID: PMCPMC5343680.

347. Saito M, Shimazaki Y, Nonoyama T, Tadokoro Y. Association between dental visits for periodontal treatment and type 2 diabetes mellitus in an elderly Japanese cohort. Journal of clinical periodontology. 2017;44(11):1133-9. Epub 2017/08/25. doi: 10.1111/jcpe.12804. PubMed PMID: 28836290.

348. Sakalauskiene J, Kubilius R, Gleiznys A, Vitkauskiene A, Ivanauskiene E, Saferis V. Relationship of clinical and microbiological variables in patients with type 1 diabetes mellitus and periodontitis. Medical science monitor : international medical journal of experimental and clinical research. 2014;20:1871-7. Epub 2014/10/09. doi: 10.12659/msm.890879. PubMed PMID: 25294115; PubMed Central PMCID: PMCPMC4199460.

349. Sakallioglu EE, Lutfioglu M, Sakallioglu U, Diraman E, Keskiner I. Fluid dynamics of gingiva in diabetic and systemically healthy periodontitis patients. Archives of oral biology.

2008;53(7):646-51. Epub 2008/02/19. doi: 10.1016/j.archoralbio.2007.12.013. PubMed PMID: 18281015.

350. Schulze A, Busse M. Gender Differences in Periodontal Status and Oral Hygiene of Non-Diabetic and Type 2 Diabetic Patients. The open dentistry journal. 2016;10:287-97. Epub 2016/06/28. doi: 10.2174/1874210601610010287. PubMed PMID: 27347232; PubMed Central PMCID: PMCPMC4901196.

351. Shearer DM, Thomson WM, Broadbent JM, Mann J, Poulton R. Periodontitis is not associated with metabolic risk during the fourth decade of life. Journal of clinical periodontology. 2017;44(1):22-30. Epub 2016/10/27. doi: 10.1111/jcpe.12641. PubMed PMID: 27783846.

352. Susanto H, Nesse W, Dijkstra PU, Hoedemaker E, van Reenen YH, Agustina D, et al. Periodontal inflamed surface area and C-reactive protein as predictors of HbA1c: a study in Indonesia. Clinical oral investigations. 2012;16(4):1237-42. Epub 2011/10/21. doi: 10.1007/s00784-011-0621-0. PubMed PMID: 22012468; PubMed Central PMCID: PMCPMC3400038.

353. Taylor GW, Burt BA, Becker MP, Genco RJ, Shlossman M. Glycemic control and alveolar bone loss progression in type 2 diabetes. Annals of periodontology. 1998;3(1):30-9. Epub 1998/09/02. doi: 10.1902/annals.1998.3.1.30. PubMed PMID: 9722688.

354. Taylor GW, Burt BA, Becker MP, Genco RJ, Shlossman M, Knowler WC, et al. Severe Periodontitis and Risk for Poor Glycemic Control in Patients with Non-Insulin-Dependent Diabetes Mellitus. Journal of periodontology. 1996;67 Suppl 10S:1085-93. Epub 1996/10/01. doi: 10.1902/jop.1996.67.10s.1085. PubMed PMID: 29539790.

355. Taylor GW, Burt BA, Becker MP, Genco RJ, Shlossman M, Knowler WC, et al. Non-insulin dependent diabetes mellitus and alveolar bone loss progression over 2 years. Journal of periodontology. 1998;69(1):76-83. Epub 1998/04/04. doi: 10.1902/jop.1998.69.1.76. PubMed PMID: 9527565.

356. Williams RC, Jr., Mahan CJ. Periodontal disease and diabetes in young adults. Journal of the American Medical Association. 1960;172:776-8. Epub 1960/02/20. PubMed PMID: 13844923.

357. Zilinskas J, Kubilius R, Zekonis G, Zekonis J. Total antioxidant capacity of venous blood, blood plasma, and serum of patients with periodontitis, and the effect of Traumeel S on these characteristics. Medicina (Kaunas, Lithuania). 2011;47(4):193-9. Epub 2011/08/11. PubMed PMID: 21829050.

358. Albright JW, Woo PH, Ji SQ, Sun BG, Lang K, Albright JF. Synergism between Obesity and Poor Oral Health Associated with Diabetes in an Elderly Human Population. Southeast Asian Journal of Tropical Medicine and Public Health. 2013;44(2):318-31. PubMed PMID: WOS:000327171400022.

359. Azogui-Levy S, Dray-Spira R, Attal S, Hartemann A, Anagnostou F, Azerad J. Factors associated with oral health-related quality of life in patients with diabetes. Australian Dental Journal. 2018;63(2):163-9. doi: 10.1111/adj.12577. PubMed PMID: WOS:000434056100005.

360. D'Aiuto F, Sabbah W, Netuveli G, Donos N, Hingorani AD, Deanfield J, et al. Association of the metabolic syndrome with severe periodontitis in a large US population-based survey. Journal of Clinical Endocrinology & Metabolism. 2008;93(10):3989-94. doi: 10.1210/jc.2007-2522. PubMed PMID: WOS:000259903700047.

361. Elias-Boneta AR, Toro MJ, Rivas-Tumanyan S, Rajendra-Santoch AB, Brache M, Collins JR. Prevalence, Severity, and Risk Factors of Gingival Inflammation in Caribbean Adults: A Multi-City, Cross-Sectional Study. Puerto Rico Health Sciences Journal. 2018;37(2):115-23. PubMed PMID: WOS:000434974900008.

362. Gopalakrishnan D, Miller PD, Mahuli AV, Sangamithra S, Phantumvanit P, Buranawat B. Evaluation of periodontally diseased molars in diabetics with Miller-McEntire Periodontal Prognostic Index. Journal of Indian Society of Periodontology. 2018;22(5):401-5. Epub 2018/09/14. doi: 10.4103/jisp.jisp_271_18. PubMed PMID: 30210188; PubMed Central PMCID: PMCPMC6128126.

363. Haheim LL, Ronningen KS, Enersen M, Olsen I. The Predictive Role of Tooth Extractions, Oral Infections, and hs-C-Reactive Protein for Mortality in Individuals with and without Diabetes: A Prospective Cohort Study of a 12 1/2-Year Follow-Up. Journal of Diabetes Research. 2017. doi: 10.1155/2017/9590740. PubMed PMID: WOS:000403961300001.

364. Huang JC, Peng YS, Fan JY, Jane SW, Tu LT, Chang CC, et al. Factors associated with numbers of remaining teeth among type 2 diabetes: a cross-sectional study. Journal of Clinical Nursing. 2013;22(13-14):1926-32. doi: 10.1111/jocn.12225. PubMed PMID: WOS:000320138300015.

365. Lee PH, McGrath CPJ, Kong AYC, Lam TH. Self-report poor oral health and chronic diseases: the Hong Kong FAMILY project. Community Dentistry and Oral Epidemiology. 2013;41(5):451-8. doi: 10.1111/cdoe.12037. PubMed PMID: WOS:000325001800008.

366. Nabee Z, Jeewon R, Pugo-Gunsam P. Oral dysbacteriosis in type 2 diabetes and its role in the progression to cardiovascular disease. African Health Sciences. 2017;17(4):1082-91. doi: 10.4314/ahs.v17i4.16. PubMed PMID: WOS:000422885100016.

367. Popescu D, Gheorghe D, Puiu I, Rica AM. Odontal-Periodontal Changes in Patients with Type 1 Diabetes. Current health sciences journal. 2017;43(4):330-4. Epub 2017/10/01. doi: 10.12865/chsj.43.04.07. PubMed PMID: 30595898; PubMed Central PMCID: PMCPMC6286457.

368. Sabharwal A, Ganley K, Miecznikowski JC, Haase EM, Barnes V, Scannapieco FA. The salivary microbiome of diabetic and non-diabetic adults with periodontal disease. Journal of periodontology. 2019;90(1):26-34. Epub 2018/07/13. doi: 10.1002/jper.18-0167. PubMed PMID: 29999529.

369. Albuquerque BN, Cota LOM, Lorentz TCM, Costa FO. Periodontal Maintenance Therapy in a Public University: A Six-Year Prospective Study. J Int Acad Periodontol. 2018;20(4):143-52. Epub 2019/09/16. PubMed PMID: 31522149.

370. Byon MJ, Kim SY, Kim JS, Kim HN, Kim JB. Association of Periodontitis with Atherosclerotic Cardiovascular Diseases: A Nationwide Population-based Retrospective Matched Cohort Study. Int J Environ Res Public Health. 2020;17(19). Epub 2020/10/07. doi: 10.3390/ijerph17197261. PubMed PMID: 33020434; PubMed Central PMCID: PMCPMC7578974.

371. Gaio EJ, Haas AN, Rösing CK, Oppermann RV, Albandar JM, Susin C. Effect of obesity on periodontal attachment loss progression: a 5-year population-based prospective study. J Clin Periodontol. 2016;43(7):557-65. Epub 2016/03/13. doi: 10.1111/jcpe.12544. PubMed PMID: 26970086.

372. Malthaner SC, Moore S, Mills M, Saad R, Sabatini R, Takacs V, et al. Investigation of the association between angiographically defined coronary artery disease and periodontal disease. J Periodontol. 2002;73(10):1169-76. Epub 2002/11/06. doi: 10.1902/jop.2002.73.10.1169. PubMed PMID: 12416775.

373. Zhao D, Zhen Z, Pelekos G, Yiu KH, Jinl LJ. Periodontal disease increases the risk for onset of systemic comorbidities in dental hospital attendees: An 18-year retrospective cohort study. Journal of Periodontology. 2019;90(3):225-33. doi: 10.1002/JPER.18-0224. PubMed PMID: WOS:000459824400001.

374. Bole C, Wactawski-Wende J, Hovey KM, Genco RJ, Hausmann E. Clinical and community risk models of incident tooth loss in postmenopausal women from the Buffalo Osteo Perio Study. Community dentistry and oral epidemiology. 2010;38(6):487-97. Epub 2010/07/20. doi: 10.1111/j.1600-0528.2010.00555.x. PubMed PMID: 20636416; PubMed Central PMCID: PMCPMC2975786.

375. Demmer RT, Holtfreter B, Desvarieux M, Jacobs DR, Jr., Kerner W, Nauck M, et al. The influence of type 1 and type 2 diabetes on periodontal disease progression: prospective results from the Study of Health in Pomerania (SHIP). Diabetes care. 2012;35(10):2036-42. Epub 2012/08/03. doi: 10.2337/dc11-2453. PubMed PMID: 22855731; PubMed Central PMCID: PMCPMC3447825.

376. Yoo JJ, Kim DW, Kim MY, Kim YT, Yoon JH. The effect of diabetes on tooth loss due to periodontal disease: A nationwide population-based cohort study in south korea. Journal of periodontology. 2018. Epub 2018/12/15. doi: 10.1002/jper.18-0480. PubMed PMID: 30548930.

377. Akyuz S, Oktay C. The relationship between periodontitis and tooth decay in juvenile diabetes mellitus cases and in healthy children. Journal of Marmara University Dental Faculty. 1990;1(1):58-65. Epub 1990/09/01. PubMed PMID: 2129918.

378. Albrecht M, Banoczy J, Baranyi E, Tamas G, Jr., Szalay J, Egyed J, et al. Studies of dental and oral changes of pregnant diabetic women. Acta diabetologica latina. 1987;24(1):1-7. Epub 1987/01/01. PubMed PMID: 3497504.

379. Bernick SM, Cohen DW, Baker L, Laster L. Dental disease in children with diabetes mellitus. Journal of periodontology. 1975;46(4):241-5. Epub 1975/04/01. doi: 10.1902/jop.1975.46.4.241. PubMed PMID: 1055217.

380. Carneiro VL, Fraiz FC, Ferreira Fde M, Pintarelli TP, Oliveira AC, Boguszewski MC. The influence of glycemic control on the oral health of children and adolescents with diabetes mellitus type 1. Archives of endocrinology and metabolism. 2015;59(6):535-40. Epub 2015/12/18. doi: 10.1590/2359-3997000000117. PubMed PMID: 26677088.

381. Cianciola LJ, Park BH, Bruck E, Mosovich L, Genco RJ. Prevalence of periodontal disease in insulin-dependent diabetes mellitus (juvenile diabetes). Journal of the American Dental Association (1939). 1982;104(5):653-60. Epub 1982/05/01. PubMed PMID: 7042797.

382. Coelho A, Carneiro AS, Pereira VF, Paula AP, Macedo AP, Carrilho EVP. Oral Health of Portuguese Children with Type 1 Diabetes: A Multiparametric Evaluation. The Journal of clinical pediatric dentistry. 2018;42(3):231-5. Epub 2018/04/27. doi: 10.17796/1053-4628-42.3.12. PubMed PMID: 29698136.

383. Dakovic D, Pavlovic MD. Periodontal disease in children and adolescents with type 1 diabetes in Serbia. Journal of periodontology. 2008;79(6):987-92. Epub 2008/06/07. doi: 10.1902/jop.2008.070549. PubMed PMID: 18533774.

384. Diaz R. Diabetes: Diabetes mellitus and periodontal disease in youth. Nature reviews Endocrinology. 2011;7(6):313. Epub 2011/05/06. doi: 10.1038/nrendo.2011.64. PubMed PMID: 21544052.

385. Diaz Rosas CY, Cardenas Vargas E, Castaneda-Delgado JE, Aguilera-Galaviz LA, Aceves Medina MC. Dental, periodontal and salivary conditions in diabetic children associated with metabolic control variables and nutritional plan adherence. European journal of paediatric dentistry : official journal of European Academy of Paediatric Dentistry. 2018;19(2):119-26. Epub 2018/05/24. doi: 10.23804/ejpd.2018.19.02.05. PubMed PMID: 29790775.

386. Duque C, Joao MF, Camargo GA, Teixeira GS, Machado TS, Azevedo RS, et al. Microbiological, lipid and immunological profiles in children with gingivitis and type 1 diabetes mellitus. Journal of applied oral science : revista FOB. 2017;25(2):217-26. Epub 2017/04/14. doi: 10.1590/1678-77572016-0196. PubMed PMID: 28403363; PubMed Central PMCID: PMCPMC5393543.

387. Firatli E, Yilmaz O, Onan U. The relationship between clinical attachment loss and the duration of insulin-dependent diabetes mellitus (IDDM) in children and adolescents. Journal of clinical periodontology. 1996;23(4):362-6. Epub 1996/04/01. PubMed PMID: 8739168.

388. Giuca MR, Pasini M, Giuca G, Caruso S, Necozione S, Gatto R. Investigation of periodontal status in type 1 diabetic adolescents. European journal of paediatric dentistry : official journal of European Academy of Paediatric Dentistry. 2015;16(4):319-23. Epub 2015/12/08. PubMed PMID: 26637258.

389. Harrison R, Bowen WH. Periodontal health, dental caries, and metabolic control in insulin-dependent diabetic children and adolescents. Pediatric dentistry. 1987;9(4):283-6. Epub 1987/12/01. PubMed PMID: 3507646.

390. Ismail AF, McGrath CP, Yiu CKY. Oral health status of children with type 1 diabetes: a comparative study. Journal of pediatric endocrinology & metabolism : JPEM. 2017;30(11):1155-9. Epub 2017/10/11. doi: 10.1515/jpem-2017-0053. PubMed PMID: 28988224.

391. Iughetti L, Marino R, Bertolani MF, Bernasconi S. Oral health in children and adolescents with IDDM--a review. Journal of pediatric endocrinology & metabolism : JPEM. 1999;12(5 Suppl 2):603-10. Epub 2000/06/15. PubMed PMID: 10854189.

392. Jindal A, Parihar AS, Sood M, Singh P, Singh N. Relationship between Severity of Periodontal Disease and Control of Diabetes (Glycated Hemoglobin) in Patients with Type 1 Diabetes Mellitus. Journal of international oral health : JIOH. 2015;7(Suppl 2):17-20. Epub 2015/12/17. PubMed PMID: 26668475; PubMed Central PMCID: PMCPMC4672852.

393. Karjalainen KM, Knuuttila ML. The onset of diabetes and poor metabolic control increases gingival bleeding in children and adolescents with insulin-dependent diabetes mellitus. Journal of clinical periodontology. 1996;23(12):1060-7. Epub 1996/12/01. PubMed PMID: 8997648.

394. Kasaj A, Zafiropoulos GG, Tekyatan H, Pistorius A, Willershausen B. Periodontal disease status of pregnant women with diabetes mellitus. Collegium antropologicum. 2008;32(1):115-8. Epub 2008/05/23. PubMed PMID: 18494196.

395. Lalla E, Cheng B, Lal S, Kaplan S, Softness B, Greenberg E, et al. Diabetes-related parameters and periodontal conditions in children. Journal of periodontal research.

2007;42(4):345-9. Epub 2007/06/15. doi: 10.1111/j.1600-0765.2006.00955.x. PubMed PMID: 17559632.

396. Lalla E, Cheng B, Lal S, Kaplan S, Softness B, Greenberg E, et al. Diabetes mellitus promotes periodontal destruction in children. Journal of clinical periodontology. 2007;34(4):294-8. Epub 2007/03/24. doi: 10.1111/j.1600-051X.2007.01054.x. PubMed PMID: 17378885.

397. Lifshitz F, Casavalle PL, Bordoni N, Rodriguez PN, Friedman SM. Oral Health in Children with Obesity or Diabetes Mellitus. Pediatric endocrinology reviews : PER. 2016;14(2):159-67. Epub 2017/05/17. doi: 10.17458/PER.2016.LCB.Oralhealth. PubMed PMID: 28508609.

398. Luczaj-Cepowicz E, Marczuk-Kolada G, Waszkiel D. Evaluation of periodontal status in young patients with insulin-dependent diabetes mellitus (type 1). Advances in medical sciences. 2006;51 Suppl 1:134-7. Epub 2007/04/27. PubMed PMID: 17458077.

399. Merchant AT, Jethwani M, Choi YH, Morrato EH, Liese AD, Mayer-Davis E. Associations between periodontal disease and selected risk factors of early complications among youth with type 1 and type 2 diabetes: a pilot study. Pediatric diabetes. 2011;12(6):529-35. Epub 2011/03/12. doi: 10.1111/j.1399-5448.2010.00736.x. PubMed PMID: 21392193.

400. Nishimura F, Takahashi K, Kurihara M, Takashiba S, Murayama Y. Periodontal disease as a complication of diabetes mellitus. Annals of periodontology. 1998;3(1):20-9. Epub 1998/09/02. doi: 10.1902/annals.1998.3.1.20. PubMed PMID: 9722687.

401. Novaes AB, Jr., Pereira AL, de Moraes N, Novaes AB. Manifestations of insulin-dependent diabetes mellitus in the periodontium of young Brazilian patients. Journal of periodontology. 1991;62(2):116-22. Epub 1991/02/01. doi: 10.1902/jop.1991.62.2.116. PubMed PMID: 2027059.

402. Oh TJ, Eber R, Wang HL. Periodontal diseases in the child and adolescent. Journal of clinical periodontology. 2002;29(5):400-10. Epub 2002/06/13. PubMed PMID: 12060422.

403. Orbak R, Simsek S, Orbak Z, Kavrut F, Colak M. The influence of type-1 diabetes mellitus on dentition and oral health in children and adolescents. Yonsei medical journal. 2008;49(3):357-65. Epub 2008/06/27. doi: 10.3349/ymj.2008.49.3.357. PubMed PMID: 18581583; PubMed Central PMCID: PMCPMC2615350.

404. Pinson M, Hoffman WH, Garnick JJ, Litaker MS. Periodontal disease and type I diabetes mellitus in children and adolescents. Journal of clinical periodontology. 1995;22(2):118-23. Epub 1995/02/01. PubMed PMID: 7775667.

405. Rafatjou R, Razavi Z, Tayebi S, Khalili M, Farhadian M. Dental Health Status and Hygiene in Children and Adolescents with Type 1 Diabetes Mellitus. Journal of research in health sciences. 2016;16(3):122-6. Epub 2016/11/15. PubMed PMID: 27840339.

406. Rosenthal IM, Abrams H, Kopczyk A. The relationship of inflammatory periodontal disease to diabetic status in insulin-dependent diabetes mellitus patients. Journal of clinical periodontology. 1988;15(7):425-9. Epub 1988/08/01. PubMed PMID: 3141483.

407. Sadeghi R, Taleghani F, Mohammadi S, Zohri Z. The Effect of Diabetes Mellitus Type I on Periodontal and Dental Status. Journal of clinical and diagnostic research : JCDR. 2017;11(7):Zc14-zc7. Epub 2017/09/13. doi: 10.7860/jcdr/2017/25742.10153. PubMed PMID: 28893034; PubMed Central PMCID: PMCPMC5583944.

408. Saes Busato IM, Bittencourt MS, Machado MA, Gregio AM, Azevedo-Alanis LR. Association between metabolic control and oral health in adolescents with type 1 diabetes mellitus. Oral surgery, oral medicine, oral pathology, oral radiology, and endodontics. 2010;109(3):e51-6. Epub 2010/03/12. doi: 10.1016/j.tripleo.2009.10.037. PubMed PMID: 20219586.

409. Saminsky M. Periodontal Disease and Dental Caries among children and Adolescents Suffering from Endocrine Disorders - A Literature Review. Pediatric endocrinology reviews : PER. 2017;15(2):165-72. Epub 2018/01/03. doi: 10.17458/per.vol15.2017.sam.periodontaldiseasedental. PubMed PMID: 29292628.

410. Sandholm L, Swanljung O, Rytomaa I, Kaprio EA, Maenpaa J. Periodontal status of Finnish adolescents with insulin-dependent diabetes mellitus. Journal of clinical periodontology. 1989;16(10):617-20. Epub 1989/11/01. PubMed PMID: 2613930.

411. Sbordone L, Ramaglia L, Barone A, Ciaglia RN, Iacono VJ. Periodontal status and subgingival microbiota of insulin-dependent juvenile diabetics: a 3-year longitudinal study. Journal of periodontology. 1998;69(2):120-8. Epub 1998/04/04. doi: 10.1902/jop.1998.69.2.120. PubMed PMID: 9526910.

412. Sbordone L, Ramaglia L, Barone A, Ciaglia RN, Tenore A, Iacono VJ. Periodontal status and selected cultivable anaerobic microflora of insulin-dependent juvenile diabetics. Journal of periodontology. 1995;66(6):452-61. Epub 1995/06/01. doi: 10.1902/jop.1995.66.6.452. PubMed PMID: 7562335.

413. Siudikiene J, Maciulskiene V, Dobrovolskiene R, Nedzelskiene I. Oral hygiene in children with type I diabetes mellitus. Stomatologija. 2005;7(1):24-7. Epub 2005/10/29. PubMed PMID: 16254474.

414. Sjodin B, Edblad E, Sondell K, Dahlen G. Minor manifestations of periodontal diseases in young adults with type 1 diabetes mellitus. Periodontal and microbiological findings. Acta odontologica Scandinavica. 2012;70(6):589-96. Epub 2012/03/01. doi: 10.3109/00016357.2011.640288. PubMed PMID: 22364291.

415. Swapna LA, Koppolu P, Prince J. Oral health in diabetic and nondiabetic patients with chronic kidney disease. Saudi journal of kidney diseases and transplantation : an official publication of the Saudi Center for Organ Transplantation, Saudi Arabia. 2017;28(5):1099-105. Epub 2017/09/25. doi: 10.4103/1319-2442.215123. PubMed PMID: 28937069.

416. Sznajder N, Carraro JJ, Rugna S, Sereday M. Periodontal findings in diabetic and nondiabetic patients. Journal of periodontology. 1978;49(9):445-8. Epub 1978/09/01. doi: 10.1902/jop.1978.49.9.445. PubMed PMID: 281490.

417. Wooton AK, Melchior LM, Coan LL, Reddington AR. Periodontal disease in children with type 2 diabetes mellitus. The Nurse practitioner. 2018;43(2):30-5. Epub 2018/01/09. doi: 10.1097/01.npr.0000529666.08823.01. PubMed PMID: 29309381.

418. Alkhabbaz A, Abdulrasoul M. Periodontal Disease in Children with Type 1 Diabetes Mellitus. Diabetes Technology & Therapeutics. 2013;15:A27-A. PubMed PMID: WOS:000315467200074.

419. Cioloca DP, Ursarescu I, Martu A, Toma V, Surdu A, Botnariu G, et al. Systemic and Periodontal Inflammatory Burden in Children and Teenagers with Diabetes, and Clinical Correlations. Medical-Surgical Journal-Revista Medico-Chirurgicala. 2015;119(3):896-902. PubMed PMID: WOS:000409111700044.

420. del Valle LML, Ocasio-Lopez C. Comparing the Oral Health Status of Diabetic and Non-Diabetic Children from Puerto Rico: a Case-control Pilot Study. Puerto Rico Health Sciences Journal. 2011;30(3):123-7. PubMed PMID: WOS:000294547900004.

421. Denisse DM, Nelly MF, Enrique CC, Enrique G, Rosa RR, Omar TM, et al. Relationship between Periodontal Disease and Type 1 Diabetes in Adolescents. Annals of Medical and Health Sciences Research. 2017;7(6):350-4. PubMed PMID: WOS:000428751500001.

422. Depommereau V, Pare C, Bordais P, Robert JJ. Insulin-Dependent Diabetes-Mellitus and Periodontal-Disease in Young-Patients. Annales De Pediatrie. 1991;38(4):235-9. PubMed PMID: WOS:A1991FM36600007.

423. Ehsanpour MG, Sodeif F. Evaluating the Effect of Type I Diabetics on the Prevalence of Periodontal Diseases in Children. Annals of Dental Specialty. 2018;6(3):232-5. PubMed PMID: WOS:000447455900001.

424. Leitao ACD, dos Santos EUD, de Souza PRE, Cimoes R. Periodontal assessment in children and adolescents with type 1 diabetes mellitus: a case-control study. Revista Portuguesa De Estomatologia Medicina Dentaria E Cirurgia Maxilofacial. 2017;58(4):225-30. doi: 10.24873/j.rpemd.2017.02.213. PubMed PMID: WOS:000442146800006.

425. Pieper K, Schwartz H. Caries Frequency, Periodontal Condition and Oral Hygiene in Children with Insulin-Controlled Diabetes-Mellitus. Caries Research. 1983;17(2):178-9. PubMed PMID: WOS:A1983QC12000072.

426. Pinson M, Hoffman WH, Garnick JJ, Litaker MS. Periodontal-Disease and Type-1 Diabetes-Mellitus in Children and Adolescents. Journal of Clinical Periodontology. 1995;22(2):118-23. PubMed PMID: WOS:A1995QL21500004.

427. Vidya K, Shetty P, Anandakrishna L. Oral health and glycosylated hemoglobin among type 1 diabetes children in South India. Journal of the Indian Society of Pedodontics and Preventive Dentistry. 2018;36(1):38-42. Epub 2018/04/03. doi: 10.4103/jisppd.jisppd_330_16. PubMed PMID: 29607837.

428. Nelson RG, Shlossman M, Budding LM, Pettitt DJ, Saad MF, Genco RJ, et al. Periodontal disease and NIDDM in Pima Indians. Diabetes care. 1990;13(8):836-40. Epub 1990/08/01. PubMed PMID: 2209317.

429. Abduljabbar T, Al-Sahaly F, Al-Kathami M, Afzal S, Vohra F. Comparison of periodontal and peri-implant inflammatory parameters among patients with prediabetes, type 2 diabetes mellitus and non-diabetic controls. Acta odontologica Scandinavica. 2017;75(5):319-24. Epub 2017/03/23. doi: 10.1080/00016357.2017.1303848. PubMed PMID: 28325134.

430. Al-Shammari KF, Al-Ansari JM, Moussa NM, Ben-Nakhi A, Al-Arouj M, Wang HL. Association of periodontal disease severity with diabetes duration and diabetic complications in patients with type 1 diabetes mellitus. Journal of the International Academy of Periodontology. 2006;8(4):109-14. Epub 2006/10/18. PubMed PMID: 17042166.

431. Aldridge JP, Lester V, Watts TL, Collins A, Viberti G, Wilson RF. Single-blind studies of the effects of improved periodontal health on metabolic control in type 1 diabetes mellitus. Journal of clinical periodontology. 1995;22(4):271-5. Epub 1995/04/01. PubMed PMID: 7622632.

432. Andriankaja OM, Munoz-Torres FJ, Vivaldi-Oliver J, Leroux BG, Campos M, Joshipura K, et al. Insulin resistance predicts the risk of gingival/periodontal inflammation. Journal of periodontology. 2018;89(5):549-57. Epub 2018/03/10. doi: 10.1002/jper.17-0384. PubMed PMID: 29520795; PubMed Central PMCID: PMCPMC5984160.

433. Auyeung L, Wang PW, Lin RT, Hsieh CJ, Lee PY, Zhuang RY, et al. Evaluation of periodontal status and effectiveness of non-surgical treatment in patients with type 2 diabetes mellitus in Taiwan for a 1-year period. Journal of periodontology. 2012;83(5):621-8. Epub 2011/06/23. doi: 10.1902/jop.2011.110133. PubMed PMID: 21692625.

434. Bandyopadhyay D, Marlow NM, Fernandes JK, Leite RS. Periodontal disease progression and glycaemic control among Gullah African Americans with type-2 diabetes. Journal of clinical periodontology. 2010;37(6):501-9. Epub 2010/05/29. doi: 10.1111/j.1600-051X.2010.01564.x. PubMed PMID: 20507373; PubMed Central PMCID: PMCPMC2891073.

435. Buzinin SM, Alabsi AM, Tan AT, Vincent-Chong VK, Swaminathan D. Effects of nonsurgical periodontal therapy on clinical response, microbiological profile, and glycemic control in Malaysian subjects with type 1 diabetes. TheScientificWorldJournal. 2014;2014:232535. Epub 2014/08/26. doi: 10.1155/2014/232535. PubMed PMID: 25147841; PubMed Central PMCID: PMCPMC4132400.

436. Calabrese N, D'Aiuto F, Calabrese A, Patel K, Calabrese G, Massi-Benedetti M. Effects of periodontal therapy on glucose management in people with diabetes mellitus. Diabetes & metabolism. 2011;37(5):456-9. Epub 2011/07/16. doi: 10.1016/j.diabet.2011.05.004. PubMed PMID: 21757386.

437. Camargo GA, Lima Mde A, Fortes TV, de Souza CS, de Jesus AM, de Almeida RP. Effect of periodontal therapy on metabolic control and levels of IL-6 in the gingival crevicular fluid in type 2 diabetes mellitus. Indian journal of dental research : official publication of Indian Society for Dental Research. 2013;24(1):110-6. Epub 2013/07/16. doi: 10.4103/0970-9290.114953. PubMed PMID: 23852243.

438. Cleary TJ, Hutton JE. An assessment of the association between functional edentulism, obesity, and NIDDM. Diabetes care. 1995;18(7):1007-9. Epub 1995/07/01. PubMed PMID: 7555531.

439. Corbella S, Francetti L, Taschieri S, De Siena F, Fabbro MD. Effect of periodontal treatment on glycemic control of patients with diabetes: A systematic review and meta-analysis. Journal of diabetes investigation. 2013;4(5):502-9. Epub 2014/05/21. doi: 10.1111/jdi.12088. PubMed PMID: 24843701; PubMed Central PMCID: PMCPMC4025114.

440. Costa FO, Miranda Cota LO, Pereira Lages EJ, Soares Dutra Oliveira AM, Dutra Oliveira PA, Cyrino RM, et al. Progression of periodontitis and tooth loss associated with glycemic control in individuals undergoing periodontal maintenance therapy: a 5-year follow-up study. Journal of periodontology. 2013;84(5):595-605. Epub 2012/07/10. doi: 10.1902/jop.2012.120255. PubMed PMID: 22769441.

441. Dag A, Firat ET, Arikan S, Kadiroglu AK, Kaplan A. The effect of periodontal therapy on serum TNF-alpha and HbA1c levels in type 2 diabetic patients. Australian dental journal. 2009;54(1):17-22. Epub 2009/02/21. doi: 10.1111/j.1834-7819.2008.01083.x. PubMed PMID: 19228128.

442. Esteves Lima RP, Cota LO, Silva TA, Cortelli SC, Cortelli JR, Costa FO. Periodontitis and type 2 diabetes among women with previous gestational diabetes: epidemiological and immunological aspects in a follow-up of three years. Journal of applied oral science : revista

FOB. 2017;25(2):130-9. Epub 2017/04/14. doi: 10.1590/1678-77572016-0367. PubMed PMID: 28403353; PubMed Central PMCID: PMCPMC5393533.

443. Fardal O, Fardal P, Persson GR. Periodontal and general health in long-term periodontal maintenance patients treated in a Norwegian private practice: a descriptive report from a compliant and partially compliant survivor population. Journal of periodontology. 2013;84(10):1374-81. Epub 2012/11/10. doi: 10.1902/jop.2012.120416. PubMed PMID: 23136948.

444. Furuichi Y, Shimotsu A, Ito H, Namariyama Y, Yotsumoto Y, Hino Y, et al. Associations of periodontal status with general health conditions and serum antibody titers for Porphyromonas gingivalis and Actinobacillus actinomycetemcomitans. Journal of periodontology. 2003;74(10):1491-7. Epub 2003/12/05. doi: 10.1902/jop.2003.74.10.1491. PubMed PMID: 14653396.

445. Holmer H, Widen C, Wallin Bengtsson V, Coleman M, Wohlfart B, Steen S, et al. Improved General and Oral Health in Diabetic Patients by an Okinawan-Based Nordic Diet: A Pilot Study. International journal of molecular sciences. 2018;19(7). Epub 2018/07/05. doi: 10.3390/ijms19071949. PubMed PMID: 29970834; PubMed Central PMCID: PMCPMC6073471.

446. Kapellas K, Do LG, Bartold PM, Skilton MR, Maple-Brown LJ, O'Dea K, et al. Effects of full-mouth scaling on the periodontal health of Indigenous Australians: a randomized controlled trial. Journal of clinical periodontology. 2013;40(11):1016-24. Epub 2013/09/03. doi: 10.1111/jcpe.12152. PubMed PMID: 23992525.

447. Marlow NM, Slate EH, Bandyopadhyay D, Fernandes JK, Leite RS. Health insurance status is associated with periodontal disease progression among Gullah African-Americans with type 2 diabetes mellitus. Journal of public health dentistry. 2011;71(2):143-51. Epub 2011/07/21. PubMed PMID: 21774138; PubMed Central PMCID: PMCPMC4234040.

448. Maupome G, Gullion CM, White BA, Wyatt CC, Williams PM. Oral disorders and chronic systemic diseases in very old adults living in institutions. Special care in dentistry : official publication of the American Association of Hospital Dentists, the Academy of Dentistry for the Handicapped, and the American Society for Geriatric Dentistry. 2003;23(6):199-208. Epub 2004/04/17. PubMed PMID: 15085956.

449. Moeintaghavi A, Arab HR, Bozorgnia Y, Kianoush K, Alizadeh M. Non-surgical periodontal therapy affects metabolic control in diabetics: a randomized controlled clinical trial. Australian dental journal. 2012;57(1):31-7. Epub 2012/03/01. doi: 10.1111/j.1834-7819.2011.01652.x. PubMed PMID: 22369555.

450. Moore PA, Weyant RJ, Mongelluzzo MB, Myers DE, Rossie K, Guggenheimer J, et al. Type 1 diabetes mellitus and oral health: assessment of tooth loss and edentulism. Journal of public health dentistry. 1998;58(2):135-42. Epub 1998/09/08. PubMed PMID: 9729758.

451. Perayil J, Suresh N, Fenol A, Vyloppillil R, Bhaskar A, Menon S. Comparison of glycated hemoglobin levels in individuals without diabetes and with and without periodontitis before and after non-surgical periodontal therapy. Journal of periodontology. 2014;85(12):1658-66. Epub 2014/06/27. doi: 10.1902/jop.2014.130661. PubMed PMID: 24968250.

452. Perez-Losada FL, Jane-Salas E, Sabater-Recolons MM, Estrugo-Devesa A, Segura-Egea JJ, Lopez-Lopez J. Correlation between periodontal disease management and metabolic control of type 2 diabetes mellitus. A systematic literature review. Medicina oral,

patologia oral y cirugia bucal. 2016;21(4):e440-6. Epub 2016/02/02. PubMed PMID: 26827070; PubMed Central PMCID: PMCPMC4920457.

453. Persson RE, Persson GR, Kiyak HA, Powell LV. Oral health and medical status in dentate low-income older persons. Special care in dentistry : official publication of the American Association of Hospital Dentists, the Academy of Dentistry for the Handicapped, and the American Society for Geriatric Dentistry. 1998;18(2):70-7. Epub 1998/07/29. PubMed PMID: 9680914.

454. Rheu GB, Ji S, Ryu JJ, Lee JB, Shin C, Lee JY, et al. Risk assessment for clinical attachment loss of periodontal tissue in Korean adults. The journal of advanced prosthodontics. 2011;3(1):25-32. Epub 2011/04/20. doi: 10.4047/jap.2011.3.1.25. PubMed PMID: 21503190; PubMed Central PMCID: PMCPMC3076570.

455. Spangler L, Reid RJ, Inge R, Newton KM, Hujoel P, Chaudhari M, et al. Cross-sectional study of periodontal care and Glycosylated Hemoglobin in an insured population. Diabetes care. 2010;33(8):1753-8. Epub 2010/05/28. doi: 10.2337/dc09-1412. PubMed PMID: 20504894; PubMed Central PMCID: PMCPMC2909057.

456. Timonen P, Saxlin T, Knuuttila M, Suominen AL, Jula A, Tervonen T, et al. Role of insulin sensitivity and beta cell function in the development of periodontal disease in adults without diabetes. Journal of clinical periodontology. 2013;40(12):1079-86. Epub 2013/11/07. doi: 10.1111/jcpe.12162. PubMed PMID: 24192072.

457. Timonen P, Suominen-Taipale L, Jula A, Niskanen M, Knuuttila M, Ylostalo P. Insulin sensitivity and periodontal infection in a non-diabetic, non-smoking adult population. Journal of clinical periodontology. 2011;38(1):17-24. Epub 2010/11/13. doi: 10.1111/j.1600-051X.2010.01642.x. PubMed PMID: 21070326.

458. Xiong X, Elkind-Hirsch KE, Xie Y, Delarosa R, Maney P, Pridjian G, et al. Periodontal disease as a potential risk factor for the development of diabetes in women with a prior history of gestational diabetes mellitus. Journal of public health dentistry. 2013;73(1):41-9. Epub 2012/12/12. doi: 10.1111/jphd.12004. PubMed PMID: 23215856.

459. Yamamoto T, Tsuneishi M, Furuta M, Ekuni D, Morita M, Hirata Y. Relationship between decrease of erythrocyte count and progression of periodontal disease in a rural Japanese population. Journal of periodontology. 2011;82(1):106-13. Epub 2010/08/05. doi: 10.1902/jop.2010.100211. PubMed PMID: 20681815.

460. Alyasiry AM. Oral Hygiene For The Diabetes Mellitus And Osteoporosis Patients. Research Journal of Pharmaceutical Biological and Chemical Sciences. 2017;8(3):783-91. PubMed PMID: WOS:000410665400086.

461. Baig MS, Bhutto RA, Badar S, Ali S. Oral Hygiene Practices and Periodontal diseases in type 2 Diabetes Mellitus Patients. Pakistan Journal of Medical & Health Sciences. 2015;9(4):1220-3. PubMed PMID: WOS:000422081900028.

462. D'Aiuto F, Gkranias N, Bhowruth D, Khan T, Orlandi M, Suvan J, et al. Systemic effects of periodontitis treatment in patients with type 2 diabetes: a 12 month, single-centre, investigator-masked, randomised trial. The lancet Diabetes & endocrinology. 2018;6(12):954-65. Epub 2018/11/27. doi: 10.1016/s2213-8587(18)30038-x. PubMed PMID: 30472992.

463. Izuora KE, Ezeanolue EE, Neubauer MF, Gewelber CL, Allenback GL, Umpierrez GE. Dental loss among ambulatory patients with diabetes. Journal of Clinical and Translational Endocrinology. 2016;4:28-31. doi: 10.1016/j.jcte.2016.02.003. PubMed PMID: WOS:000399090200005.

464. Khader Y, Khassawneh B, Obeidat B, Hammad M, El-Salem K, Bawadi H, et al. Periodontal Status of Patients With Metabolic Syndrome Compared to Those Without Metabolic Syndrome. Journal of Periodontology. 2008;79(11):2048-53. doi: 10.1902/jop.2008.080022. PubMed PMID: WOS:000260933900008.

465. Maia FB, de Sousa ET, Sampaio FC, Freitas CH, Forte FD. Tooth loss in middle-aged adults with diabetes and hypertension: Social determinants, health perceptions, oral impact on daily performance (OIDP) and treatment need. Medicina oral, patologia oral y cirugia bucal. 2018;23(2):e203-e10. Epub 2018/02/25. doi: 10.4317/medoral.22176. PubMed PMID: 29476679; PubMed Central PMCID: PMCPMC5911361.

466. Neely AL, Holford TR, Loe H, Anerud A, Boysen H. The natural history of periodontal disease in man. Risk factors for progression of attachment loss in individuals receiving no oral health care. Journal of Periodontology. 2001;72(8):1006-15. doi: 10.1902/jop.2001.72.8.1006. PubMed PMID: WOS:000170546900004.

467. Riaz S, Habib K, Sajid M. Analysis of Relationship among Obesity, Diabetes Mellitus and Periodontitis in Local Population of Pakistan. Indo American Journal of Pharmaceutical Sciences. 2018;5(12):17070-3. doi: 10.5281/zenodo.2527588. PubMed PMID: WOS:000454558500403.

468. Shaheen A, Bashir M, Ashraf M, Iqbal S, Khattak S, Ali R. Periodontitis and Blood Glucose Level in Obese and Non Obese Type-2 Diabetic Patients. Pakistan Journal of Medical & Health Sciences. 2017;11(4):1436-7. PubMed PMID: WOS:000432248000061.

469. Susin C, Haas AN, Valle PM, Oppermann RV, Albandar JM. Prevalence and risk indicators for chronic periodontitis in adolescents and young adults in south Brazil. Journal of Clinical Periodontology. 2011;38(4):326-33. doi: 10.1111/j.1600-051X.2011.01699.x. PubMed PMID: WOS:000287700700004.

470. Xie QF, Ainamo A. Association of edentulousness with systemic factors in elderly people living at home. Community Dentistry and Oral Epidemiology. 1999;27(3):202-9. doi: 10.1111/j.1600-0528.1999.tb02011.x. PubMed PMID: WOS:000080761900006.

471. Zhao D, Zhen Z, Pelekos G, Yiu KH, Jin L. Periodontal disease increases the risk for onset of systemic comorbidities in dental hospital attendees: An 18-year retrospective cohort study. Journal of periodontology. 2019;90(3):225-33. Epub 2018/10/09. doi: 10.1002/jper.18-0224. PubMed PMID: 30296334.

472. Cabrera C, Hakeberg M, Ahlqwist M, Wedel H, Bjorkelund C, Bengtsson C, et al. Can the relation between tooth loss and chronic disease be explained by socio-economic status? A 24-year follow-up from the population study of women in Gothenburg, Sweden. European journal of epidemiology. 2005;20(3):229-36. Epub 2005/06/01. PubMed PMID: 15921040.

473. Rangel-Nieto IG, Borges-Yanez SA, Jimenez-Corona ME, Jimenez-Corona L, Jimenez-Corona A. Prevalence of Periodontitis in Adult Population with Prediabetes and Type 2 Diabetes: The Comitan Study. Diabetes. 2014;63:A642-A. PubMed PMID: WOS:000359481603385.

474. Oluwagbemigun K, Dietrich T, Pischon N, Bergmann M, Boeing H. Association between Number of Teeth and Chronic Systemic Diseases: A Cohort Study Followed for 13 Years. PloS one. 2015;10(5). doi: 10.1371/journal.pone.0123879. PubMed PMID: WOS:000354049700023.

475. Liljestrand JM, Havulinna AS, Paju S, Mannisto S, Salomaa V, Pussinen PJ. Missing Teeth Predict Incident Cardiovascular Events, Diabetes, and Death. Journal of dental

research. 2015;94(8):1055-62. Epub 2015/05/21. doi: 10.1177/0022034515586352. PubMed PMID: 25991651.

476. Al-Mubarak S, Ciancio S, Aljada A, Mohanty P, Ross C, Dandona P. Comparative evaluation of adjunctive oral irrigation in diabetics. Journal of clinical periodontology. 2002;29(4):295-300. Epub 2002/04/23. PubMed PMID: 11966926.

477. Andriankaja OM, Joshipura K. Potential association between prediabetic conditions and gingival and/or periodontal inflammation. Journal of diabetes investigation. 2014;5(1):108-14. Epub 2014/04/15. doi: 10.1111/jdi.12122. PubMed PMID: 24729853; PubMed Central PMCID: PMCPMC3980950.

478. Dahiya P, Kamal R, Gupta R. Obesity, periodontal and general health: Relationship and management. Indian journal of endocrinology and metabolism. 2012;16(1):88-93. Epub 2012/01/26. doi: 10.4103/2230-8210.91200. PubMed PMID: 22276257; PubMed Central PMCID: PMCPMC3263203.

479. Demmer RT, Breskin A, Rosenbaum M, Zuk A, LeDuc C, Leibel R, et al. The subgingival microbiome, systemic inflammation and insulin resistance: The Oral Infections, Glucose Intolerance and Insulin Resistance Study. Journal of clinical periodontology. 2017;44(3):255-65. Epub 2016/12/16. doi: 10.1111/jcpe.12664. PubMed PMID: 27978598; PubMed Central PMCID: PMCPMC5328907.

480. Javed F, Thafeed Alghamdi AS, Mikami T, Mehmood A, Ahmed HB, Samaranayake LP, et al. Effect of glycemic control on self-perceived oral health, periodontal parameters, and alveolar bone loss among patients with prediabetes. Journal of periodontology. 2014;85(2):234-41. Epub 2013/05/11. doi: 10.1902/jop.2013.130008. PubMed PMID: 23659422.

481. Karikoski A, Murtomaa H. Periodontal treatment needs in a follow-up study among adults with diabetes in Finland. Acta odontologica Scandinavica. 2003;61(1):6-10. Epub 2003/03/15. PubMed PMID: 12635774.

482. Kumar MS, Vamsi G, Sripriya R, Sehgal PK. Expression of matrix metalloproteinases (MMP-8 and -9) in chronic periodontitis patients with and without diabetes mellitus. Journal of periodontology. 2006;77(11):1803-8. Epub 2006/11/02. doi: 10.1902/jop.2006.050293. PubMed PMID: 17076603.

483. Mawardi HH, Elbadawi LS, Sonis ST. Current understanding of the relationship between periodontal and systemic diseases. Saudi medical journal. 2015;36(2):150-8. Epub 2015/02/27. doi: 10.15537/smj.2015.2.9424. PubMed PMID: 25719577; PubMed Central PMCID: PMCPMC4375690.

484. Merchant AT, Georgantopoulos P, Howe CJ, Virani SS, Morales DA, Haddock KS. Effect of Long-Term Periodontal Care on Hemoglobin A1c in Type 2 Diabetes. Journal of dental research. 2016;95(4):408-15. Epub 2015/12/25. doi: 10.1177/0022034515622197. PubMed PMID: 26701348; PubMed Central PMCID: PMCPMC4802779.

485. Sastrowijoto SH, van der Velden U, van Steenbergen TJ, Hillemans P, Hart AA, de Graaff J, et al. Improved metabolic control, clinical periodontal status and subgingival microbiology in insulin-dependent diabetes mellitus. A prospective study. Journal of clinical periodontology. 1990;17(4):233-42. Epub 1990/04/01. PubMed PMID: 2189897.

486. Silva NSV, da Silva LA, Jaluul O, Jacob-Filho W, Siqueira S. Oral infections, comorbidities and sensory evidences in elderly: Cross-sectional study. Archives of

gerontology and geriatrics. 2017;73:15-20. Epub 2017/07/21. doi: 10.1016/j.archger.2017.06.011. PubMed PMID: 28728082.

487. Suzuki S, Yoshino K, Takayanagi A, Ishizuka Y, Satou R, Kamijo H, et al. Comparison of risk factors for tooth loss between professional drivers and white-collar workers: an internet survey. Industrial health. 2016;54(3):246-53. Epub 2016/01/05. doi: 10.2486/indhealth.2015-0207. PubMed PMID: 26726831; PubMed Central PMCID: PMCPMC4939861.

488. Eklund SA, Burt BA. Risk-Factors for Total Tooth Loss in the United-States - Longitudinal Analysis of National Data. Journal of Public Health Dentistry. 1994;54(1):5-14. doi: 10.1111/j.1752-7325.1994.tb01173.x. PubMed PMID: WOS:A1994MZ50700002.

489. Gheorghe DN, Surlin P, Herascu E, Vere C, Cojocaru M, Iliescu AA, et al. Metabolic Status of Periodontal Patients with Systemic Diseases. Romanian Journal of Oral Rehabilitation. 2017;9(4):105-19. PubMed PMID: WOS:000424297400019.

490. Kesic L, Radovic S, Dedic A, Avdic M, Obradovic R. The influence of HbA1c level on gingival inflammation and periodontal therapy among diabetic patients. Healthmed. 2011;5(1):200-4. PubMed PMID: WOS:000287819600027.

491. Alzogibi A, Al-Shubrumi F, Bhattacharjee S, Prabhu V. Awareness of the Association between Periodontal Health and Diabetes Mellitus among Middle-Aged Population in Burridah, Saudi Arabia. International Journal of Life Science and Pharma Research. 2020;10(1):L1-L6. doi: 10.22376/ijpbs/lpr.2020.10.1.L1-6. PubMed PMID: WOS:000528223800001.

492. dos Santos RC, Pinho RCM, Cimoes R. Chronic Periodontitis in Patients with Type 2 Diabetes: Analysis of the FokI Polymorphism and Perception of Quality of Life. Pesquisa Brasileira Em Odontopediatria E Clinica Integrada. 2020;20. doi: 10.1690/pboci.2020.149. PubMed PMID: WOS:000556101400001.

493. Kuwamura Y, Sakamoto E, Sumikawa M, Matsuhisa M, Hinode D, Uemura H, et al. Assessing the oral health of in-patients with diabetes using a clinical version of the Diabetes Oral Health Assessment Tool© and its association with dental examinations. J Med Invest. 2019;66(3.4):328-36. Epub 2019/10/28. doi: 10.2152/jmi.66.328. PubMed PMID: 31656299.

494. Laukkanen E, Vehkalahti MM, Kotiranta AK. Impact of systemic diseases and tooth-based factors on outcome of root canal treatment. Int Endod J. 2019;52(10):1417-26. Epub 2019/05/11. doi: 10.1111/iej.13143. PubMed PMID: 31074887.

495. Peceliuniene J, Zukauskaite I, Sadauskaite N, Norkus A. Diabetes Mellitus and Periodontitis: Lower Income - More Complicated Course of Disease? Annals of Medical and Health Sciences Research. 2020;10(5):1060-6. PubMed PMID: WOS:000607198600010.

496. Saxlin T, Ylöstalo P, Suominen-Taipale L, Männistö S, Knuuttila M. Association between periodontal infection and obesity: results of the Health 2000 Survey. J Clin Periodontol. 2011;38(3):236-42. Epub 2011/01/05. doi: 10.1111/j.1600-051X.2010.01677.x. PubMed PMID: 21198765.

497. Shearer DM, Thomson WM, Cameron CM, Ramrakha S, Wilson G, Wong TY, et al. Periodontitis and multiple markers of cardiometabolic risk in the fourth decade: A cohort study. Community Dent Oral Epidemiol. 2018;46(6):615-23. Epub 2018/08/31. doi: 10.1111/cdoe.12414. PubMed PMID: 30160305; PubMed Central PMCID: PMCPMC6237628.

498. Toda K, Mizutani K, Minami I, Ye M, Arakawa T, Mitsubayashi K, et al. Effects of oral health instructions on glycemic control and oral health status of periodontitis patients with type 2 diabetes mellitus: A preliminary observation. J Dent Sci. 2019;14(2):171-7. Epub 2019/06/19. doi: 10.1016/j.jds.2019.01.009. PubMed PMID: 31210891; PubMed Central PMCID: PMCPMC6562110.

499. Chavarry NG, Vettore MV, Sansone C, Sheiham A. The relationship between diabetes mellitus and destructive periodontal disease: a meta-analysis. Oral health & preventive dentistry. 2009;7(2):107-27. Epub 2009/07/09. PubMed PMID: 19583037.

500. Darre L, Vergnes JN, Gourdy P, Sixou M. Efficacy of periodontal treatment on glycaemic control in diabetic patients: A meta-analysis of interventional studies. Diabetes & metabolism. 2008;34(5):497-506. Epub 2008/10/25. doi: 10.1016/j.diabet.2008.03.006. PubMed PMID: 18948050.

501. Engebretson S, Kocher T. Evidence that periodontal treatment improves diabetes outcomes: a systematic review and meta-analysis. Journal of periodontology. 2013;84(4 Suppl):S153-69. Epub 2013/05/03. doi: 10.1902/jop.2013.1340017. PubMed PMID: 23631575; PubMed Central PMCID: PMCPMC4100543.

502. Graziani F, Gennai S, Solini A, Petrini M. A systematic review and meta-analysis of epidemiologic observational evidence on the effect of periodontitis on diabetes An update of the EFP-AAP review. Journal of clinical periodontology. 2018;45(2):167-87. Epub 2017/12/27. doi: 10.1111/jcpe.12837. PubMed PMID: 29277926.

503. Khader YS, Dauod AS, El-Qaderi SS, Alkafajei A, Batayha WQ. Periodontal status of diabetics compared with nondiabetics: a meta-analysis. Journal of diabetes and its complications. 2006;20(1):59-68. Epub 2006/01/04. doi: 10.1016/j.jdiacomp.2005.05.006. PubMed PMID: 16389170.

504. Nascimento GG, Leite FRM, Vestergaard P, Scheutz F, Lopez R. Does diabetes increase the risk of periodontitis? A systematic review and meta-regression analysis of longitudinal prospective studies. Acta diabetologica. 2018;55(7):653-67. Epub 2018/03/05. doi: 10.1007/s00592-018-1120-4. PubMed PMID: 29502214.

505. Ziukaite L, Slot DE, Van der Weijden FA. Prevalence of diabetes mellitus in people clinically diagnosed with periodontitis: A systematic review and meta-analysis of epidemiologic studies. Journal of clinical periodontology. 2018;45(6):650-62. Epub 2017/11/11. doi: 10.1111/jcpe.12839. PubMed PMID: 29125699.

506. Hsu YT, Nair M, Angelov N, Lalla E, Lee CT. Impact of diabetes on clinical periodontal outcomes following non-surgical periodontal therapy. Journal of clinical periodontology. 2019;46(2):206-17. Epub 2018/12/12. doi: 10.1111/jcpe.13044. PubMed PMID: 30536853.

507. Diabetes and Periodontitis are mutually dependent. Diabetes Stoffwechsel Und Herz. 2020;29(3):196-. PubMed PMID: WOS:000541415500015.

508. Badiger AB, Gowda TM, Chandra K, Mehta DS. Bilateral Interrelationship of Diabetes and Periodontium. Current Diabetes Reviews. 2019;15(5):357-62. doi: 10.2174/1573399815666190115144534. PubMed PMID: WOS:000482174000003.

509. Felton DA. Edentulism and Comorbid Factors. Journal of Prosthodontics-Implant Esthetic and Reconstructive Dentistry. 2009;18(2):88-96. doi: 10.1111/j.1532-849X.2009.00437.x. PubMed PMID: WOS:000207914200002.

510. Genco RJ, Graziani F, Hasturk H. Effects of periodontal disease on glycemic control, complications, and incidence of diabetes mellitus. Periodontol 2000. 2020;83(1):59-65. Epub 2020/05/10. doi: 10.1111/prd.12271. PubMed PMID: 32385875.

511. Glurich I, Acharya A. Updates from the Evidence Base Examining Association between Periodontal Disease and Type 2 Diabetes Mellitus: Current Status and Clinical Relevance. Curr Diab Rep. 2019;19(11):121. Epub 2019/11/07. doi: 10.1007/s11892-019-1228-0. PubMed PMID: 31696343.

512. Jain A, Chawla M, Kumar A, Chawla R, Grover V, Ghosh S, et al. Management of periodontal disease in patients with diabetes good clinical practice guidelines: A joint statement by Indian Society of Periodontology and Research Society for the Study of Diabetes in India. International Journal of Diabetes in Developing Countries. doi: 10.1007/s13410-020-00897-2. PubMed PMID: WOS:000595365500001.

513. Liccardo D, Cannavo A, Spagnuolo G, Ferrara N, Cittadini A, Rengo C, et al. Periodontal Disease: A Risk Factor for Diabetes and Cardiovascular Disease. Int J Mol Sci. 2019;20(6). Epub 2019/03/23. doi: 10.3390/ijms20061414. PubMed PMID: 30897827; PubMed Central PMCID: PMCPMC6470716.

514. Engebretson S. Periodontal disease and glycemic control in diabetics. Evidence-based dentistry. 2014;15(3):93-4. Epub 2014/10/25. doi: 10.1038/sj.ebd.6401040. PubMed PMID: 25343401.

515. Borgnakke WS, Glick M, Genco RJ. Periodontitis: the canary in the coal mine. Journal of the American Dental Association (1939). 2013;144(7):764-6. Epub 2013/07/03. PubMed PMID: 23813251.

516. Mealey B. Diabetes and periodontal diseases. Journal of periodontology. 1999;70(8):935-49. Epub 1999/09/07. doi: 10.1902/jop.1999.70.8.935. PubMed PMID: 10476904.

517. Scannapieco FA. Position paper of The American Academy of Periodontology: periodontal disease as a potential risk factor for systemic diseases. Journal of periodontology. 1998;69(7):841-50. Epub 1998/08/26. PubMed PMID: 9706864.

518. Studies show link between diabetes, severity of periodontal disease. Journal of the American Dental Association (1939). 1993;124(9):24. Epub 1993/09/01. PubMed PMID: 8409022.

519. Periodontal disease increases risk of diabetes and chronic lung disease. Journal of the American Dental Association (1939). 1999;130(5):628. Epub 1999/05/20. PubMed PMID: 10332125.

520. Dentists play important role in early detection of diabetes. Dentistry today. 2003;22(6):32. Epub 2003/07/10. PubMed PMID: 12847834.

521. Periodontal disease-type 2 diabetes link confirmed. British dental journal. 2010;208(10):447. Epub 2010/05/22. doi: 10.1038/sj.bdj.2010.495. PubMed PMID: 20489763.

522. Akazawa H. Periodontitis and Diabetes Mellitus: Be true to your teeth. International heart journal. 2018;59(4):680-2. Epub 2018/08/03. doi: 10.1536/ihj.18-410. PubMed PMID: 30068836.

523. Finney LS, Finney MO, Gonzalez-Campoy JM. What the mouth has to say about diabetes. Careful examinations can avert serious complications. Postgraduate medicine. 1997;102(6):117-26. Epub 1997/12/24. doi: 10.3810/pgm.1997.12.384. PubMed PMID: 9406568.

524. Genco RJ. Periodontal disease and association with diabetes mellitus and diabetes: clinical implications. Journal of dental hygiene : JDH. 2009;83(4):186-7. Epub 2009/11/17. PubMed PMID: 19909640.

525. Aichelmann-Reidy MB. Probing depth but not attachment level may be associated with the development of impaired glucose tolerance among (40- to 79-year-old) Hisayama residents. J Evid Based Dent Pract. 2005;5(4):231-3. Epub 2006/12/02. doi: 10.1016/j.jebdp.2005.10.002. PubMed PMID: 17138385.

526. Alrahlah A, Abduljabbar T, Vohra F. Periodontal parameters in prediabetes, type 2 diabetes mellitus, and non-diabetic patients (vol 32, e81, 2018). Brazilian Oral Research. 2018;32. doi: 10.1590/1807-3107bor-2018.vol32.0081err. PubMed PMID: WOS:000467761300001.

527. Chang Y, Lee JS, Lee KJ, Woo HG, Song TJ. Improved oral hygiene is associated with decreased risk of new-onset diabetes: a nationwide population-based cohort study. Diabetologia. 2020;63(5):924-33. Epub 2020/03/05. doi: 10.1007/s00125-020-05112-9. PubMed PMID: 32128623.

528. Kim YT, Choi JK, Kim DH, Jeong SN, Lee JH. Association between health status and tooth loss in Korean adults: longitudinal results from the National Health Insurance Service-Health Examinee Cohort, 2002-2015. J Periodontal Implant Sci. 2019;49(3):158-70. Epub 2019/07/10. doi: 10.5051/jpis.2019.49.3.158. PubMed PMID: 31285940; PubMed Central PMCID: PMCPMC6599754.

529. Lee JH, Jeong SN. A Population-Based Study on the Association between Periodontal Disease and Major Lifestyle-Related Comorbidities in South Korea: An Elderly Cohort Study from 2002-2015. Medicina (Kaunas). 2020;56(11). Epub 2020/11/04. doi: 10.3390/medicina56110575. PubMed PMID: 33138320; PubMed Central PMCID: PMCPMC7693625.

530. Adam HS, Zhang SP, Philips K, Moss K, Wu D, Selvin E, et al. Periodontal Disease is Associated With Risk of Incident Diabetes Among Non-obese Individuals. Circulation. 2020;141. doi: 10.1161/circ.141.suppl_1.P442. PubMed PMID: WOS:000589965800410.

531. Lau P, Gupta N, Martin R, McPherson M, Calache H. Diabetes and Oral Health (DiabOH): validating a periodontal risk assessment tool for non-dental practitioners. Australian Journal of Primary Health. 2019;25(3):XVIII-XVIII. PubMed PMID: WOS:000474928700025.

532. Alshihayb TS, Kaye EA, Zhao Y, Leone CW, Heaton B. A quantitative bias analysis to assess the impact of unmeasured confounding on associations between diabetes and periodontitis. Journal of clinical periodontology. 2021;48(1):51-60. Epub 2020/10/09. doi: 10.1111/jcpe.13386. PubMed PMID: 33031608.

533. Chiu SY, Lai H, Yen AM, Fann JC, Chen LS, Chen HH. Temporal sequence of the bidirectional relationship between hyperglycemia and periodontal disease: a community-based study of 5,885 Taiwanese aged 35-44 years (KCIS No. 32). Acta diabetologica. 2015;52(1):123-31. Epub 2014/07/06. doi: 10.1007/s00592-014-0612-0. PubMed PMID: 24990094.

534. Jimenez M, Hu FB, Marino M, Li Y, Joshipura KJ. Type 2 diabetes mellitus and 20 year incidence of periodontitis and tooth loss. Diabetes research and clinical practice. 2012;98(3):494-500. Epub 2012/10/09. doi: 10.1016/j.diabres.2012.09.039. PubMed PMID: 23040240; PubMed Central PMCID: PMCPMC3551264.

535. Morita I, Inagaki K, Nakamura F, Noguchi T, Matsubara T, Yoshii S, et al. Relationship between periodontal status and levels of glycated hemoglobin. Journal of dental research. 2012;91(2):161-6. Epub 2011/12/14. doi: 10.1177/0022034511431583. PubMed PMID: 22157098.

536. Lee JH, Choi JK, Jeong SN, Choi SH. Charlson comorbidity index as a predictor of periodontal disease in elderly participants. Journal of periodontal & implant science. 2018;48(2):92-102. Epub 2018/05/18. doi: 10.5051/jpis.2018.48.2.92. PubMed PMID: 29770238; PubMed Central PMCID: PMCPMC5944227.

537. Sun KT, Chen SC, Lin CL, Hsu JT, Chen IA, Wu IT, et al. The association between Type 1 diabetes mellitus and periodontal diseases. Journal of the Formosan Medical Association = Taiwan yi zhi. 2018. Epub 2018/11/06. doi: 10.1016/j.jfma.2018.10.012. PubMed PMID: 30391109.

538. Lin SY, Lin CL, Liu JH, Wang IK, Hsu WH, Chen CJ, et al. Association between periodontitis needing surgical treatment and subsequent diabetes risk: a population-based cohort study. Journal of periodontology. 2014;85(6):779-86. Epub 2013/10/23. doi: 10.1902/jop.2013.130357. PubMed PMID: 24144269.

539. Demmer RT, Jacobs DR, Jr., Desvarieux M. Periodontal disease and incident type 2 diabetes: results from the First National Health and Nutrition Examination Survey and its epidemiologic follow-up study. Diabetes care. 2008;31(7):1373-9. Epub 2008/04/09. doi: 10.2337/dc08-0026. PubMed PMID: 18390797; PubMed Central PMCID: PMCPMC2453650.

540. Ide R, Hoshuyama T, Wilson D, Takahashi K, Higashi T. Periodontal disease and incident diabetes: a seven-year study. Journal of dental research. 2011;90(1):41-6. Epub 2010/11/03. doi: 10.1177/0022034510381902. PubMed PMID: 21041549.

541. Miyawaki A, Toyokawa S, Inoue K, Miyoshi Y, Kobayashi Y. Self-Reported Periodontitis and Incident Type 2 Diabetes among Male Workers from a 5-Year Follow-Up to MY Health Up Study. PloS one. 2016;11(4):e0153464. Epub 2016/04/27. doi: 10.1371/journal.pone.0153464. PubMed PMID: 27115749; PubMed Central PMCID: PMCPMC4846029.

542. Kebede TG, Pink C, Rathmann W, Kowall B, Volzke H, Petersmann A, et al. Does periodontitis affect diabetes incidence and haemoglobin A1c change? An 11-year follow-up study. Diabetes & metabolism. 2018;44(3):243-9. Epub 2017/12/19. doi: 10.1016/j.diabet.2017.11.003. PubMed PMID: 29249612.

543. Myllymaki V, Saxlin T, Knuuttila M, Rajala U, Keinanen-Kiukaanniemi S, Anttila S, et al. Association between periodontal condition and the development of type 2 diabetes mellitus - results from a 15-year follow-up study. Journal of clinical periodontology. 2018. Epub 2018/08/23. doi: 10.1111/jcpe.13005. PubMed PMID: 30133880.

544. Winning L, Patterson CC, Neville CE, Kee F, Linden GJ. Periodontitis and incident type 2 diabetes: a prospective cohort study. Journal of clinical periodontology. 2017;44(3):266-74. Epub 2016/12/31. doi: 10.1111/jcpe.12691. PubMed PMID: 28036104.

545. Lee JH, Oh JY, Youk TM, Jeong SN, Kim YT, Choi SH. Association between periodontal disease and non-communicable diseases: A 12-year longitudinal health-

examinee cohort study in South Korea. Medicine. 2017;96(26):e7398. Epub 2017/06/29. doi: 10.1097/md.0000000000007398. PubMed PMID: 28658175; PubMed Central PMCID: PMCPMC5500097.

546. Lee CY, Kuan YH, Tsai YF, Tai CJ, Tsai TH, Huang KH. Correlation between diabetes mellitus and periodontitis in Taiwan: A nationwide cohort study. Diabetes research and clinical practice. 2019;150:245-52. Epub 2019/03/19. doi: 10.1016/j.diabres.2019.03.019. PubMed PMID: 30880092.
